# Factors that contribute to the success of primary isolation of Mycoplasma genitalium from clinical samples

**DOI:** 10.1099/jmm.0.002040

**Published:** 2025-07-03

**Authors:** Jose L. Huaman, Catriona S. Bradshaw, Teck-Phui Chua, Erica L. Plummer, Jennifer A. Danielewski, Lenka A. Vodstrcil, Jorgen S. Jensen, Suzanne M. Garland, Natasha Wild, Gerald L. Murray

**Affiliations:** 1 Department of Obstetrics, Gynaecology and Newborn Health, University of Melbourne, Parkville, Victoria, Australia; 2 Centre for Women’s Infectious Diseases, The Royal Women’s Hospital, Parkville, Victoria, Australia; 3 Molecular Microbiology Research Group, Murdoch Children’s Research Institute, Parkville, Victoria, Australia; 4 Melbourne Sexual Health Centre, Alfred Health, Carlton, Victoria, Australia; 5 Central Clinical School, Monash University, Melbourne, Victoria, Australia; 6 Centre for Epidemiology and Biostatistics, Melbourne School of Population and Global Health, University of Melbourne, Parkville, Victoria, Australia; 7 Research Unit for Reproductive Microbiology, Statens Serum Institut, Copenhagen, Denmark

**Keywords:** bacterial load, culture, *Mycoplasma genitalium*, primary isolation

## Abstract

**Introduction.** *Mycoplasma genitalium*, a small and slow-growing bacterium, has gained notoriety due to rapidly increasing rates of antibiotic resistance.

**Gap Statement.** *M. genitalium* is difficult to culture, limiting efforts to understand its biology and the mechanisms of antimicrobial resistance.

**Aim.** To understand factors that influence success in primary isolation of *M. genitalium* from clinical samples.

**Methodology.** Neat urine or swabs (high vaginal and anal, in universal transport medium) were collected from patients with confirmed or suspected *M. genitalium* infections attending the Melbourne Sexual Health Centre. The specimens were stored at −80 °C prior to laboratory analysis. Initial diagnosis was by transcription-mediated amplification (TMA) assay, and samples subsequently testing positive by quantitative PCR (qPCR) were washed twice and inoculated into Vero cell monolayers with a selective antibiotic mixture (cycloheximide and Thayer-Martin Medium I). Cultures were incubated at 37 °C with 5% CO_2_ for 8 weeks and observed daily, with qPCR used to monitor growth.

**Results.** In total, 127 TMA-positive samples were subjected to qPCR, and *M. genitalium* genomic DNA (gDNA) was detected in 53.5% (68/127) of these samples. An isolate was obtained from 26.5% (18/68) of the gDNA-positive samples following co-culture with Vero cells. The isolation rate varied between sample types, with growth detected in 12.5% (3/24) of the high vaginal swabs and 37.5% (15/40) of the urine samples. No isolates were obtained from anal swabs. The proportion with a successful culture was influenced by the initial *M. genitalium* load in the sample, which translated into the inoculum size for the Vero cell monolayer. Isolation was unsuccessful with low inoculum (<2,000 genome equivalents, geq), partially successful (13.3%) with a moderate inoculum (2,500–9,500 geq) and highly successful (100%) in samples with a high inoculum (>10,000 geq).

**Conclusion.** The initial bacterial load emerged as a critical determinant of isolation success. This emphasizes the importance of optimizing sample collection and *M. genitalium* isolation procedures.

## Introduction

*Mycoplasma genitalium*, a sexually transmitted bacterium, has been associated with an increased risk of pelvic inflammatory disease [1], low birthweight [2], cervicitis in women [3] and urethritis in men [4,5]. This micro-organism has gained notoriety due to rapidly increasing resistance to antibiotic treatments [6]. Moreover, the prevalence for resistance markers to first- and second-line antibiotics (macrolides and fluoroquinolones, respectively) is increasing globally [7,8].

*M. genitalium* is a slow-growing micro-organism with the smallest known bacterial genome; 580 kbps code for fewer than 500 genes [9,10]. In 1996, Jensen *et al*. [11] introduced the use of Vero cells for the initial co-cultivation of *M. genitalium* to facilitate recovery from clinical samples. However, isolating this bacterium from clinical specimens remains challenging and time-consuming, with few laboratories worldwide performing this task.

Testing for *M. genitalium* infection is performed exclusively using molecular assays. The Aptima^®^ *Mycoplasma genitalium* assay (Hologic Inc.) is a transcription-mediated amplification (TMA) test that targets the rRNA of *M. genitalium* which is present in higher copy numbers compared to DNA molecules [12,13]. TMA exhibits higher analytical sensitivity compared to DNA-based detection methods, such as quantitative PCR (qPCR), due to its ability to amplify targets of increased abundance [12,14].

Since direct assessment of antibiotic susceptibility of *M. genitalium* is not routinely conducted, our understanding of this bacterium is primarily based upon molecular analysis of resistance-associated genes. Moreover, the quantity of *M. genitalium* genetic material in clinical samples is typically insufficient for direct sequencing. Coupled with the difficulties of culture, this has resulted in a limited understanding of the genomics of this micro-organism. Consequently, comprehending the factors that influence the success of primary isolation of *M. genitalium* from clinical samples is crucial for enhancing resistance screening and expanding our knowledge of this bacterium.

In this study, we assessed the factors that influenced success in primary isolation of *M. genitalium* from clinical specimens.

## Methods

### Clinical specimens

This study employed specimens from patients with suspected or confirmed *M. genitalium* infections who attended the Melbourne Sexual Health Centre (Melbourne, Australia) between September 2022 and October 2024. To maximize the likelihood of obtaining viable bacterial cells from the collected samples, patient inclusion was based on their recent antibiotic treatment history. Eligible participants were either treatment-naïve individuals who had not received any prior antibiotic therapy for *M. genitalium* infection or those who had completed their treatment regimen at least 14 days before sample collection.

**Fig. 1.**
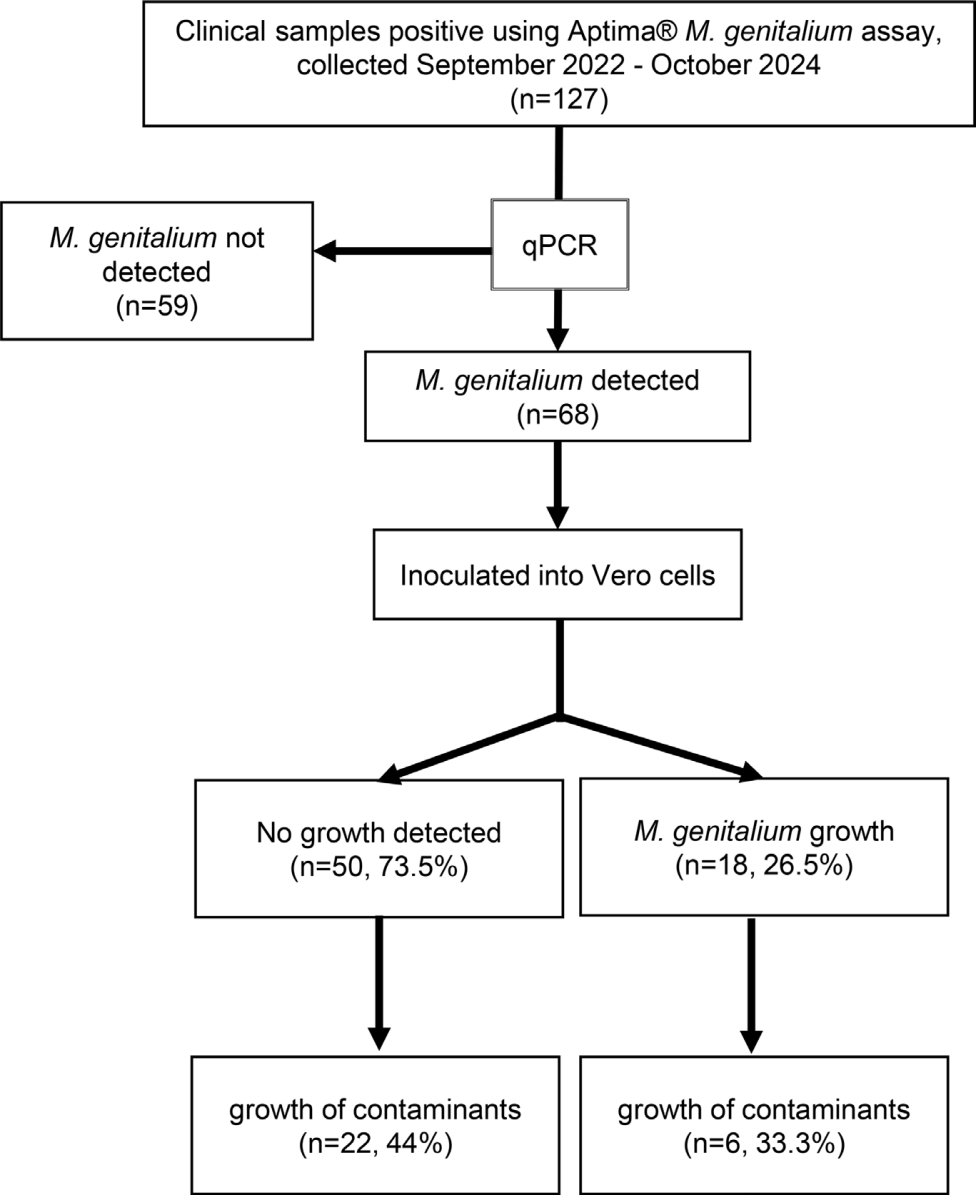
Overview of sample collection, sample quality check and Vero cell co-culture results.

Samples were taken simultaneously for diagnosis (Aptima^®^ *Mycoplasma genitalium* TMA Assay – Hologic Inc., San Diego, CA, USA) and for culture (either neat urine, high vaginal swabs or anal swabs). Clinician-collected high vaginal (samples collected closer to the cervix) and anal swab specimens were obtained using flocked swabs and placed immediately into a 1 ml transport media tube (UTM Copan, Brescia, Italy). Urine specimens were collected from participants using sterile 70 ml sample containers. Between 10 and 14 ml of each urine sample was aliquoted into a sterile 15 ml polypropylene conical tube. Each specimen was stored at −80 °C on site until delivery to the Royal Women’s Hospital laboratory for analysis. Samples for which *M. genitalium* was detected by TMA underwent the workflow outlined in Fig. 1.

### Maintenance of Vero cells

Vero cells were maintained in Eagle’s minimum essential medium (EMEM) (Sigma-Aldrich, MO, USA; Cat# M5650-500ML) supplemented with 2% Ultroser G serum substitute (Sartorius, Cergy-Saint-Christophe, France) and 1% l-glutamine (Gibco, Thermo Fisher Scientific, Waltham, MA, USA), without the addition of antibiotics, at 37 °C in a humidified atmosphere with 5% CO_2_ [15]. Vero cells were propagated in a vented cap T-25 cell culture flask (Corning Inc., Corning, NY, USA).

### Isolation of *M. genitalium* from clinical specimens in Vero cells

The methodology for isolation of *M. genitalium* was performed as described previously [11,16, 17], with minor modifications as indicated below. Briefly, 4 ml of neat urine or 1 ml of swab transport media was centrifuged at 20,000 ***g*** for 30 min at 4 °C. The resulting pellets were resuspended in 1 ml of EMEM. Subsequently, the resuspended pellets were centrifuged again, and the final pellets were resuspended in 0.5 ml of fresh EMEM. Chelex 100 Resin (20% w/v) (Bio-Rad Laboratories, Hercules, CA, USA) was employed to extract DNA from the resuspended pellet as formerly described [18]. Initial bacterial load was determined by analysing the processed samples by qPCR targeting the *mgp*B gene, as previously described [19].

Resuspended pellets in which *M. genitalium* was detected by qPCR were inoculated into a T-25 cell culture flask containing a 70% confluent Vero cell monolayer. The inoculation was performed in 3 ml of EMEM supplemented with 2% Ultroser G serum substitute and 1% l-glutamine. Thayer-Martin Supplement I (Sigma-Aldrich, St. Louis, MO, USA) at a 1/100 dilution was used to inhibit the growth of bacterial contaminants [17]. Thayer-Martin Supplement I contains 0.3 mg ml^−1^ vancomycin, 0.75 mg ml^−1^ colistin methane sulphonate, 0.5 mg ml^−1^ trimethoprim and 1,250 units nystatin. Also, cycloheximide solution (Sigma-Aldrich, St. Louis, MO, USA) at a 1/2,000 (0.0005%) dilution was used to prevent growth of yeast and fungi (this concentration slowed but did not prevent the growth of Vero cells). The inoculated flask was incubated at 37 °C in 5% CO_2_ for 8 weeks. The cells were observed daily for the first week and subsequently at 2- to 3-day intervals to identify cytopathic effects on the Vero cells or colour change and turbidity of the medium (evidence of contamination). In the event of cell detachment, fresh Vero cells (~2×10^4^ cells) were added. The medium was not changed during the first 2 weeks, after which it was changed on a weekly basis (without further trypsinization). At 1 week post-inoculation and subsequently at weekly intervals, 50 µl aliquots of the culture supernatant were collected and subjected to qPCR targeting the *mgpB* gene to determine *M. genitalium* loads [19]. Statistical analysis was performed using Stata 18 (Statacorp LLC, Texas, USA). Genome sequences were obtained using Oxford Nanopore sequencing as previously described [20] (manuscript in preparation).

## Results and discussion

### Sample characteristics

During the sampling period, a total of 127 clinical samples (from the same number of patients) that had tested positive using the Aptima^®^ TMA *Mycoplasma genitalium* assay were selected for laboratory analysis as described in Fig. 1. *M. genitalium* was detected by *mgpB* qPCR in 53.5% (68/127) of these samples, and they were inoculated in Vero cells. Notably, this discrepancy highlights a diagnostic gap between the Aptima^®^ diagnostic test and qPCR, which could be attributed to the higher analytical sensitivity of the Aptima^®^ TMA assay (which detects highly abundant rRNA molecules) [12,14]. Additionally, the samples tested by qPCR were frozen and stored prior to testing, which may have led to some sample degradation.

From the qPCR-positive group, 13 patients had not received any prior antibiotic treatment for the *M. genitalium* infection prior to the sample collection date. The remaining 55 patients had received antibiotics but had completed the course at least 14 days prior to sample collection and presented with symptoms on the day of collection. The inclusion criterion used in this study, which considered recent antibiotic treatment history, aimed to minimize the potential impact of recent antimicrobial exposure on bacterial viability and load. Such an impact could adversely affect the success of subsequent *M. genitalium* isolation. The specimens consisted of 40 neat urines, 24 high vaginal swabs and 4 anal swabs. Among the urine samples, 82.5% (33/40) were from males. For analysis purposes, *M. genitalium* loads were divided into five groups based on qPCR results (Table 1).

**Table 1.** Distribution of initial loads in samples detected by qPCR and inoculated in Vero cells

*Mg* loads in processed samples by qPCR (geq µl^−1^)	No. of qPCR-positive specimens (%)			*Totaln=68*
	Urine*n=40	High vaginal swab†*n*=24	Anal swab†*n*=4	
1–4	8 (20)	15 (62.5)	1 (25)	*24* (35.3)
5–19	20 (50)	7 (29.2)	3 (75)	*30* (44.1)
20–49	4 (10)	1 (4.2)	0	*5* (7.4)
50–99	1 (2.5)	1 (4.2)	0	*2* (2.9)
≥100	7 (17.5)	0	0	*7* (10.3)

*4 ml of sample was washed and resuspended in 0.5 ml of culture medium prior to PCR.

†1 ml of sample was washed and resuspended in 0.5 ml of culture medium prior to PCR.

### *M. genitalium* growth in Vero cells

An isolate of *M. genitalium* was obtained from 26.5% (18/68) of the qPCR-positive specimens following co-culture with Vero cells (Table 2). The isolation rate varied between sample types, with urine showing higher success (37.5%) than high vaginal swabs (12.5%; *P*=0.044 Fisher’s exact test). This may in part be explained by the fact that urine samples generally had a higher load and were less prone to contamination (see below). *M. genitalium* culture was unsuccessful in anal swabs, likely due to contamination.

**Table 2.** Summary of *M. genitalium* growth results by specimen type and concentration

		Cultures positive for growth (%)
*Specimen type*		
Anal swab		0/4 (0)
High vaginal swab		3/24 (12.5)
Urine		15/40 (37.5)
*M. genitalium* processed sample concentration (geq µl^−1^)*	Inoculum added per culture (geq)	
1–4	500–2,000	0/24 (0)
5–19	2,500–9,500	4/30 (13.3)
20–99	10,000–49,500	7/7 (100)
≥100	>50,000	7/7 (100)

*Concentration is indicated for processed sample. To convert to the number geq used in the inoculation of Vero cells, this number was multiplied by 500.

Previously, it was suggested that swab specimens were more likely to be successful for culture isolation than urine [16]. Our results contrast with this finding, noting that there were likely some differences in methodology (sample processing) and sample type (e.g. previous work used urethral or cervical swabs, but this study used high vaginal and anal swabs). The culture success rate for urine in the current study was lower than that reported by Doelman *et al*. (50%) [21] and Wood *et al*. (90%) [22]. The use of *Mycoplasma* transport media and a shorter time frame between sample collection and culture may have contributed to the higher culture success observed in those studies.

**Fig. 2.**
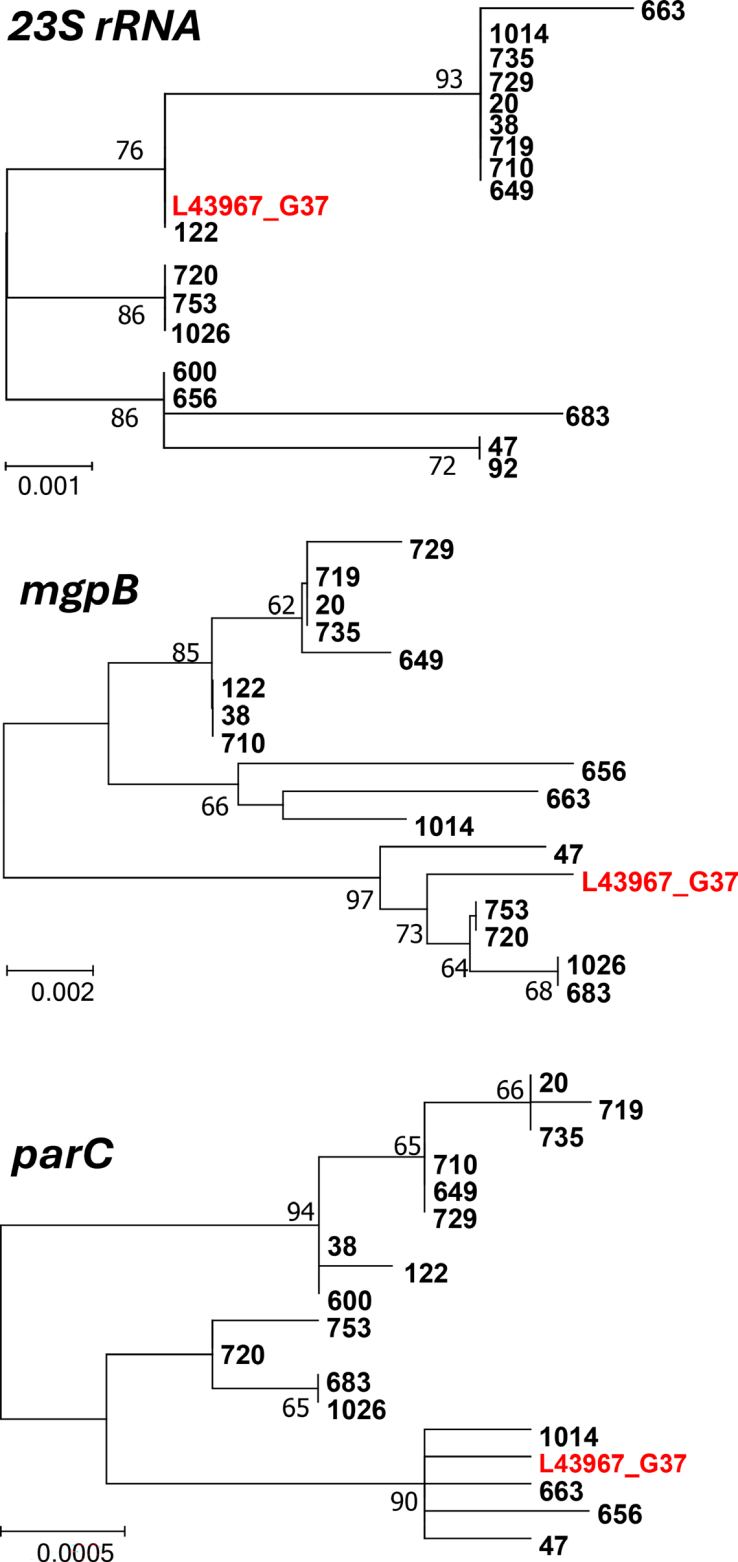
Maximum likelihood trees of *M. genitalium* isolates based on full-length 23S rRNA (2,734 bp), partial *mgpB* (320 bp) and full-length *parC* (2,346 bp) sequences. Bootstrap values are shown at the nodes. Reference strain G37 is shown in red. Culture isolates are shown in black. Gene sequence alignments can be found in File S1.

The success rate of *M. genitalium* isolation was significantly influenced by the initial bacterial load, which exhibited a clear positive correlation (*P* trend<0.0001) (Table 2). Considering a threshold of an inoculum of 10,000 genome equivalents (geq; corresponding to ≥20 geq μl^−1^ in 0.5 ml of processed sample), samples reaching this benchmark were significantly more likely to be cultured successfully (*P*<0.0001, Fisher’s exact test). Isolation was unsuccessful with low inoculum (<2,000 geq), partially successful (13.3%) with a moderate inoculum (2,500–9,500 geq) and highly successful (100%) in samples with a high inoculum (>10,000 geq). Phylogenetic analysis of a molecular typing gene (*mgpB*) and two resistance marker genes (23S rRNA, *parC*) showed that the samples were distributed across different branches, suggesting no cross-contamination of the cultured samples with a different *M. genitalium* strain (Fig. 2; File S1, available in the online Supplementary Material).

Our findings highlight the significant impact of the initial *M. genitalium* load on the likelihood of successful isolation, and this should be considered in the context of known information on load, as follows. We previously analysed loads in routine diagnostic samples [23]. In urine, the median female load was <1 geq μl^−1^, with only 9% of samples exceeding 20 geq μl^−1^. Additionally, 34% of samples surpassed the threshold of 10,000 geq in 4 ml (equivalent of 2.5 geq μl^−1^). The concentration in male urine was higher, with a median of ~7 geq μl^−1^ and 63% of samples reaching the above threshold. These results suggest that many routine clinical samples would be challenging to grow using the methods employed in this study. However, concentrating the samples would likely improve outcomes by increasing the detectable load and facilitating better growth.

Two studies have considered load prior to primary isolation of *M. genitalium*. Previously, Mondeja *et al*. [17] reported a high success rate for *M. genitalium* isolation using specimens with higher bacterial concentration (ranging from 67 to 5.8×10^4^ geq µl^−1^), although they note the likelihood of cross-contamination in their study. From 109 male urine samples, Hamasuna *et al*. [16] deemed 6 samples with suitable concentration to attempt culture (40 geq μl^−1^), of which 4 samples yielded successful isolates. Other bacterial isolation studies did not report bacterial concentrations [11,21, 22, 24]. The results of our study underscore the importance of optimizing sample collection methods to preserve high bacterial loads.

### Time to detection of bacterial growth

**Fig. 3.**
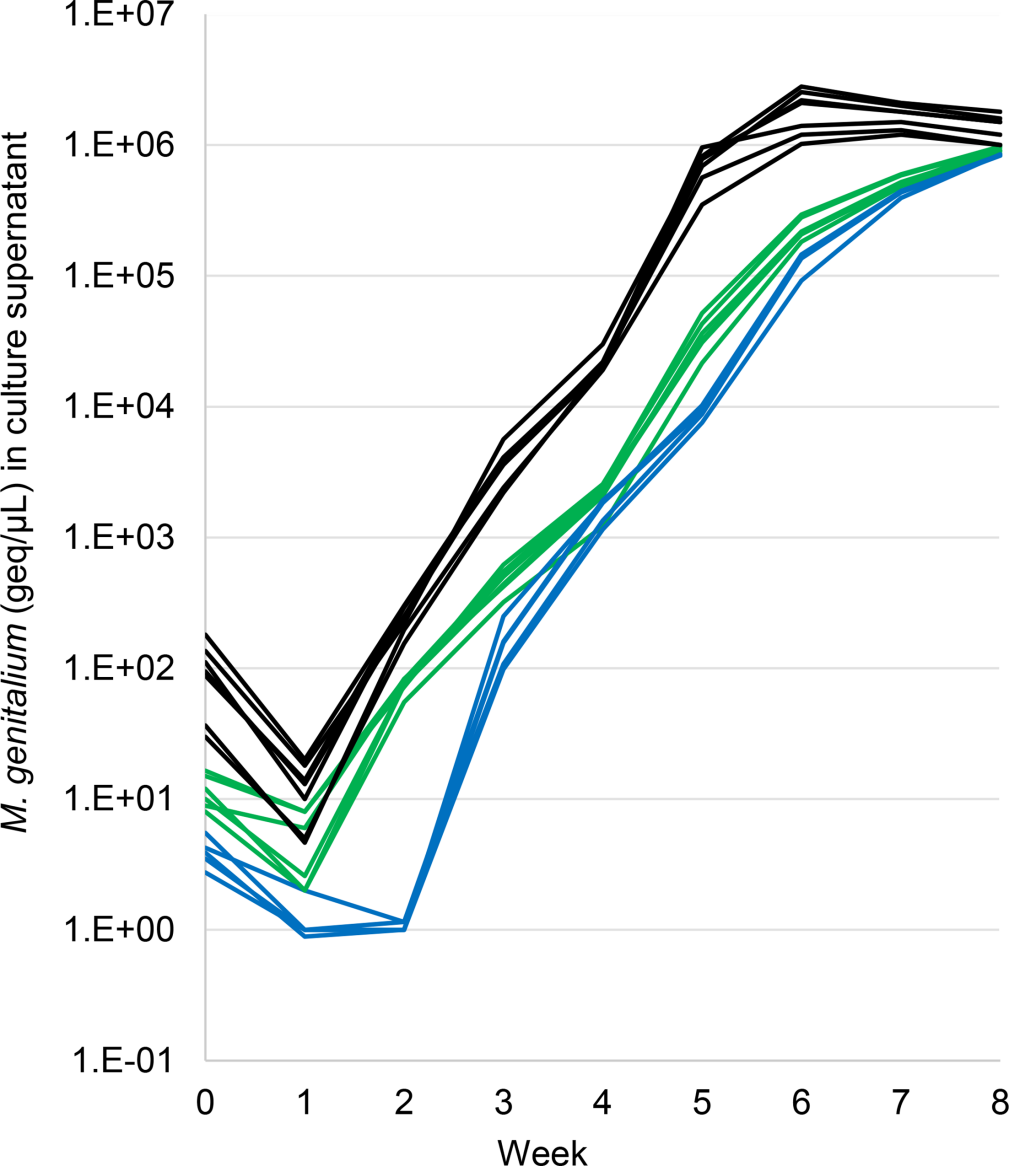
Growth curves of *M. genitalium* in Vero cell culture showing specimens with a low (<10,000 geq, blue), moderate (10,000–49,500 geq, green) and high (≥50,000 geq, black) inoculum. Samples were washed and then inoculated onto Vero cell monolayers, and the culture supernatant was sampled periodically by qPCR.

The bacterial concentration in samples over time was plotted to determine growth kinetics during isolation (Fig. 3). The * M. genitalium* concentration followed a distinct pattern over time, a decrease at 1 week after the inoculation followed by steady growth after 2 weeks post-infection. Samples with lower initial *M. genitalium* loads (below 100 geq μl^−1^ in the processed sample or total inoculum of <50,000 geq) achieved significant growth (arbitrarily defined as ≥1,000 geq μl^−1^) at 4 weeks post-infection in Vero cell cultures. In contrast, samples harbouring higher initial bacterial loads exhibited more rapid growth kinetics, reaching significant growth within 3 weeks post-infection (Fig. 3).

This observation indicates that the time required for *M. genitalium* to achieve significant growth in cell culture is directly influenced by the initial bacterial load of the inoculated sample. A 1–2-week lag phase was observed during bacterial isolation. A similar pattern is observed in the growth kinetics of *M. genitalium* after subculture of isolates previously established in cell culture systems [17,18]. After subculture, Hamasuna *et al*. [18] found the lag period differed for different isolates, with the majority displaying a 2-week lag phase, some slow growing (>2 weeks lag) and others fast (<1 week lag). This suggests that the genetic background of the bacteria may be a factor contributing to culture outcomes and potentially also to the success of primary isolation. Of note, the results presented in the current study relate to the primary isolation (processed patient specimen inoculated into Vero cells), and individual growth curves were not performed for each isolate following adaptation to culture.

Our findings also highlight the importance of considering the initial bacterial load when optimizing isolation protocols, as higher loads facilitate more rapid growth and successful recovery of viable isolates from clinical specimens. The decrease in * M. genitalium* load during the first week post-inoculation, followed by steady growth, suggests that this bacterium might require at least 1 week to establish an infection, potentially because of binding to and invading the host cells.

A significant challenge encountered during the isolation of *M. genitalium* from urogenital specimens was the growth of microbiota originating from these anatomical sites. In addition to competing for nutrients with *M. genitalium*, the microbiota poses risks of overgrowth, cytotoxicity and potential cross-contamination, which can ultimately lead to the failure of isolation attempts. Despite the use of antimicrobial reagents (Thayer-Martin Supplement I and cycloheximide solution), a substantial proportion of the inoculated swab specimens exhibited overgrowth by bacterial or fungal contaminants. The Thayer-Martin Supplement I was used because it contains vancomycin and colistin, which effectively inhibit the Gram-positive and Gram-negative bacteria commonly found in the urogenital tract [17]. Notably, 64.3% (18/28) of the swabs and 25.0% (10/40) of urine samples were overgrown by contaminant bacteria, while 5.9% (4/68) were overgrown by fungi. Higher prevalence of bacterial overgrowth was observed in anal swabs (100%, 4/4) compared to high vaginal swabs (58.3%, 14/24).

Despite bacterial overgrowth, *M. genitalium* was isolated in two high vaginal swabs (initial loads of 40 and 60 geq µl^−1^) and four urine samples (initial loads of 16, 17, 22 and 146 geq µl^−1^), demonstrating that isolation can occur even with relatively low initial loads. In these cases, contamination was resolved by filtering the culture through 0.45 µm centrifugal filters once it reached a high *M. genitalium* concentration (after ~8 weeks). These findings indicate that while bacterial overgrowth presents a significant challenge, isolation of *M. genitalium* can still be successful.

Interestingly, we observed a higher isolation rate from urine samples compared to swab samples, due in part to swabs having more contamination overgrowth. Previous work indicates that, in the absence of bacterial vaginosis, vaginal swab samples have similar bacterial load and composition to urine [25]. Besides the presence of urogenital microbiota, this discrepancy could be attributed to several factors, including *M. genitalium* tropism, potential loss of viability during swab collection and transport or the presence of inhibitory substances in swab samples.

Further investigations into sample-type-specific challenges, along with the use of more effective selective antibiotics targeting the urogenital microbiota, may help improve isolation rates from different specimen types. Additionally, future studies may explore the association between freezing time (the duration between sample collection and culture initiation) and isolation success.

### Limitations

Our study provides valuable insights into the factors that influence the isolation of *M. genitalium* from clinical specimens. However, it is important to acknowledge certain limitations. The sample size was relatively small, which may restrict the broader relevance and applicability of the results. It was not possible to identify what proportion of bacteria in the sample was viable, and this may be a key factor that determines the success of culture. The duration of sample storage while frozen may have impacted the viability of samples, but this was an uncontrolled variable that we were unable to incorporate into the analysis. Additionally, the study focused on the use of Vero cells for co-culture using one media formulation, and the findings may not be directly applicable to other cell lines, media or isolation methods. Therefore, future research may focus on alternative cell lines or co-culture systems and selective antibiotics that may offer more favourable growth conditions for *M. genitalium*.

## Conclusions

The initial bacterial load emerged as a critical determinant of isolation success, where an inoculum of ≥10,000 geq was most suitable for culture. This highlights the importance of optimizing sample collection and *M. genitalium* isolation procedures. Exploring different cell types or co-culture methods could potentially improve isolation rates, reduce incubation times and mitigate the challenges posed by contamination with commensal microbiota. The successful isolation of *M. genitalium* from clinical specimens enables downstream applications such as antimicrobial susceptibility testing, genomic characterization and the development of improved diagnostic assays.

## Supplementary material

10.1099/jmm.0.002040 Uncited Supplementary Material 1.

## Abbreviations

EMEM: Eagle’s minimum essential medium
gDNA: genomic DNA
geq: genome equivalents
qPCR: quantitative PCR
TMA: transcription-mediated amplification

## References

[1] Htaik K, Vodstrcil LA, Plummer EL, Sfameni AM, Machalek DA (2024). Systematic review and meta-analysis of the association between *Mycoplasma genitalium* and Pelvic Inflammatory Disease (PID). Clin Infect Dis.

[2] Scoullar MJL, Melepia P, Peach E, Fidelis R, Supsup H (2024). *Mycoplasma genitalium* in pregnancy, including specific co-infections, is associated with lower birthweight: a prospective cohort study. *Med*.

[3] Latimer RL, Vodstrcil LA, Plummer EL, Doyle M, Murray GL (2022). The clinical indications for testing women for *Mycoplasma genitalium*. Sex Transm Infect.

[4] Lis R, Rowhani-Rahbar A, Manhart LE (2015). *Mycoplasma genitalium* infection and female reproductive tract disease: a meta-analysis. Clin Infect Dis.

[5] Horner PJ, Martin DH (2017). *Mycoplasma genitalium* infection in men. J Infect Dis.

[6] Centers for Disease Control and Prevention (2019). Antibiotic Resistance Threats in the United States, 2019.

[7] Machalek DA, Tao Y, Shilling H, Jensen JS, Unemo M (2020). Prevalence of mutations associated with resistance to macrolides and fluoroquinolones in *Mycoplasma genitalium*: a systematic review and meta-analysis. Lancet Infect Dis.

[8] Chua T-P, Vodstrcil LA, Murray GL, Plummer EL, Jensen JS (2025). Evolving patterns of macrolide and fluoroquinolone resistance in *Mycoplasma genitalium*: an updated systematic review and meta-analysis. *Lancet Microbe*.

[9] Taylor-Robinson D, Jensen JS (2011). *Mycoplasma genitalium*: from Chrysalis to multicolored butterfly. Clin Microbiol Rev.

[10] Fraser CM, Gocayne JD, White O, Adams MD, Clayton RA (1995). The minimal gene complement of *Mycoplasma genitalium*. Science.

[11] Jensen JS, Hansen HT, Lind K (1996). Isolation of *Mycoplasma genitalium* strains from the male urethra. J Clin Microbiol.

[12] Kirkconnell B, Weinbaum B, Santos K, Le Nguyen T, Vinluan B (2019). Design and validation of transcription-mediated-amplification nucleic acid amplification tests for *Mycoplasma genitalium*. J Clin Microbiol.

[13] Unemo M, Salado-Rasmussen K, Hansen M, Olsen AO, Falk M (2018). Clinical and analytical evaluation of the new Aptima *Mycoplasma genitalium* assay, with data on *M. genitalium* prevalence and antimicrobial resistance in *M. genitalium* in Denmark, Norway and Sweden in 2016. Clin Microbiol Infect.

[14] Hamasuna R, Aono H, Kawaguchi K, Matsumoto M, Fujimoto N (2021). Sensitivity of a transcription-mediated amplification method (Aptima *Mycoplasma genitalium* assay) to detect *M. genitalium in vitro*. J Infect Chemother.

[15] Peh CR, Danielewski J, Chua TP, Bodiyabadu K, Machalek DA (2023). Quantitation of *Mycoplasma genitalium* using droplet digital PCR. Lett Appl Microbiol.

[16] Hamasuna R, Osada Y, Jensen JS (2007). Isolation of *Mycoplasma genitalium* from first-void urine specimens by coculture with Vero cells. J Clin Microbiol.

[17] Mondeja BA, Jensen JS, Rodríguez I, Morier LF, Kourí V (2013). Isolation of *Mycoplasma genitalium* from patients with urogenital infections: first report from the Latin-American region. New Microbes New Infect.

[18] Hamasuna R, Osada Y, Jensen JS (2005). Antibiotic susceptibility testing of *Mycoplasma genitalium* by TaqMan 5’ nuclease real-time PCR. Antimicrob Agents Chemother.

[19] Jensen JS, Björnelius E, Dohn B, Lidbrink P (2004). Use of TaqMan 5’ nuclease real-time PCR for quantitative detection of *Mycoplasma genitalium* DNA in males with and without urethritis who were attendees at a sexually transmitted disease clinic. J Clin Microbiol.

[20] Chua T-P, Danielewski J, Bradshaw CS, Machalek DA, Garland SM (2025). A novel azithromycin resistance mutation in *Mycoplasma genitalium* induced *in vitro*. J Antimicrob Chemother.

[21] Doelman TA, Adriaens N, Westerhuis BM, Bruisten SM, Vergunst CE (2025). Phenotypic antibiotic resistance of *Mycoplasma genitalium* and its variation between different macrolide resistance-associated mutations. J Antimicrob Chemother.

[22] Wood GE, Jensen NL, Astete S, Jensen JS, Kenny GE (2021). Azithromycin and doxycycline resistance profiles of U.S. *Mycoplasma genitalium* strains and their association with treatment outcomes. J Clin Microbiol.

[23] Murray GL, Danielewski J, Bodiyabadu K, Machalek DA, Bradshaw CS (2019). Analysis of infection loads in *Mycoplasma genitalium* clinical specimens by use of a commercial diagnostic test. J Clin Microbiol.

[24] Mondeja BA, Rodríguez NM, Barroto B, Blanco O, Jensen JS (2016). Antimicrobial susceptibility patterns of recent Cuban *Mycoplasma genitalium* isolates determined by a modified cell-culture-based method. PLoS One.

[25] Naicker D, Ramsuran V, Naicker M, Dessai F, Giandhari J (2021). Strong correlation between urine and vaginal swab samples for bacterial vaginosis. S Afr J Infect Dis.

