# Supplementary material for: Factors that contribute to the success of primary isolation of Mycoplasma genitalium from clinical samples
Source: J Med Microbiol. 2025 Jul 3;74(7):002040. doi: 10.1099/jmm.0.002040 (PMC12231096; doi:10.1099/jmm.0.002040)
Supplement: Uncited Supplementary Material 1. [file jmm-74-02040-s001.pdf]

***mgpB* alignment (partial sequence)**

```
47          ATGCACCAACCAAAGAAAAGACTGGCTAAGAAGTCTTGAGCCTTTCTAACCGCTGCACTT   60
L43967      ATGCACCAACCAAAGAAAAGACTGGCTAAGAAGTCTTGAGCCTTTCTAACCGCTGCACTT   60
683         ATGCACCAACCAAAGAAAAGACTGGCTAAGAAGTCTTGAGCCTTTCTAACCGCTGCACTT   60
720         ATGCACCAACCAAAGAAAAGACTGGCTAAGAAGTCTTGAGCCTTTCTAACCGCTGCACTT   60
753         ATGCACCAACCAAAGAAAAGACTGGCTAAGAAGTCTTGAGCCTTTCTAACCGCTGCACTT   60
1026        ATGCACCAACCAAAGAAAAGACTGGCTAAGAAGTCTTGAGCCTTTCTAACCGCTGCACTT   60
20          ATGCACCAACCAAAGAAAAGACTGGCTAAGAAGTCTTGAGCCTTTCTAACCGCTGCACTT   60
649         ATGCACCAACCAAAGAAAAGACTGGCTAAGAAGTCTTGAGCCTTTCTAACCGCTGCACTT   60
719         ATGCACCAACCAAAGAAAAGACTGGCTAAGAAGTCTTGAGCCTTTCTAACCGCTGCACTT   60
729         ATGCACCAACCAAAGAAAAGACTGGCTAAGAAGTCTTGAGCCTTTCTAACCGCTGCACTT   60
735         ATGCACCAACCAAAGAAAAGACTGGCTAAGAAGTCTTGAGCCTTTCTAACCGCTGCACTT   60
38          ATGCACCAACCAAAGAAAAGACTGGCTAAGAAGTCTTGAGCCTTTCTAACCGCTGCACTT   60
122         ATGCACCAACCAAAGAAAAGACTGGCTAAGAAGTCTTGAGCCTTTCTAACCGCTGCACTT   60
710         ATGCACCAACCAAAGAAAAGACTGGCTAAGAAGTCTTGAGCCTTTCTAACCGCTGCACTT   60
1014        ATGCACCAACCAAAGAAAAGACTGGCTAAGAAGTCTTGAGCCTTTCTAACCGCTGCACTT   60
656         ATGCACCAACCAAAGAAAAGACTGGCTAAGAAGTCTTGAGCCTTTCTAACCGCTGCACTT   60
663         ATGCACCAACCAAAGAAAAGACTGGCTAAGAAGTCTTGAGCCTTTCTAACCGCTGCACTT   60
            ************************************************************

47          ACCCTTGGGGTTGTAACAGGTGTAGGTGGTTATTTTCTCTTTAACCAAAATAAGCAACGT   120
L43967      ACCCTTGGGGTTATAACAGGTGTAGGTGGTTATTTTCTCTTTAACCAAAATAAGCAACGT   120
683         ACCCTTGGGGTTATAACAGGTGTAGGTGGTTATTTTCTCTTTAACCAAAATAAGCAACGT   120
720         ACCCTTGGGGTTATAACAGGTGTAGGTGGTTATTTTCTCTTTAACCAAAATAAGCAACGT   120
753         ACCCTTGGGGTTATAACAGGTGTAGGTGGTTATTTTCTCTTTAACCAAAATAAGCAACGT   120
1026        ACCCTTGGGGTTATAACAGGTGTAGGTGGTTATTTTCTCTTTAACCAAAATAAGCAACGT   120
20          ACCCTTGGGGTTATAACAGGTGTAGGTGGTTATTTTCTCTTTAACCAAAATAAGCAACGT   120
649         ACCCTTGGGGTTATAACAGGTGTAGGTGGTTATTTTCTCTTTAACCAAAATAAGCAACGT   120
719         ACCCTTGGGGTTATAACAGGTGTAGGTGGTTATTTTCTCTTTAACCAAAATAAGCAACGT   120
729         ACCCTTGGGGTTATAACAGGTGTAGGTGGTTATTTTCTCTTTAACCAAAATAAGCAACGT   120
735         ACCCTTGGGGTTATAACAGGTGTAGGTGGTTATTTTCTCTTTAACCAAAATAAGCAACGT   120
38          ACCCTTGGGGTTATAACAGGTGTAGGTGGTTATTTTCTCTTTAACCAAAATAAGCAACGT   120
122         ACCCTTGGGGTTATAACAGGTGTAGGTGGTTATTTTCTCTTTAACCAAAATAAGCAACGT   120
710         ACCCTTGGGGTTATAACAGGTGTAGGTGGTTATTTTCTCTTTAACCAAAATAAGCAACGT   120
1014        ACCCTTGGGGTTGTAACAGGTGTAGGTGGTTATTTTCTCTTTAACCAAAATAAGCAACGT   120
656         ACCCTTGGGGTTATAACAGGTGTAGGTGGTTATTTTCTCTTTAACCAAAATAAGCAACGT   120
663         ACCCTTGGGGTTATAACAGGTGTAGGTGGTTATTTTCTCTTTAACCAAAATAAGCAACGT   120
            ************ ***********************************************

47          AGTAGCGTGAGCAACTTTGCTTACCAACCCAAGCAGTTAAGTGTTAAACACCAACAAGCA   180
L43967      AGTAGCGTGAGCAACTTTGCTTACCAACCCAAGCAGTTAAGTGTTAAACACCAACAAGCA   180
683         AGTAGCGTGAGCAACTTTGCTTACCAACCCAAGCAGTTAAGTGTTAAACACCAACAAGCA   180
720         AGTAGCGTGAGCAACTTTGCTTACCAACCCAAGCAGTTAAGTGTTAAACACCAACAAGCA   180
753         AGTAGCGTGAGCAACTTTGCTTACCAACCCAAGCAGTTAAGTGTTAAACACCAACAAGCA   180
1026        AGTAGCGTGAGCAACTTTGCTTACCAACCCAAGCAGTTAAGTGTTAAACACCAACAAGCA   180
20          AGTAGCGTGAGCAACTTTGCTTACCAACCCAAGCAGTTAAGTGTTAAACACCAACAAGCA   180
649         AGTAGCGTGAGCAACTTTGCTTACCAACCCAAGCAGTTAAGTGTTAAACACCAACAAGCA   180
719         AGTAGCGTGAGCAACTTTGCTTACCAACCCAAGCAGTTAAGTGTTAAACACCAACAAGCA   180
729         AGTAGCGTGAGCAACTTTGCTTACCAACCCAAGCAGTTAAGTGTTAAACACCAACAAGCA   180
735         AGTAGCGTGAGCAACTTTGCTTACCAACCCAAGCAGTTAAGTGTTAAACACCAACAAGCA   180
38          AGTAGCGTGAGCAACTTTGCTTACCAACCCAAGCAGTTAAGTGTTAAACACCAACAAGCA   180
122         AGTAGCGTGAGCAACTTTGCTTACCAACCCAAGCAGTTAAGTGTTAAACACCAACAAGCA   180
710         AGTAGCGTGAGCAACTTTGCTTACCAACCCAAGCAGTTAAGTGTTAAACACCAACAAGCA   180
1014        AGTAGCGTGAGCAACTTTGCTTACCAACCCAAGCAGTTAAGTGTTAAACACCAACAAGCA   180
656         AGTAGCGTGAGCAACTTTGCTTACCAACCCAAGCAGTTAAGTGTTAAACACCAACAAGCA   180
663         AGTAGCGTGAGCAACTTTGCTTACCAACCCAAGCAGTTAAGTGTTAAACACCAACAAGCA   180
            ************************************************************

47          GTTGATGAAACCTTAACCCCTTGGACTTGAAACAATAACAACTTCTCTTCACTAAAGATT   240
L43967      GTTGATGAAACCTTAACCCCTTGGACTTGAAACAATAACAACTTCTCTTCACTAAAGATT   240
683         GTTGATGAAACCTTAACCCCTTGGACTTGAAACAATAACAACTTCTCTTCACTAAAGATT   240
720         GTTGATGAAACCTTAACCCCTTGGACTTGAAACAATAACAACTTCTCTTCACTAAAGATT   240
753         GTTGATGAAACCTTAACCCCTTGGACTTGAAACAATAACAACTTCTCTTCACTAAAGATT   240
1026        GTTGATGAAACCTTAACCCCTTGGACTTGAAACAATAACAACTTCTCTTCACTAAAGATT   240
20          GTTGATGAAACATTAACCCCTTGGACTTGAAACAATAACAACTTCTCTTCACTAAAGATT   240
649         GTTGATGAAACATTAACCCCTTGGACTTGAAACAATAACAACTTCTCTTCACTAAAGATT   240
719         GTTGATGAAACATTAACCCCTTGGACTTGAAACAATAACAACTTCTCTTCACTAAAGATT   240
729         GTTGATGAAACATTAACCCCTTGGACTTGAAACAATAACAACTTCTCTTCACTAAAGATT   240
735         GTTGATGAAACATTAACCCCTTGGACTTGAAACAATAACAACTTCTCTTCACTAAAGATT   240
38          GTTGATGAAACATTAACCCCTTGGACTTGAAACAATAACAACTTCTCTTCACTAAAGATT   240
122         GTTGATGAAACATTAACCCCTTGGACTTGAAACAATAACAACTTCTCTTCACTAAAGATT   240
710         GTTGATGAAACATTAACCCCTTGGACTTGAAACAATAACAACTTCTCTTCACTAAAGATT   240
1014        GTTGATGAAACATTAACCCCTTGGACTTGAAACAATAACAACTTCTCTTCACTGAAGATT   240
656         GTTGATGAAACCTTAACCCCTTGGACTTGAAACAATAACAACTTCTCTTCACTGAAGATT   240
663         GTTGATGAAACCTTAACCCCTTGGACTTGAAACAATAACAACTTCTCTTCACTGAAGATT   240
            *********** ***************************************** ******

47          ACTGGAGAGAACCCAGGATCATTTGGATTAGTAAGAAGCCAAAATGACAACTTAAATATT   300
L43967      ACTGGAGAGAACCCAGGATCATTTGGATTAGTAAGAAGCCAAAATGACAACTTAAATATT   300
683         ACTGGAGAGAACCCAGGATCATTTGGATTAGTAAGAAGCCAAAATGACAACTTAAATATT   300
720         ACTGGAGAGAACCCAGGATCATTTGGATTAGTAAGAAGCCAAAATGACAACTTAAATATT   300
753         ACTGGAGAGAACCCAGGATCATTTGGATTAGTAAGAAGCCAAAATGACAACTTAAATATT   300
```

```
1026        ACTGGAGAGAACCCAGGATCATTTGGATTAGTAAGAAGCCAAAATGACAACTTAAATATT   300
20          ACTGGAGAGAACCCAGGATCATTTGGACTAGTAAGAAGTCAAAATGAGAACTTAAACATC   300
649         ACTGGAGAGAACCCAGGATCATTTGGACTAGTAAGAAGTCAAAATGAGAACTTAAACATC   300
719         ACTGGAGAGAACCCAGGATCATTTGGACTAGTAAGAAGTCAAAATGAGAACTTAAACATC   300
729         ACTGGAGAGAACCCAGGATCATTTGGACTAGTAAGAAGTCAAAATGAGAACTTAAACATC   300
735         ACTGGAGAGAACCCAGGATCATTTGGACTAGTAAGAAGTCAAAATGAGAACTTAAACATC   300
38          ACTGGAGAGAACCCAGGATCATTTGGACTAGTAAGAAGCCAAAATGAGAACTTAAACATC   300
122         ACTGGAGAGAACCCAGGATCATTTGGACTAGTAAGAAGCCAAAATGAGAACTTAAACATC   300
710         ACTGGAGAGAACCCAGGATCATTTGGACTAGTAAGAAGCCAAAATGAGAACTTAAACATC   300
1014        ACTGGAGAGAACCCAGGATCATTTGGACTAGTAAGAAGCCAAAATGAGAACTTAAACATC   300
656         ACTGGAGAGAACCCAGGATCATTTGGACTAGTAAAAAGCCAAAATGAGAACTTAAACATC   300
663         ACTGGAGAGAACCCAGGATCATTTGGACTAGTAAGAAGCCAAAATGAGAACTTAAACATC   300
            *************************** ****** *** ******** ******** **

47          TCAAGTGTTACAAAGAATGG      320
L43967      TCAAGTGTTACAAAGAATTC      320
683         TCAAGTGTTACAAAGAATGT      320
720         TCAAGTGTTACAAAGAATGT      320
753         TCAAGTGTTACAAAGAATGT      320
1026        TCAAGTGTTACAAAGAATGT      320
20          GCAAGTGTTACAAAGAATGG      320
649         GCAAGTGTTACAAAGAATGG      320
719         GCAAGTGTTACAAAGAATGG      320
729         GCAAGTGTTACAAAGAATGG      320
735         GCAAGTGTTACAAAGAATGG      320
38          GCAAGTGTTACAAAGAATGG      320
122         GCAAGTGTTACAAAGAATGG      320
710         GCAAGTGTTACAAAGAATGG      320
1014        GCAAGTGTTACAAAGAATGG      320
656         GCAAGTGTTACAAAGAATGA      320
663         GCAAGTGTTACAAAGAATGG      320
             *****************
```

23S rRNA gene alignment

```
683         ACAATAAGTTACTAAGGGCTTATGGTGGATGCCTTGGCACTAAAAGGCGATGAAGGACGT   60
663         ACAATAAGTTACTAAGGGCTTATGGTGGATGCCTTGGCACTAAAAGGCGATGAAGGACGT   60
20          ACAATAAGTTACTAAGGGCTTATGGTGGATGCCTTGGCACTAAAAGGCGATGAAGGACGT   60
38          ACAATAAGTTACTAAGGGCTTATGGTGGATGCCTTGGCACTAAAAGGCGATGAAGGACGT   60
649         ACAATAAGTTACTAAGGGCTTATGGTGGATGCCTTGGCACTAAAAGGCGATGAAGGACGT   60
710         ACAATAAGTTACTAAGGGCTTATGGTGGATGCCTTGGCACTAAAAGGCGATGAAGGACGT   60
719         ACAATAAGTTACTAAGGGCTTATGGTGGATGCCTTGGCACTAAAAGGCGATGAAGGACGT   60
729         ACAATAAGTTACTAAGGGCTTATGGTGGATGCCTTGGCACTAAAAGGCGATGAAGGACGT   60
735_        ACAATAAGTTACTAAGGGCTTATGGTGGATGCCTTGGCACTAAAAGGCGATGAAGGACGT   60
1014        ACAATAAGTTACTAAGGGCTTATGGTGGATGCCTTGGCACTAAAAGGCGATGAAGGACGT   60
122         ACAATAAGTTACTAAGGGCTTATGGTGGATGCCTTGGCACTAAAAGGCGATGAAGGACGT   60
L43967      ACAATAAGTTACTAAGGGCTTATGGTGGATGCCTTGGCACTAAAAGGCGATGAAGGACGT   60
720         ACAATAAGTTACTAAGGGCTTATGGTGGATGCCTTGGCACTAAAAGGCGATGAAGGACGT   60
753         ACAATAAGTTACTAAGGGCTTATGGTGGATGCCTTGGCACTAAAAGGCGATGAAGGACGT   60
1026        ACAATAAGTTACTAAGGGCTTATGGTGGATGCCTTGGCACTAAAAGGCGATGAAGGACGT   60
47          ACAATAAGTTACTAAGGGCTTATGGTGGATGCCTTGGCACTAAAAGGCGATGAAGGACGT   60
92          ACAATAAGTTACTAAGGGCTTATGGTGGATGCCTTGGCACTAAAAGGCGATGAAGGACGT   60
600         ACAATAAGTTACTAAGGGCTTATGGTGGATGCCTTGGCACTAAAAGGCGATGAAGGACGT   60
656         ACAATAAGTTACTAAGGGCTTATGGTGGATGCCTTGGCACTAAAAGGCGATGAAGGACGT   60
            ************************************************************

683         GTTAACCTGCGATAAGCTTCGGGGAGGTGGTAAAAACCTGAGATCCGGAGGTGTCCGAAT   120
663         GTTAACCTGCGATAAGCTTCGGGGAGGTGGTAAAAACCTGAGATCCGGAGGTGTCCGAAT   120
20          GTTAACCTGCGATAAGCTTCGGGGAGGTGGTAAAAACCTGAGATCCGGAGGTGTCCGAAT   120
38          GTTAACCTGCGATAAGCTTCGGGGAGGTGGTAAAAACCTGAGATCCGGAGGTGTCCGAAT   120
649         GTTAACCTGCGATAAGCTTCGGGGAGGTGGTAAAAACCTGAGATCCGGAGGTGTCCGAAT   120
710         GTTAACCTGCGATAAGCTTCGGGGAGGTGGTAAAAACCTGAGATCCGGAGGTGTCCGAAT   120
719         GTTAACCTGCGATAAGCTTCGGGGAGGTGGTAAAAACCTGAGATCCGGAGGTGTCCGAAT   120
729         GTTAACCTGCGATAAGCTTCGGGGAGGTGGTAAAAACCTGAGATCCGGAGGTGTCCGAAT   120
735_        GTTAACCTGCGATAAGCTTCGGGGAGGTGGTAAAAACCTGAGATCCGGAGGTGTCCGAAT   120
1014        GTTAACCTGCGATAAGCTTCGGGGAGGTGGTAAAAACCTGAGATCCGGAGGTGTCCGAAT   120
122         GTTAACCTGCGATAAGCTTCGGGGAGGTGGTAAAAACCTGAGATCCGGAGGTGTCCGAAT   120
L43967      GTTAACCTGCGATAAGCTTCGGGGAGGTGGTAAAAACCTGAGATCCGGAGGTGTCCGAAT   120
720         GTTAACCTGCGATAAGCTTCGGGGAGGTGGTAAAAACCTGAGATCCGGAGGTGTCCGAAT   120
753         GTTAACCTGCGATAAGCTTCGGGGAGGTGGTAAAAACCTGAGATCCGGAGGTGTCCGAAT   120
1026        GTTAACCTGCGATAAGCTTCGGGGAGGTGGTAAAAACCTGAGATCCGGAGGTGTCCGAAT   120
47          GTTAACCTGCGATAAGCTTCGGGGAGGTGGTAAAAACCTGAGATCCGGAGGTGTCCGAAT   120
92          GTTAACCTGCGATAAGCTTCGGGGAGGTGGTAAAAACCTGAGATCCGGAGGTGTCCGAAT   120
600         GTTAACCTGCGATAAGCTTCGGGGAGGTGGTAAAAACCTGAGATCCGGAGGTGTCCGAAT   120
656         GTTAACCTGCGATAAGCTTCGGGGAGGTGGTAAAAACCTGAGATCCGGAGGTGTCCGAAT   120
            ************************************************************

683         GGAGCAATCTGGTAGCTTGCAAAAGTTACCATTAATTAATGAATTCATAGTTAATTAAAG   180
663         GGAGCAATCTGGTAGCTTGCAAAAGTTACCATTAATTAATGAATTCATAGTTAATTAAAG   180
20          GGAGCAATCTGGTAGCTTGCAAAAGTTACCATTAATTAATGAATTCATAGTTAATTAAAG   180
38          GGAGCAATCTGGTAGCTTGCAAAAGTTACCATTAATTAATGAATTCATAGTTAATTAAAG   180
649         GGAGCAATCTGGTAGCTTGCAAAAGTTACCATTAATTAATGAATTCATAGTTAATTAAAG   180
710         GGAGCAATCTGGTAGCTTGCAAAAGTTACCATTAATTAATGAATTCATAGTTAATTAAAG   180
719         GGAGCAATCTGGTAGCTTGCAAAAGTTACCATTAATTAATGAATTCATAGTTAATTAAAG   180
729         GGAGCAATCTGGTAGCTTGCAAAAGTTACCATTAATTAATGAATTCATAGTTAATTAAAG   180
735_        GGAGCAATCTGGTAGCTTGCAAAAGTTACCATTAATTAATGAATTCATAGTTAATTAAAG   180
1014        GGAGCAATCTGGTAGCTTGCAAAAGTTACCATTAATTAATGAATTCATAGTTAATTAAAG   180
122         GGAGCAATCTGGTAGCTTGCAAAAGTTACCATTAATTAATGAATTCATAGTTAATTAAAG   180
L43967      GGAGCAATCTGGTAGCTTGCAAAAGTTACCATTAATTAATGAATTCATAGTTAATTAAAG   180
720         GGAGCAATCTGGTAGCTTGCAAAAGTTACCATTAATTAATGAATTCATAGTTAATTAAAG   180
753         GGAGCAATCTGGTAGCTTGCAAAAGTTACCATTAATTAATGAATTCATAGTTAATTAAAG   180
1026        GGAGCAATCTGGTAGCTTGCAAAAGTTACCATTAATTAATGAATTCATAGTTAATTAAAG   180
47          GGAGCAATCTGGTAGCTTGCAAAAGTTACCATTAATTAATGAATTCATAGTTAATTAAAG   180
92          GGAGCAATCTGGTAGCTTGCAAAAGTTACCATTAATTAATGAATTCATAGTTAATTAAAG   180
600         GGAGCAATCTGGTAGCTTGCAAAAGTTACCATTAATTAATGAATTCATAGTTAATTAAAG   180
656         GGAGCAATCTGGTAGCTTGCAAAAGTTACCATTAATTAATGAATTCATAGTTAATTAAAG   180
            ************************************************************

683         CGATACGTGGTGAAGTGAAACATCTCAGTAACCACAGGAAAAGAAAACGAATGTGATTCC   240
663         CGATACGTGGTGAAGTGAAACATCTCAGTAACCACAGGAAAAGAAAACGAATGTGATTCC   240
20          CGATACGTGGTGAAGTGAAACATCTCAGTAACCACAGGAAAAGAAAACGAATGTGATTCC   240
38          CGATACGTGGTGAAGTGAAACATCTCAGTAACCACAGGAAAAGAAAACGAATGTGATTCC   240
649         CGATACGTGGTGAAGTGAAACATCTCAGTAACCACAGGAAAAGAAAACGAATGTGATTCC   240
710         CGATACGTGGTGAAGTGAAACATCTCAGTAACCACAGGAAAAGAAAACGAATGTGATTCC   240
719         CGATACGTGGTGAAGTGAAACATCTCAGTAACCACAGGAAAAGAAAACGAATGTGATTCC   240
729         CGATACGTGGTGAAGTGAAACATCTCAGTAACCACAGGAAAAGAAAACGAATGTGATTCC   240
735_        CGATACGTGGTGAAGTGAAACATCTCAGTAACCACAGGAAAAGAAAACGAATGTGATTCC   240
1014        CGATACGTGGTGAAGTGAAACATCTCAGTAACCACAGGAAAAGAAAACGAATGTGATTCC   240
122         CGATACGTGGTGAAGTGAAACATCTCAGTAACCACAGGAAAAGAAAACGAATGTGATTCC   240
L43967      CGATACGTGGTGAAGTGAAACATCTCAGTAACCACAGGAAAAGAAAACGAATGTGATTCC   240
720         CGATACGTGGTGAAGTGAAACATCTCAGTAACCACAGGAAAAGAAAACGAATGTGATTCC   240
753         CGATACGTGGTGAAGTGAAACATCTCAGTAACCACAGGAAAAGAAAACGAATGTGATTCC   240
1026        CGATACGTGGTGAAGTGAAACATCTCAGTAACCACAGGAAAAGAAAACGAATGTGATTCC   240
47          CGATACGTGGTGAAGTGAAACATCTCAGTAACCACAGGAAAAGAAAACGAATGTGATTCC   240
92          CGATACGTGGTGAAGTGAAACATCTCAGTAACCACAGGAAAAGAAAACGAATGTGATTCC   240
600         CGATACGTGGTGAAGTGAAACATCTCAGTAACCACAGGAAAAGAAAACGAATGTGATTCC   240
```

```
656        CGATACGTGGTGAAGTGAAACATCTCAGTAACCACAGGAAAAGAAAACGAATGTGATTCC   240
           ************************************************************

683        GTGTGTAGTGGCGAGCGAAAGCGGAACAGGCCAAACCTATCTGAGGATAGGGGTTGTAGG   300
663        GTGTGTAGTGGCGAGCGAAAGCGGAACAGGCCAAACCTATCTGAGGATAGGGGTTGTAGG   300
20         GTGTGTAGTGGCGAGCGAAAGCGGAACAGGCCAAACCTATCTGAGGATAGGGGTTGTAGG   300
38         GTGTGTAGTGGCGAGCGAAAGCGGAACAGGCCAAACCTATCTGAGGATAGGGGTTGTAGG   300
649        GTGTGTAGTGGCGAGCGAAAGCGGAACAGGCCAAACCTATCTGAGGATAGGGGTTGTAGG   300
710        GTGTGTAGTGGCGAGCGAAAGCGGAACAGGCCAAACCTATCTGAGGATAGGGGTTGTAGG   300
719        GTGTGTAGTGGCGAGCGAAAGCGGAACAGGCCAAACCTATCTGAGGATAGGGGTTGTAGG   300
729        GTGTGTAGTGGCGAGCGAAAGCGGAACAGGCCAAACCTATCTGAGGATAGGGGTTGTAGG   300
735_       GTGTGTAGTGGCGAGCGAAAGCGGAACAGGCCAAACCTATCTGAGGATAGGGGTTGTAGG   300
1014       GTGTGTAGTGGCGAGCGAAAGCGGAACAGGCCAAACCTATCTGAGGATAGGGGTTGTAGG   300
122        GTGTGTAGTGGCGAGCGAAAGCGGAACAGGCCAAACCTATCTGAGGATAGGGGTTGTAGG   300
L43967     GTGTGTAGTGGCGAGCGAAAGCGGAACAGGCCAAACCTATCTGAGGATAGGGGTTGTAGG   300
720        GTGTGTAGTGGCGAGCGAAAGCGGAACAGGCCAAACCTATCTGAGGATAGGGGTTGTAGG   300
753        GTGTGTAGTGGCGAGCGAAAGCGGAACAGGCCAAACCTATCTGAGGATAGGGGTTGTAGG   300
1026       GTGTGTAGTGGCGAGCGAAAGCGGAACAGGCCAAACCTATCTGAGGATAGGGGTTGTAGG   300
47         GTGTGTAGTGGCGAGCGAAAGCGGAACAGGCCAAACCTATCTGAGGATAGGGGTTGTAGG   300
92         GTGTGTAGTGGCGAGCGAAAGCGGAACAGGCCAAACCTATCTGAGGATAGGGGTTGTAGG   300
600        GTGTGTAGTGGCGAGCGAAAGCGGAACAGGCCAAACCTATCTGAGGATAGGGGTTGTAGG   300
656        GTGTGTAGTGGCGAGCGAAAGCGGAACAGGCCAAACCTATCTGAGGATAGGGGTTGTAGG   300
           ************************************************************

683        GCTTGCATTATGGAAGTTAAAAGGATAGAAGAAGCTGTTGGAAAGCAGCGCCAAAGAGGG   360
663        GCTTGCATTATGGAAGTTAAAAGGATAGAAGAAGCTGTTGGAAAGCAGCGCCAAAGAGGG   360
20         GCTTGCATTATGGAAGTTAAAAGGATAGAAGAAGCTGTTGGAAAGCAGCGCCAAAGAGGG   360
38         GCTTGCATTATGGAAGTTAAAAGGATAGAAGAAGCTGTTGGAAAGCAGCGCCAAAGAGGG   360
649        GCTTGCATTATGGAAGTTAAAAGGATAGAAGAAGCTGTTGGAAAGCAGCGCCAAAGAGGG   360
710        GCTTGCATTATGGAAGTTAAAAGGATAGAAGAAGCTGTTGGAAAGCAGCGCCAAAGAGGG   360
719        GCTTGCATTATGGAAGTTAAAAGGATAGAAGAAGCTGTTGGAAAGCAGCGCCAAAGAGGG   360
729        GCTTGCATTATGGAAGTTAAAAGGATAGAAGAAGCTGTTGGAAAGCAGCGCCAAAGAGGG   360
735_       GCTTGCATTATGGAAGTTAAAAGGATAGAAGAAGCTGTTGGAAAGCAGCGCCAAAGAGGG   360
1014       GCTTGCATTATGGAAGTTAAAAGGATAGAAGAAGCTGTTGGAAAGCAGCGCCAAAGAGGG   360
122        GCTTGCATTATGGAAGTTAAAAGGATAGAAGAAGCTGTTGGAAAGCAGCGCCAAAGAGGG   360
L43967     GCTTGCATTATGGAAGTTAAAAGGATAGAAGAAGCTGTTGGAAAGCAGCGCCAAAGAGGG   360
720        GCTTGCATTATGGAAGTTAAAAGGATAGAAGAAGCTGTTGGAAAGCAGCGCCAAAGAGGG   360
753        GCTTGCATTATGGAAGTTAAAAGGATAGAAGAAGCTGTTGGAAAGCAGCGCCAAAGAGGG   360
1026       GCTTGCATTATGGAAGTTAAAAGGATAGAAGAAGCTGTTGGAAAGCAGCGCCAAAGAGGG   360
47         GCTTGCATTATGGAAGTTAAAAGGATAGAAGAAGCTGTTGGAAAGCAGCGCCAAAGAGGG   360
92         GCTTGCATTATGGAAGTTAAAAGGATAGAAGAAGCTGTTGGAAAGCAGCGCCAAAGAGGG   360
600        GCTTGCATTATGGAAGTTAAAAGGATAGAAGAAGCTGTTGGAAAGCAGCGCCAAAGAGGG   360
656        GCTTGCATTATGGAAGTTAAAAGGATAGAAGAAGCTGTTGGAAAGCAGCGCCAAAGAGGG   360
           ************************************************************

683        TGATAGCCCCGTATTTGAAATCTTTTTAATACCTAGCAAGAAACCTGAGTAGCTCGAAAA   420
663        TGATAGCCCCGTATTTGAAATCTTTTTAATACCTAGCAAGAAACCTGAGTAGCTCGAAAA   420
20         TGATAGCCCCGTATTTGAAATCTTTTTAATACCTAGCAAGAAACCTGAGTAGCTCGAAAA   420
38         TGATAGCCCCGTATTTGAAATCTTTTTAATACCTAGCAAGAAACCTGAGTAGCTCGAAAA   420
649        TGATAGCCCCGTATTTGAAATCTTTTTAATACCTAGCAAGAAACCTGAGTAGCTCGAAAA   420
710        TGATAGCCCCGTATTTGAAATCTTTTTAATACCTAGCAAGAAACCTGAGTAGCTCGAAAA   420
719        TGATAGCCCCGTATTTGAAATCTTTTTAATACCTAGCAAGAAACCTGAGTAGCTCGAAAA   420
729        TGATAGCCCCGTATTTGAAATCTTTTTAATACCTAGCAAGAAACCTGAGTAGCTCGAAAA   420
735_       TGATAGCCCCGTATTTGAAATCTTTTTAATACCTAGCAAGAAACCTGAGTAGCTCGAAAA   420
1014       TGATAGCCCCGTATTTGAAATCTTTTTAATACCTAGCAAGAAACCTGAGTAGCTCGAAAA   420
122        TGATAGCCCCGTATTTGAAATCTTTTTAATACCTAGCAAGAAACCTGAGTAGCTCGAAAA   420
L43967     TGATAGCCCCGTATTTGAAATCTTTTTAATACCTAGCAAGAAACCTGAGTAGCTCGAAAA   420
720        TGATAGCCCCGTATTTGAAATCTTTTTAATACCTAGCAAGAAACCTGAGTAGCTCGAAAA   420
753        TGATAGCCCCGTATTTGAAATCTTTTTAATACCTAGCAAGAAACCTGAGTAGCTCGAAAA   420
1026       TGATAGCCCCGTATTTGAAATCTTTTTAATACCTAGCAAGAAACCTGAGTAGCTCGAAAA   420
47         TGATAGCCCCGTATTTGAAATCTTTTTAATACCTAGCAAGAAACCTGAGTAGCTCGAAAA   420
92         TGATAGCCCCGTATTTGAAATCTTTTTAATACCTAGCAAGAAACCTGAGTAGCTCGAAAA   420
600        TGATAGCCCCGTATTTGAAATCTTTTTAATACCTAGCAAGAAACCTGAGTAGCTCGAAAA   420
656        TGATAGCCCCGTATTTGAAATCTTTTTAATACCTAGCAAGAAACCTGAGTAGCTCGAAAA   420
           ************************************************************

683        ACGTTATTTTGAGTGAATCTGCCCAGACCATTGGGTAAGCCTAAATACTAATTAGTGACC   480
663        ACGTTATTTTGAGTGAATCTGCCCAGACCATTGGGTAAGCCTAAATACTAATTAGTGACC   480
20         ACGTTATTTTGAGTGAATCTGCCCAGACCATTGGGTAAGCCTAAATACTAATTAGTGACC   480
38         ACGTTATTTTGAGTGAATCTGCCCAGACCATTGGGTAAGCCTAAATACTAATTAGTGACC   480
649        ACGTTATTTTGAGTGAATCTGCCCAGACCATTGGGTAAGCCTAAATACTAATTAGTGACC   480
710        ACGTTATTTTGAGTGAATCTGCCCAGACCATTGGGTAAGCCTAAATACTAATTAGTGACC   480
719        ACGTTATTTTGAGTGAATCTGCCCAGACCATTGGGTAAGCCTAAATACTAATTAGTGACC   480
729        ACGTTATTTTGAGTGAATCTGCCCAGACCATTGGGTAAGCCTAAATACTAATTAGTGACC   480
735_       ACGTTATTTTGAGTGAATCTGCCCAGACCATTGGGTAAGCCTAAATACTAATTAGTGACC   480
1014       ACGTTATTTTGAGTGAATCTGCCCAGACCATTGGGTAAGCCTAAATACTAATTAGTGACC   480
122        ACGTTATTTTGAGTGAATCTGCCCAGACCATTGGGTAAGCCTAAATACTAATTAGTGACC   480
L43967     ACGTTATTTTGAGTGAATCTGCCCAGACCATTGGGTAAGCCTAAATACTAATTAGTGACC   480
720        ACGTTATTTTGAGTGAATCTGCCCAGACCATTGGGTAAGCCTAAATACTAATTAGTGACC   480
753        ACGTTATTTTGAGTGAATCTGCCCAGACCATTGGGTAAGCCTAAATACTAATTAGTGACC   480
1026       ACGTTATTTTGAGTGAATCTGCCCAGACCATTGGGTAAGCCTAAATACTAATTAGTGACC   480
47         ACGTTATTTTGAGTGAATCTGCCCAGACCATTGGGTAAGCCTAAATACTAATTAGTGACC   480
92         ACGTTATTTTGAGTGAATCTGCCCAGACCATTGGGTAAGCCTAAATACTAATTAGTGACC   480
```

```
600         ACGTTATTTTGAGTGAATCTGCCCAGACCATTGGGTAAGCCTAAATACTAATTAGTGACC   480
656         ACGTTATTTTGAGTGAATCTGCCCAGACCATTGGGTAAGCCTAAATACTAATTAGTGACC   480
            ************************************************************

683         GATAGCGAAACAGTACCGTGAGGGAAAGGTGAAAAGAACCCAGAGATGGGAGTGAAATAG   540
663         GATAGCGAAACAGTACCGTGAGGGAAAGGTGAAAAGAACCCAGAGATGGGAGTGAAATAG   540
20          GATAGCGAAACAGTACCGTGAGGGAAAGGTGAAAAGAACCCAGAGATGGGAGTGAAATAG   540
38          GATAGCGAAACAGTACCGTGAGGGAAAGGTGAAAAGAACCCAGAGATGGGAGTGAAATAG   540
649         GATAGCGAAACAGTACCGTGAGGGAAAGGTGAAAAGAACCCAGAGATGGGAGTGAAATAG   540
710         GATAGCGAAACAGTACCGTGAGGGAAAGGTGAAAAGAACCCAGAGATGGGAGTGAAATAG   540
719         GATAGCGAAACAGTACCGTGAGGGAAAGGTGAAAAGAACCCAGAGATGGGAGTGAAATAG   540
729         GATAGCGAAACAGTACCGTGAGGGAAAGGTGAAAAGAACCCAGAGATGGGAGTGAAATAG   540
735_        GATAGCGAAACAGTACCGTGAGGGAAAGGTGAAAAGAACCCAGAGATGGGAGTGAAATAG   540
1014        GATAGCGAAACAGTACCGTGAGGGAAAGGTGAAAAGAACCCAGAGATGGGAGTGAAATAG   540
122         GATAGCGAAACAGTACCGTGAGGGAAAGGTGAAAAGAACCCAGAGATGGGAGTGAAATAG   540
L43967      GATAGCGAAACAGTACCGTGAGGGAAAGGTGAAAAGAACCCAGAGATGGGAGTGAAATAG   540
720         GATAGCGAAACAGTACCGTGAGGGAAAGGTGAAAAGAACCCAGAGATGGGAGTGAAATAG   540
753         GATAGCGAAACAGTACCGTGAGGGAAAGGTGAAAAGAACCCAGAGATGGGAGTGAAATAG   540
1026        GATAGCGAAACAGTACCGTGAGGGAAAGGTGAAAAGAACCCAGAGATGGGAGTGAAATAG   540
47          GATAGCGAAACAGTACCGTGAGGGAAAGGTGAAAAGAACCCAGAGATGGGAGTGAAATAG   540
92          GATAGCGAAACAGTACCGTGAGGGAAAGGTGAAAAGAACCCAGAGATGGGAGTGAAATAG   540
600         GATAGCGAAACAGTACCGTGAGGGAAAGGTGAAAAGAACCCAGAGATGGGAGTGAAATAG   540
656         GATAGCGAAACAGTACCGTGAGGGAAAGGTGAAAAGAACCCAGAGATGGGAGTGAAATAG   540
            ************************************************************

683         ATTCTGAAACCATATGCCTACAACGTGTCAGAGCACATTAATGTGTGATGGCGTGCGTTT   600
663         ATTCTGAAACCATATGCCTACAACGTGTCAGAGCACATTAATGTGTGATGGCGTGCGTTT   600
20          ATTCTGAAACCATATGCCTACAACGTGTCAGAGCACATTAATGTGTGATGGCGTGCGTTT   600
38          ATTCTGAAACCATATGCCTACAACGTGTCAGAGCACATTAATGTGTGATGGCGTGCGTTT   600
649         ATTCTGAAACCATATGCCTACAACGTGTCAGAGCACATTAATGTGTGATGGCGTGCGTTT   600
710         ATTCTGAAACCATATGCCTACAACGTGTCAGAGCACATTAATGTGTGATGGCGTGCGTTT   600
719         ATTCTGAAACCATATGCCTACAACGTGTCAGAGCACATTAATGTGTGATGGCGTGCGTTT   600
729         ATTCTGAAACCATATGCCTACAACGTGTCAGAGCACATTAATGTGTGATGGCGTGCGTTT   600
735_        ATTCTGAAACCATATGCCTACAACGTGTCAGAGCACATTAATGTGTGATGGCGTGCGTTT   600
1014        ATTCTGAAACCATATGCCTACAACGTGTCAGAGCACATTAATGTGTGATGGCGTGCGTTT   600
122         ATTCTGAAACCATATGCCTACAACGTGTCAGAGCACATTAATGTGTGATGGCGTGCGTTT   600
L43967      ATTCTGAAACCATATGCCTACAACGTGTCAGAGCACATTAATGTGTGATGGCGTGCGTTT   600
720         ATTCTGAAACCATATGCCTACAACGTGTCAGAGCACATTAATGTGTGATGGCGTGCGTTT   600
753         ATTCTGAAACCATATGCCTACAACGTGTCAGAGCACATTAATGTGTGATGGCGTGCGTTT   600
1026        ATTCTGAAACCATATGCCTACAACGTGTCAGAGCACATTAATGTGTGATGGCGTGCGTTT   600
47          ATTCTGAAACCATATGCCTACAACGTGTCAGAGCACATTAATGTGTGATGGCGTGCGTTT   600
92          ATTCTGAAACCATATGCCTACAACGTGTCAGAGCACATTAATGTGTGATGGCGTGCGTTT   600
600         ATTCTGAAACCATATGCCTACAACGTGTCAGAGCACATTAATGTGTGATGGCGTGCGTTT   600
656         ATTCTGAAACCATATGCCTACAACGTGTCAGAGCACATTAATGTGTGATGGCGTGCGTTT   600
            ************************************************************

683         TGAAGTATGAGCCGGCGAGTTATGATAGCAAGCGTTAGTTAACCAGGAGATGGGGAGCTG   660
663         TGAAGTATGAGCCGGCGAGTTATGATAGCAAGCGTTAGTTAACCAGGAGATGGGGAGCTG   660
20          TGAAGTATGAGCCGGCGAGTTATGATAGCAAGCGTTAGTTAACCAGGAGATGGGGAGCTG   660
38          TGAAGTATGAGCCGGCGAGTTATGATAGCAAGCGTTAGTTAACCAGGAGATGGGGAGCTG   660
649         TGAAGTATGAGCCGGCGAGTTATGATAGCAAGCGTTAGTTAACCAGGAGATGGGGAGCTG   660
710         TGAAGTATGAGCCGGCGAGTTATGATAGCAAGCGTTAGTTAACCAGGAGATGGGGAGCTG   660
719         TGAAGTATGAGCCGGCGAGTTATGATAGCAAGCGTTAGTTAACCAGGAGATGGGGAGCTG   660
729         TGAAGTATGAGCCGGCGAGTTATGATAGCAAGCGTTAGTTAACCAGGAGATGGGGAGCTG   660
735_        TGAAGTATGAGCCGGCGAGTTATGATAGCAAGCGTTAGTTAACCAGGAGATGGGGAGCTG   660
1014        TGAAGTATGAGCCGGCGAGTTATGATAGCAAGCGTTAGTTAACCAGGAGATGGGGAGCTG   660
122         TGAAGTATGAGCCGGCGAGTTATGATAGCAAGCGTTAGTTAACCAGGAGATGGGGAGCTG   660
L43967      TGAAGTATGAGCCGGCGAGTTATGATAGCAAGCGTTAGTTAACCAGGAGATGGGGAGCTG   660
720         TGAAGTATGAGCCGGCGAGTTATGATAGCAAGCGTTAGTTAACCAGGAGATGGGGAGCTG   660
753         TGAAGTATGAGCCGGCGAGTTATGATAGCAAGCGTTAGTTAACCAGGAGATGGGGAGCTG   660
1026        TGAAGTATGAGCCGGCGAGTTATGATAGCAAGCGTTAGTTAACCAGGAGATGGGGAGCTG   660
47          TGAAGTATGAGCCGGCGAGTTATGATAGCAAGCGTTAGTTAACCAGGAGATGGGGAGCTG   660
92          TGAAGTATGAGCCGGCGAGTTATGATAGCAAGCGTTAGTTAACCAGGAGATGGGGAGCTG   660
600         TGAAGTATGAGCCGGCGAGTTATGATAGCAAGCGTTAGTTAACCAGGAGATGGGGAGCTG   660
656         TGAAGTATGAGCCGGCGAGTTATGATAGCAAGCGTTAGTTAACCAGGAGATGGGGAGCTG   660
            ************************************************************

683         TAGCGAAAGCGAGTTTTAAGAGAGCGTTTGTTTGTTATCATAGACCCGAAACGGGTTGAG   720
663         TAGCGAAAGCGAGTTTTAAGAGAGCGTTTGTTTGTTATCATAGACCCGAAACGGGTTGAG   720
20          TAGCGAAAGCGAGTTTTAAGAGAGCGTTTGTTTGTTATCATAGACCCGAAACGGGTTGAG   720
38          TAGCGAAAGCGAGTTTTAAGAGAGCGTTTGTTTGTTATCATAGACCCGAAACGGGTTGAG   720
649         TAGCGAAAGCGAGTTTTAAGAGAGCGTTTGTTTGTTATCATAGACCCGAAACGGGTTGAG   720
710         TAGCGAAAGCGAGTTTTAAGAGAGCGTTTGTTTGTTATCATAGACCCGAAACGGGTTGAG   720
719         TAGCGAAAGCGAGTTTTAAGAGAGCGTTTGTTTGTTATCATAGACCCGAAACGGGTTGAG   720
729         TAGCGAAAGCGAGTTTTAAGAGAGCGTTTGTTTGTTATCATAGACCCGAAACGGGTTGAG   720
735_        TAGCGAAAGCGAGTTTTAAGAGAGCGTTTGTTTGTTATCATAGACCCGAAACGGGTTGAG   720
1014        TAGCGAAAGCGAGTTTTAAGAGAGCGTTTGTTTGTTATCATAGACCCGAAACGGGTTGAG   720
122         TAGCGAAAGCGAGTTTTAAGAGAGCGTTTGTTTGTTATCATAGACCCGAAACGGGTTGAG   720
L43967      TAGCGAAAGCGAGTTTTAAGAGAGCGTTTGTTTGTTATCATAGACCCGAAACGGGTTGAG   720
720         TAGCGAAAGCGAGTTTTAAGAGAGCGTTTGTTTGTTATCATAGACCCGAAACGGGTTGAG   720
753         TAGCGAAAGCGAGTTTTAAGAGAGCGTTTGTTTGTTATCATAGACCCGAAACGGGTTGAG   720
1026        TAGCGAAAGCGAGTTTTAAGAGAGCGTTTGTTTGTTATCATAGACCCGAAACGGGTTGAG   720
47          TAGCGAAAGCGAGTTTTAAGAGAGCGTTTGTTTGTTATCATAGACCCGAAACGGGTTGAG   720
```

```
92          TAGCGAAAGCGAGTTTTAAGAGAGCGTTTGTTTGTTATCATAGACCCGAAACGGGTTGAG   720
600         TAGCGAAAGCGAGTTTTAAGAGAGCGTTTGTTTGTTATCATAGACCCGAAACGGGTTGAG   720
656         TAGCGAAAGCGAGTTTTAAGAGAGCGTTTGTTTGTTATCATAGACCCGAAACGGGTTGAG   720
            ************************************************************

683         CTAGTCATGAGCAGGTTGAAGGTTGAGTAACATTAACTGGAGGACCGAACCGACTCTCGT   780
663         CTAGTCATGAGCAGGTTGAAGGTTGAGTAACATTAACTGGAGGACCGAACCGACTCTCGT   780
20          CTAGTCATGAGCAGGTTGAAGGTTGAGTAACATTAACTGGAGGACCGAACCGACTCTCGT   780
38          CTAGTCATGAGCAGGTTGAAGGTTGAGTAACATTAACTGGAGGACCGAACCGACTCTCGT   780
649         CTAGTCATGAGCAGGTTGAAGGTTGAGTAACATTAACTGGAGGACCGAACCGACTCTCGT   780
710         CTAGTCATGAGCAGGTTGAAGGTTGAGTAACATTAACTGGAGGACCGAACCGACTCTCGT   780
719         CTAGTCATGAGCAGGTTGAAGGTTGAGTAACATTAACTGGAGGACCGAACCGACTCTCGT   780
729         CTAGTCATGAGCAGGTTGAAGGTTGAGTAACATTAACTGGAGGACCGAACCGACTCTCGT   780
735_        CTAGTCATGAGCAGGTTGAAGGTTGAGTAACATTAACTGGAGGACCGAACCGACTCTCGT   780
1014        CTAGTCATGAGCAGGTTGAAGGTTGAGTAACATTAACTGGAGGACCGAACCGACTCTCGT   780
122         CTAGTCATGAGCAGGTTGAAGGTTGAGTAACATTAACTGGAGGACCGAACCGACTCTCGT   780
L43967      CTAGTCATGAGCAGGTTGAAGGTTGAGTAACATTAACTGGAGGACCGAACCGACTCTCGT   780
720         CTAGTCATGAGCAGGTTGAAGGTTGAGTAACATTAACTGGAGGACCGAACCGACTCTCGT   780
753         CTAGTCATGAGCAGGTTGAAGGTTGAGTAACATTAACTGGAGGACCGAACCGACTCTCGT   780
1026        CTAGTCATGAGCAGGTTGAAGGTTGAGTAACATTAACTGGAGGACCGAACCGACTCTCGT   780
47          CTAGTCATGAGCAGGTTGAAGGTTGAGTAACATTAACTGGAGGACCGAACCGACTCTCGT   780
92          CTAGTCATGAGCAGGTTGAAGGTTGAGTAACATTAACTGGAGGACCGAACCGACTCTCGT   780
600         CTAGTCATGAGCAGGTTGAAGGTTGAGTAACATTAACTGGAGGACCGAACCGACTCTCGT   780
656         CTAGTCATGAGCAGGTTGAAGGTTGAGTAACATTAACTGGAGGACCGAACCGACTCTCGT   780
            ************************************************************

683         TGAAACGATAGCGGATGACTTGTGATTAGGGGTGAAATTCCAATCGAAATCCGTGATAGC   840
663         TGAAACGATAGCGGATGACTTGTGATTAGGGGTGAAATTCCAATCGAAATCCGTGATAGC   840
20          TGAAACGATAGCGGATGACTTGTGATTAGGGGTGAAATTCCAATCGAAATCCGTGATAGC   840
38          TGAAACGATAGCGGATGACTTGTGATTAGGGGTGAAATTCCAATCGAAATCCGTGATAGC   840
649         TGAAACGATAGCGGATGACTTGTGATTAGGGGTGAAATTCCAATCGAAATCCGTGATAGC   840
710         TGAAACGATAGCGGATGACTTGTGATTAGGGGTGAAATTCCAATCGAAATCCGTGATAGC   840
719         TGAAACGATAGCGGATGACTTGTGATTAGGGGTGAAATTCCAATCGAAATCCGTGATAGC   840
729         TGAAACGATAGCGGATGACTTGTGATTAGGGGTGAAATTCCAATCGAAATCCGTGATAGC   840
735_        TGAAACGATAGCGGATGACTTGTGATTAGGGGTGAAATTCCAATCGAAATCCGTGATAGC   840
1014        TGAAACGATAGCGGATGACTTGTGATTAGGGGTGAAATTCCAATCGAAATCCGTGATAGC   840
122         TGAAACGATAGCGGATGACTTGTGATTAGGGGTGAAATTCCAATCGAAATCCGTGATAGC   840
L43967      TGAAACGATAGCGGATGACTTGTGATTAGGGGTGAAATTCCAATCGAAATCCGTGATAGC   840
720         TGAAACGATAGCGGATGACTTGTGATTAGGGGTGAAATTCCAATCGAAATCCGTGATAGC   840
753         TGAAACGATAGCGGATGACTTGTGATTAGGGGTGAAATTCCAATCGAAATCCGTGATAGC   840
1026        TGAAACGATAGCGGATGACTTGTGATTAGGGGTGAAATTCCAATCGAAATCCGTGATAGC   840
47          TGAAACGATAGCGGATGACTTGTGATTAGGGGTGAAATTCCAATCGAAATCCGTGATAGC   840
92          TGAAACGATAGCGGATGACTTGTGATTAGGGGTGAAATTCCAATCGAAATCCGTGATAGC   840
600         TGAAACGATAGCGGATGACTTGTGATTAGGGGTGAAATTCCAATCGAAATCCGTGATAGC   840
656         TGAAACGATAGCGGATGACTTGTGATTAGGGGTGAAATTCCAATCGAAATCCGTGATAGC   840
            ************************************************************

683         TGGTTCTCGTCGAAATAGCTTTAAGGCTAGCGTAAGATCACAAATAAGTGGAGGTAAAGC   900
663         TGGTTCTCGTCGAAATAGCTTTAAGGCTAGCGTAAGATCACAAATAAGTGGAGGTAAAGC   900
20          TGGTTCTCGTCGAAATAGCTTTAAGGCTAGCGTAAGATCACAAATAAGTGGAGGTAAAGC   900
38          TGGTTCTCGTCGAAATAGCTTTAAGGCTAGCGTAAGATCACAAATAAGTGGAGGTAAAGC   900
649         TGGTTCTCGTCGAAATAGCTTTAAGGCTAGCGTAAGATCACAAATAAGTGGAGGTAAAGC   900
710         TGGTTCTCGTCGAAATAGCTTTAAGGCTAGCGTAAGATCACAAATAAGTGGAGGTAAAGC   900
719         TGGTTCTCGTCGAAATAGCTTTAAGGCTAGCGTAAGATCACAAATAAGTGGAGGTAAAGC   900
729         TGGTTCTCGTCGAAATAGCTTTAAGGCTAGCGTAAGATCACAAATAAGTGGAGGTAAAGC   900
735_        TGGTTCTCGTCGAAATAGCTTTAAGGCTAGCGTAAGATCACAAATAAGTGGAGGTAAAGC   900
1014        TGGTTCTCGTCGAAATAGCTTTAAGGCTAGCGTAAGATCACAAATAAGTGGAGGTAAAGC   900
122         TGGTTCTCGTCGAAATAGCTTTAAGGCTAGCGTAAGATCACAAATAAGTGGAGGTAAAGC   900
L43967      TGGTTCTCGTCGAAATAGCTTTAAGGCTAGCGTAAGATCACAAATAAGTGGAGGTAAAGC   900
720         TGGTTCTCGTCGAAATAGCTTTAAGGCTAGCGTAAGATCACAAATAAGTGGAGGTAAAGC   900
753         TGGTTCTCGTCGAAATAGCTTTAAGGCTAGCGTAAGATCACAAATAAGTGGAGGTAAAGC   900
1026        TGGTTCTCGTCGAAATAGCTTTAAGGCTAGCGTAAGATCACAAATAAGTGGAGGTAAAGC   900
47          TGGTTCTCGTCGAAATAGCTTTAAGGCTAGCGTAAGATCACAAATAAGTGGAGGTAAAGC   900
92          TGGTTCTCGTCGAAATAGCTTTAAGGCTAGCGTAAGATCACAAATAAGTGGAGGTAAAGC   900
600         TGGTTCTCGTCGAAATAGCTTTAAGGCTAGCGTAAGATCACAAATAAGTGGAGGTAAAGC   900
656         TGGTTCTCGTCGAAATAGCTTTAAGGCTAGCGTAAGATCACAAATAAGTGGAGGTAAAGC   900
            ************************************************************

683         TACTGAATGTATGATGGCGCCACCTAGGCGTACTGAATACAATTAAACTCTGAATGCCAT   960
663         TACTGAATGTATGATGGCGCCACCTAGGCGTACTGAATACAATTAAACTCTGAATGCCAT   960
20          TACTGAATGTATGATGGCGCCACCTAGGCGTACTGAATACAATTAAACTCTGAATGCCAT   960
38          TACTGAATGTATGATGGCGCCACCTAGGCGTACTGAATACAATTAAACTCTGAATGCCAT   960
649         TACTGAATGTATGATGGCGCCACCTAGGCGTACTGAATACAATTAAACTCTGAATGCCAT   960
710         TACTGAATGTATGATGGCGCCACCTAGGCGTACTGAATACAATTAAACTCTGAATGCCAT   960
719         TACTGAATGTATGATGGCGCCACCTAGGCGTACTGAATACAATTAAACTCTGAATGCCAT   960
729         TACTGAATGTATGATGGCGCCACCTAGGCGTACTGAATACAATTAAACTCTGAATGCCAT   960
735_        TACTGAATGTATGATGGCGCCACCTAGGCGTACTGAATACAATTAAACTCTGAATGCCAT   960
1014        TACTGAATGTATGATGGCGCCACCTAGGCGTACTGAATACAATTAAACTCTGAATGCCAT   960
122         TACTGAATGTATGATGGCGCCACCTAGGCGTACTGAATACAATTAAACTCTGAATGCCAT   960
L43967      TACTGAATGTATGATGGCGCCACCTAGGCGTACTGAATACAATTAAACTCTGAATGCCAT   960
720         TACTGAATGTATGATGGCGCCACCTAGGCGTACTGAATACAATTAAACTCTGAATGCCAT   960
753         TACTGAATGTATGATGGCGCCACCTAGGCGTACTGAATACAATTAAACTCTGAATGCCAT   960
1026        TACTGAATGTATGATGGCGCCACCTAGGCGTACTGAATACAATTAAACTCTGAATGCCAT   960
```

```
47          TACTGAATGTATGATGGCGCCACCTAGGCGTACTGAATACAATTAAACTCTGAATGCCAT   960
92          TACTGAATGTATGATGGCGCCACCTAGGCGTACTGAATACAATTAAACTCTGAATGCCAT   960
600         TACTGAATGTATGATGGCGCCACCTAGGCGTACTGAATACAATTAAACTCTGAATGCCAT   960
656         TACTGAATGTATGATGGCGCCACCTAGGCGTACTGAATACAATTAAACTCTGAATGCCAT   960
            ************************************************************

683         TTATTTTATTCTTGCAGTCAGACAGTGGGGGATAAGCTTCATTGTCAAGAGGGGAAGAGC   1020
663         TTATTTTATTCTTGCAGTCAGACAGTGGGGGATAAGCTTCATTGTCAAGAGGGGAAGAGC   1020
20          TTATTTTATTCTTGCAGTCAGACAGTGGGGGATAAGCTTCATTGTCAAGAGGGGAAGAGC   1020
38          TTATTTTATTCTTGCAGTCAGACAGTGGGGGATAAGCTTCATTGTCAAGAGGGGAAGAGC   1020
649         TTATTTTATTCTTGCAGTCAGACAGTGGGGGATAAGCTTCATTGTCAAGAGGGGAAGAGC   1020
710         TTATTTTATTCTTGCAGTCAGACAGTGGGGGATAAGCTTCATTGTCAAGAGGGGAAGAGC   1020
719         TTATTTTATTCTTGCAGTCAGACAGTGGGGGATAAGCTTCATTGTCAAGAGGGGAAGAGC   1020
729         TTATTTTATTCTTGCAGTCAGACAGTGGGGGATAAGCTTCATTGTCAAGAGGGGAAGAGC   1020
735_        TTATTTTATTCTTGCAGTCAGACAGTGGGGGATAAGCTTCATTGTCAAGAGGGGAAGAGC   1020
1014        TTATTTTATTCTTGCAGTCAGACAGTGGGGGATAAGCTTCATTGTCAAGAGGGGAAGAGC   1020
122         TTATTTTATTCTTGCAGTCAGACAGTGGGGGATAAGCTTCATTGTCAAGAGGGGAAGAGC   1020
L43967      TTATTTTATTCTTGCAGTCAGACAGTGGGGGATAAGCTTCATTGTCAAGAGGGGAAGAGC   1020
720         TTATTTTATTCTTGCAGTCAGACAGTGGGGGATAAGCTTCATTGTCAAGAGGGGAAGAGC   1020
753         TTATTTTATTCTTGCAGTCAGACAGTGGGGGATAAGCTTCATTGTCAAGAGGGGAAGAGC   1020
1026        TTATTTTATTCTTGCAGTCAGACAGTGGGGGATAAGCTTCATTGTCAAGAGGGGAAGAGC   1020
47          TTATTTTATTCTTGCAGTCAGACAGTGGGGGATAAGCTTCATTGTCAAGAGGGGAAGAGC   1020
92          TTATTTTATTCTTGCAGTCAGACAGTGGGGGATAAGCTTCATTGTCAAGAGGGGAAGAGC   1020
600         TTATTTTATTCTTGCAGTCAGACAGTGGGGGATAAGCTTCATTGTCAAGAGGGGAAGAGC   1020
656         TTATTTTATTCTTGCAGTCAGACAGTGGGGGATAAGCTTCATTGTCAAGAGGGGAAGAGC   1020
            ************************************************************

683         CCAGATCATTAAATAAGGTCCCCAAAATATACTAAGTGGAAAAGGATGTGAAAGTGCTAA   1080
663         CCAGATCATTAAATAAGGTCCCCAAAATATACTAAGTGGAAAAGGATGTGAAAGTGCTAA   1080
20          CCAGATCATTAAATAAGGTCCCCAAAATATACTAAGTGGAAAAGGATGTGAAAGTGCTAA   1080
38          CCAGATCATTAAATAAGGTCCCCAAAATATACTAAGTGGAAAAGGATGTGAAAGTGCTAA   1080
649         CCAGATCATTAAATAAGGTCCCCAAAATATACTAAGTGGAAAAGGATGTGAAAGTGCTAA   1080
710         CCAGATCATTAAATAAGGTCCCCAAAATATACTAAGTGGAAAAGGATGTGAAAGTGCTAA   1080
719         CCAGATCATTAAATAAGGTCCCCAAAATATACTAAGTGGAAAAGGATGTGAAAGTGCTAA   1080
729         CCAGATCATTAAATAAGGTCCCCAAAATATACTAAGTGGAAAAGGATGTGAAAGTGCTAA   1080
735_        CCAGATCATTAAATAAGGTCCCCAAAATATACTAAGTGGAAAAGGATGTGAAAGTGCTAA   1080
1014        CCAGATCATTAAATAAGGTCCCCAAAATATACTAAGTGGAAAAGGATGTGAAAGTGCTAA   1080
122         CCAGATCATTAAATAAGGTCCCCAAAATATACTAAGTGGAAAAGGATGTGAAAGTGCTAA   1080
L43967      CCAGATCATTAAATAAGGTCCCCAAAATATACTAAGTGGAAAAGGATGTGAAAGTGCTAA   1080
720         CCAGATCATTAAATAAGGTCCCCAAAATATACTAAGTGGAAAAGGATGTGAAAGTGCTAA   1080
753         CCAGATCATTAAATAAGGTCCCCAAAATATACTAAGTGGAAAAGGATGTGAAAGTGCTAA   1080
1026        CCAGATCATTAAATAAGGTCCCCAAAATATACTAAGTGGAAAAGGATGTGAAAGTGCTAA   1080
47          CCAGATCATTAAATAAGGTCCCCAAAATATACTAAGTGGAAAAGGATGTGAAAGTGCTAA   1080
92          CCAGATCATTAAATAAGGTCCCCAAAATATACTAAGTGGAAAAGGATGTGAAAGTGCTAA   1080
600         CCAGATCATTAAATAAGGTCCCCAAAATATACTAAGTGGAAAAGGATGTGAAAGTGCTAA   1080
656         CCAGATCATTAAATAAGGTCCCCAAAATATACTAAGTGGAAAAGGATGTGAAAGTGCTAA   1080
            ************************************************************

683         AACAGCAAGGATGTTGGCTTAGAAGCAGCCATCGTTTAAAGAGTGCGTAACAGCTCACTT   1140
663         AACAGCAAGGATGTTGGCTTAGAAGCAGCCATCGTTTAAAGAGTGCGTAACAGCTCACTT   1140
20          AACAGCAAGGATGTTGGCTTAGAAGCAGCCATCGTTTAAAGAGTGCGTAACAGCTCACTT   1140
38          AACAGCAAGGATGTTGGCTTAGAAGCAGCCATCGTTTAAAGAGTGCGTAACAGCTCACTT   1140
649         AACAGCAAGGATGTTGGCTTAGAAGCAGCCATCGTTTAAAGAGTGCGTAACAGCTCACTT   1140
710         AACAGCAAGGATGTTGGCTTAGAAGCAGCCATCGTTTAAAGAGTGCGTAACAGCTCACTT   1140
719         AACAGCAAGGATGTTGGCTTAGAAGCAGCCATCGTTTAAAGAGTGCGTAACAGCTCACTT   1140
729         AACAGCAAGGATGTTGGCTTAGAAGCAGCCATCGTTTAAAGAGTGCGTAACAGCTCACTT   1140
735_        AACAGCAAGGATGTTGGCTTAGAAGCAGCCATCGTTTAAAGAGTGCGTAACAGCTCACTT   1140
1014        AACAGCAAGGATGTTGGCTTAGAAGCAGCCATCGTTTAAAGAGTGCGTAACAGCTCACTT   1140
122         AACAGCAAGGATGTTGGCTTAGAAGCAGCCATCGTTTAAAGAGTGCGTAACAGCTCACTT   1140
L43967      AACAGCAAGGATGTTGGCTTAGAAGCAGCCATCGTTTAAAGAGTGCGTAACAGCTCACTT   1140
720         AACAGCAAGGATGTTGGCTTAGAAGCAGCCATCGTTTAAAGAGTGCGTAACAGCTCACTT   1140
753         AACAGCAAGGATGTTGGCTTAGAAGCAGCCATCGTTTAAAGAGTGCGTAACAGCTCACTT   1140
1026        AACAGCAAGGATGTTGGCTTAGAAGCAGCCATCGTTTAAAGAGTGCGTAACAGCTCACTT   1140
47          AACAGCAAGGATGTTGGCTTAGAAGCAGCCATCGTTTAAAGAGTGCGTAACAGCTCACTT   1140
92          AACAGCAAGGATGTTGGCTTAGAAGCAGCCATCGTTTAAAGAGTGCGTAACAGCTCACTT   1140
600         AACAGCAAGGATGTTGGCTTAGAAGCAGCCATCGTTTAAAGAGTGCGTAACAGCTCACTT   1140
656         AACAGCAAGGATGTTGGCTTAGAAGCAGCCATCGTTTAAAGAGTGCGTAACAGCTCACTT   1140
            ************************************************************

683         GTCGAGTGTTTTTGCGCCGAAGATGTAACGGGGCTAAGTATATTACCGAATTTATGGATA   1200
663         GTCGAGTGTTTTTGCGCCGAAGATGTAACGGGGCTAAGTATATTACCGAATTTATGGATA   1200
20          GTCGAGTGTTTTTGCGCCGAAGATGTAACGGGGCTAAGTATATTACCGAATTTATGGATA   1200
38          GTCGAGTGTTTTTGCGCCGAAGATGTAACGGGGCTAAGTATATTACCGAATTTATGGATA   1200
649         GTCGAGTGTTTTTGCGCCGAAGATGTAACGGGGCTAAGTATATTACCGAATTTATGGATA   1200
710         GTCGAGTGTTTTTGCGCCGAAGATGTAACGGGGCTAAGTATATTACCGAATTTATGGATA   1200
719         GTCGAGTGTTTTTGCGCCGAAGATGTAACGGGGCTAAGTATATTACCGAATTTATGGATA   1200
729         GTCGAGTGTTTTTGCGCCGAAGATGTAACGGGGCTAAGTATATTACCGAATTTATGGATA   1200
735_        GTCGAGTGTTTTTGCGCCGAAGATGTAACGGGGCTAAGTATATTACCGAATTTATGGATA   1200
1014        GTCGAGTGTTTTTGCGCCGAAGATGTAACGGGGCTAAGTATATTACCGAATTTATGGATA   1200
122         GTCGAGTGTTTTTGCGCCGAAGATGTAACGGGGCTAAGTATATTACCGAATTTATGGATA   1200
L43967      GTCGAGTGTTTTTGCGCCGAAGATGTAACGGGGCTAAGTATATTACCGAATTTATGGATA   1200
720         GTCGAGTGTTTTTGCGCCGAAGATGTAACGGGGCTAAGTATATTACCGAATTTATGGATA   1200
753         GTCGAGTGTTTTTGCGCCGAAGATGTAACGGGGCTAAGTATATTACCGAATTTATGGATA   1200
```

```
1026      GTCGAGTGTTTTTGCGCCGAAGATGTAACGGGGCTAAGTATATTACCGAATTTATGGATA   1200
47        GTCGAGTGTTTTTGCGCCGAAGATGTAACGGGGCTAAGTATATTACCGAATTTATGGATA   1200
92        GTCGAGTGTTTTTGCGCCGAAGATGTAACGGGGCTAAGTATATTACCGAATTTATGGATA   1200
600       GTCGAGTGTTTTTGCGCCGAAGATGTAACGGGGCTAAGTATATTACCGAATTTATGGATA   1200
656       GTCGAGTGTTTTTGCGCCGAAGATGTAACGGGGCTAAGTATATTACCGAATTTATGGATA   1200
          ************************************************************

683       AGATATTTTTATCTTGTGGTAGACGAGCGTTGTATTGGAGTTGAAGTCAAAGCGTGAGCA   1260
663       AGATATTTTTATCTTGTGGTAGACGAGCGTTGTATTGGAGTTGAAGTCAAAGCGTGAGCA   1260
20        AGATATTTTTATCTTGTGGTAGACGAGCGTTGTATTGGAGTTGAAGTCAAAGCGTGAGCA   1260
38        AGATATTTTTATCTTGTGGTAGACGAGCGTTGTATTGGAGTTGAAGTCAAAGCGTGAGCA   1260
649       AGATATTTTTATCTTGTGGTAGACGAGCGTTGTATTGGAGTTGAAGTCAAAGCGTGAGCA   1260
710       AGATATTTTTATCTTGTGGTAGACGAGCGTTGTATTGGAGTTGAAGTCAAAGCGTGAGCA   1260
719       AGATATTTTTATCTTGTGGTAGACGAGCGTTGTATTGGAGTTGAAGTCAAAGCGTGAGCA   1260
729       AGATATTTTTATCTTGTGGTAGACGAGCGTTGTATTGGAGTTGAAGTCAAAGCGTGAGCA   1260
735_      AGATATTTTTATCTTGTGGTAGACGAGCGTTGTATTGGAGTTGAAGTCAAAGCGTGAGCA   1260
1014      AGATATTTTTATCTTGTGGTAGACGAGCGTTGTATTGGAGTTGAAGTCAAAGCGTGAGCA   1260
122       AGATATTTTTATCTTGTGGTAGACGAGCGTTGTATTGGAGTTGAAGTCAAAGCGTGAGCA   1260
L43967    AGATATTTTTATCTTGTGGTAGACGAGCGTTGTATTGGAGTTGAAGTCAAAGCGTGAGCA   1260
720       AGATATTTTTATCTTGTGGTAGACGAGCGTTGTATTGGAGTTGAAGTCAAAGCGTGAGCA   1260
753       AGATATTTTTATCTTGTGGTAGACGAGCGTTGTATTGGAGTTGAAGTCAAAGCGTGAGCA   1260
1026      AGATATTTTTATCTTGTGGTAGACGAGCGTTGTATTGGAGTTGAAGTCAAAGCGTGAGCA   1260
47        AGATATTTTTATCTTGTGGTAGACGAGCGTTGTATTGGAGTTGAAGTCAAAGCGTGAGCA   1260
92        AGATATTTTTATCTTGTGGTAGACGAGCGTTGTATTGGAGTTGAAGTCAAAGCGTGAGCA   1260
600       AGATATTTTTATCTTGTGGTAGACGAGCGTTGTATTGGAGTTGAAGTCAAAGCGTGAGCA   1260
656       AGATATTTTTATCTTGTGGTAGACGAGCGTTGTATTGGAGTTGAAGTCAAAGCGTGAGCA   1260
          ************************************************************

683       TTGGTGGATCCAATACAAGTGAGAATGCCGGCGTGAGTAACGCTTGGGAGTGAGAATCTC   1320
663       TTGGTGGATCCAATACAAGTGAGAATGCCGGCGTGAGTAACGCTTGGGAGTGAGAATCTC   1320
20        TTGGTGGATCCAATACAAGTGAGAATGCCGGCGTGAGTAACGCTTGGGAGTGAGAATCTC   1320
38        TTGGTGGATCCAATACAAGTGAGAATGCCGGCGTGAGTAACGCTTGGGAGTGAGAATCTC   1320
649       TTGGTGGATCCAATACAAGTGAGAATGCCGGCGTGAGTAACGCTTGGGAGTGAGAATCTC   1320
710       TTGGTGGATCCAATACAAGTGAGAATGCCGGCGTGAGTAACGCTTGGGAGTGAGAATCTC   1320
719       TTGGTGGATCCAATACAAGTGAGAATGCCGGCGTGAGTAACGCTTGGGAGTGAGAATCTC   1320
729       TTGGTGGATCCAATACAAGTGAGAATGCCGGCGTGAGTAACGCTTGGGAGTGAGAATCTC   1320
735_      TTGGTGGATCCAATACAAGTGAGAATGCCGGCGTGAGTAACGCTTGGGAGTGAGAATCTC   1320
1014      TTGGTGGATCCAATACAAGTGAGAATGCCGGCGTGAGTAACGCTTGGGAGTGAGAATCTC   1320
122       TTGGTGGATCCAATACAAGTGAGAATGCCGGCGTGAGTAACGCTTGGGAGTGAGAATCTC   1320
L43967    TTGGTGGATCCAATACAAGTGAGAATGCCGGCGTGAGTAACGCTTGGGAGTGAGAATCTC   1320
720       TTGGTGGATCCAATACAAGTGAGAATGCCGGCGTGAGTAACGCTTGGGAGTGAGAATCTC   1320
753       TTGGTGGATCCAATACAAGTGAGAATGCCGGCGTGAGTAACGCTTGGGAGTGAGAATCTC   1320
1026      TTGGTGGATCCAATACAAGTGAGAATGCCGGCGTGAGTAACGCTTGGGAGTGAGAATCTC   1320
47        TTGGTGGATCCAATACAAGTGAGAATGCCGGCGTGAGTAACGCTTGGGAGTGAGAATCTC   1320
92        TTGGTGGATCCAATACAAGTGAGAATGCCGGCGTGAGTAACGCTTGGGAGTGAGAATCTC   1320
600       TTGGTGGATCCAATACAAGTGAGAATGCCGGCGTGAGTAACGCTTGGGAGTGAGAATCTC   1320
656       TTGGTGGATCCAATACAAGTGAGAATGCCGGCGTGAGTAACGCTTGGGAGTGAGAATCTC   1320
          ************************************************************

683       CCAAACCGATTGACTAAGGTTTCCTGGACCAGGGTCGTCCTTCCAGGGTTAGTCTGGACC   1380
663       CCAAACCGATTGACTAAGGTTTCCTGGACCAGGGTCGTCCTTCCAGGGTTAGTCTGGACC   1380
20        CCAAACCGATTGACTAAGGTTTCCTGGACCAGGGTCGTCCTTCCAGGGTTAGTCTGGACC   1380
38        CCAAACCGATTGACTAAGGTTTCCTGGACCAGGGTCGTCCTTCCAGGGTTAGTCTGGACC   1380
649       CCAAACCGATTGACTAAGGTTTCCTGGACCAGGGTCGTCCTTCCAGGGTTAGTCTGGACC   1380
710       CCAAACCGATTGACTAAGGTTTCCTGGACCAGGGTCGTCCTTCCAGGGTTAGTCTGGACC   1380
719       CCAAACCGATTGACTAAGGTTTCCTGGACCAGGGTCGTCCTTCCAGGGTTAGTCTGGACC   1380
729       CCAAACCGATTGACTAAGGTTTCCTGGACCAGGGTCGTCCTTCCAGGGTTAGTCTGGACC   1380
735_      CCAAACCGATTGACTAAGGTTTCCTGGACCAGGGTCGTCCTTCCAGGGTTAGTCTGGACC   1380
1014      CCAAACCGATTGACTAAGGTTTCCTGGACCAGGGTCGTCCTTCCAGGGTTAGTCTGGACC   1380
122       CCAAACCGATTGACTAAGGTTTCCTGGACCAGGGTCGTCCTTCCAGGGTTAGTCTGGACC   1380
L43967    CCAAACCGATTGACTAAGGTTTCCTGGACCAGGGTCGTCCTTCCAGGGTTAGTCTGGACC   1380
720       CCAAACCGATTGACTAAGGTTTCCTGGACCAGGGTCGTCCTTCCAGGGTTAGTCTGGACC   1380
753       CCAAACCGATTGACTAAGGTTTCCTGGACCAGGGTCGTCCTTCCAGGGTTAGTCTGGACC   1380
1026      CCAAACCGATTGACTAAGGTTTCCTGGACCAGGGTCGTCCTTCCAGGGTTAGTCTGGACC   1380
47        CCAAACCGATTGACTAAGGTTTCCTGGACCAGGGTCGTCCTTCCAGGGTTAGTCTGGACC   1380
92        CCAAACCGATTGACTAAGGTTTCCTGGACCAGGGTCGTCCTTCCAGGGTTAGTCTGGACC   1380
600       CCAAACCGATTGACTAAGGTTTCCTGGACCAGGGTCGTCCTTCCAGGGTTAGTCTGGACC   1380
656       CCAAACCGATTGACTAAGGTTTCCTGGACCAGGGTCGTCCTTCCAGGGTTAGTCTGGACC   1380
          ************************************************************

683       TAAGCTGAGGCTGAAGAGCGTAGGCGATGGACAACAGGTTAATATTCCTGTACTTACAGT   1440
663       TAAGCTGAGGCTGAAGAGCGTAGGCGATGGACAACAGGTTAATATTCCTGTACTTACAGT   1440
20        TAAGCTGAGGCTGAAGAGCGTAGGCGATGGACAACAGGTTAATATTCCTGTACTTACAGT   1440
38        TAAGCTGAGGCTGAAGAGCGTAGGCGATGGACAACAGGTTAATATTCCTGTACTTACAGT   1440
649       TAAGCTGAGGCTGAAGAGCGTAGGCGATGGACAACAGGTTAATATTCCTGTACTTACAGT   1440
710       TAAGCTGAGGCTGAAGAGCGTAGGCGATGGACAACAGGTTAATATTCCTGTACTTACAGT   1440
719       TAAGCTGAGGCTGAAGAGCGTAGGCGATGGACAACAGGTTAATATTCCTGTACTTACAGT   1440
729       TAAGCTGAGGCTGAAGAGCGTAGGCGATGGACAACAGGTTAATATTCCTGTACTTACAGT   1440
735_      TAAGCTGAGGCTGAAGAGCGTAGGCGATGGACAACAGGTTAATATTCCTGTACTTACAGT   1440
1014      TAAGCTGAGGCTGAAGAGCGTAGGCGATGGACAACAGGTTAATATTCCTGTACTTACAGT   1440
122       TAAGCTGAGGCTGAAGAGCGTAGGCGATGGACAACAGGTTAATATTCCTGTACTTACAGT   1440
L43967    TAAGCTGAGGCTGAAGAGCGTAGGCGATGGACAACAGGTTAATATTCCTGTACTTACAGT   1440
720       TAAGCTGAGGCTGAAGAGCGTAGGCGATGGACAACAGGTTAATATTCCTGTACTTACAGT   1440
```

```
753        TAAGCTGAGGCTGAAGAGCGTAGGCGATGGACAACAGGTTAATATTCCTGTACTTACAGT   1440
1026       TAAGCTGAGGCTGAAGAGCGTAGGCGATGGACAACAGGTTAATATTCCTGTACTTACAGT   1440
47         TAAGCTGAGGCTGAAGAGCGTAGGCGATGGACAACAGGTTAATATTCCTGTACTTACAGT   1440
92         TAAGCTGAGGCTGAAGAGCGTAGGCGATGGACAACAGGTTAATATTCCTGTACTTACAGT   1440
600        TAAGCTGAGGCTGAAGAGCGTAGGCGATGGACAACAGGTTAATATTCCTGTACTTACAGT   1440
656        TAAGCTGAGGCTGAAGAGCGTAGGCGATGGACAACAGGTTAATATTCCTGTACTTACAGT   1440
           ************************************************************

683        TAGACTGATGGAGTGACAAAGAAGGTTATCCACCCCCATTATTGGATTTGGGGATAAACC   1500
663        TAGACTGATGGAGTGACAAAGAAGGTTATCCACCCCCATTATTGGATTTGGGGATAAACC   1500
20         TAGACTGATGGAGTGACAAAGAAGGTTATCCACCCCCATTATTGGATTTGGGGATAAACC   1500
38         TAGACTGATGGAGTGACAAAGAAGGTTATCCACCCCCATTATTGGATTTGGGGATAAACC   1500
649        TAGACTGATGGAGTGACAAAGAAGGTTATCCACCCCCATTATTGGATTTGGGGATAAACC   1500
710        TAGACTGATGGAGTGACAAAGAAGGTTATCCACCCCCATTATTGGATTTGGGGATAAACC   1500
719        TAGACTGATGGAGTGACAAAGAAGGTTATCCACCCCCATTATTGGATTTGGGGATAAACC   1500
729        TAGACTGATGGAGTGACAAAGAAGGTTATCCACCCCCATTATTGGATTTGGGGATAAACC   1500
735_       TAGACTGATGGAGTGACAAAGAAGGTTATCCACCCCCATTATTGGATTTGGGGATAAACC   1500
1014       TAGACTGATGGAGTGACAAAGAAGGTTATCCACCCCCATTATTGGATTTGGGGATAAACC   1500
122        TAGACTGATGGAGTGACAAAGAAGGTTATCCACCCCCATTATTGGATTTGGGGATAAACC   1500
L43967     TAGACTGATGGAGTGACAAAGAAGGTTATCCACCCCCATTATTGGATTTGGGGATAAACC   1500
720        TAGACTGATGGAGTGACAAAGAAGGTTATCCACCCCCATTATTGGATTTGGGGATAAACC   1500
753        TAGACTGATGGAGTGACAAAGAAGGTTATCCACCCCCATTATTGGATTTGGGGATAAACC   1500
1026       TAGACTGATGGAGTGACAAAGAAGGTTATCCACCCCCATTATTGGATTTGGGGATAAACC   1500
47         TAGACTGATGGAGTGACAAAGAAGGTTATCCACCCCCATTATTGGATTTGGGGATAAACC   1500
92         TAGACTGATGGAGTGACAAAGAAGGTTATCCACCCCCATTATTGGATTTGGGGATAAACC   1500
600        TAGACTGATGGAGTGACAAAGAAGGTTATCCACCCCCATTATTGGATTTGGGGATAAACC   1500
656        TAGACTGATGGAGTGACAAAGAAGGTTATCCACCCCCATTATTGGATTTGGGGATAAACC   1500
           ************************************************************

683        ATAAGGTGGTACAATAGGCAAATCCGTTGTGCATAACACTGAGTGGTGATGTCGAGTGAA   1560
663        ATAAGGTGGTACAATAGGCAAATCCGTTGTGCATAACACTGAGTGGTGATGTCGAGTGAA   1560
20         ATAAGGTGGTACAATAGGCAAATCCGTTGTGCATAACACTGAGTGGTGATGTCGAGTGAA   1560
38         ATAAGGTGGTACAATAGGCAAATCCGTTGTGCATAACACTGAGTGGTGATGTCGAGTGAA   1560
649        ATAAGGTGGTACAATAGGCAAATCCGTTGTGCATAACACTGAGTGGTGATGTCGAGTGAA   1560
710        ATAAGGTGGTACAATAGGCAAATCCGTTGTGCATAACACTGAGTGGTGATGTCGAGTGAA   1560
719        ATAAGGTGGTACAATAGGCAAATCCGTTGTGCATAACACTGAGTGGTGATGTCGAGTGAA   1560
729        ATAAGGTGGTACAATAGGCAAATCCGTTGTGCATAACACTGAGTGGTGATGTCGAGTGAA   1560
735_       ATAAGGTGGTACAATAGGCAAATCCGTTGTGCATAACACTGAGTGGTGATGTCGAGTGAA   1560
1014       ATAAGGTGGTACAATAGGCAAATCCGTTGTGCATAACACTGAGTGGTGATGTCGAGTGAA   1560
122        ATAAGGTGGTACAATAGGCAAATCCGTTGTGCATAACACTGAGTGGTGATGTCGAGTGAA   1560
L43967     ATAAGGTGGTACAATAGGCAAATCCGTTGTGCATAACACTGAGTGGTGATGTCGAGTGAA   1560
720        ATAAGGTGGTACAATAGGCAAATCCGTTGTGCATAACACTGAGTGGTGATGTCGAGTGAA   1560
753        ATAAGGTGGTACAATAGGCAAATCCGTTGTGCATAACACTGAGTGGTGATGTCGAGTGAA   1560
1026       ATAAGGTGGTACAATAGGCAAATCCGTTGTGCATAACACTGAGTGGTGATGTCGAGTGAA   1560
47         ATAAGGTGGTACAATAGGCAAATCCGTTGTGCATAACACTGAGTGGTGATGTCGAGTGAA   1560
92         ATAAGGTGGTACAATAGGCAAATCCGTTGTGCATAACACTGAGTGGTGATGTCGAGTGAA   1560
600        ATAAGGTGGTACAATAGGCAAATCCGTTGTGCATAACACTGAGTGGTGATGTCGAGTGAA   1560
656        ATAAGGTGGTACAATAGGCAAATCCGTTGTGCATAACACTGAGTGGTGATGTCGAGTGAA   1560
           ************************************************************

683        CGAGTGATCAAGTAGCGAAGGTGGCAATTAATCATGCTTTCAAGAAAAGCTGCTAGGGCT   1620
663        CGAGTGATCAAGTAGCGAAGGTGGCAATTAATCATGCTTTCAAGAAAAGCTGCTAGGGCT   1620
20         CGAGTGATCAAGTAGCGAAGGTGGCAATTAATCATGCTTTCAAGAAAAGCTGCTAGGGCT   1620
38         CGAGTGATCAAGTAGCGAAGGTGGCAATTAATCATGCTTTCAAGAAAAGCTGCTAGGGCT   1620
649        CGAGTGATCAAGTAGCGAAGGTGGCAATTAATCATGCTTTCAAGAAAAGCTGCTAGGGCT   1620
710        CGAGTGATCAAGTAGCGAAGGTGGCAATTAATCATGCTTTCAAGAAAAGCTGCTAGGGCT   1620
719        CGAGTGATCAAGTAGCGAAGGTGGCAATTAATCATGCTTTCAAGAAAAGCTGCTAGGGCT   1620
729        CGAGTGATCAAGTAGCGAAGGTGGCAATTAATCATGCTTTCAAGAAAAGCTGCTAGGGCT   1620
735_       CGAGTGATCAAGTAGCGAAGGTGGCAATTAATCATGCTTTCAAGAAAAGCTGCTAGGGCT   1620
1014       CGAGTGATCAAGTAGCGAAGGTGGCAATTAATCATGCTTTCAAGAAAAGCTGCTAGGGCT   1620
122        CGAGTGATCAAGTAGCGAAGGTGGCAATTAATCATGCTTTCAAGAAAAGCTGCTAGGGCT   1620
L43967     CGAGTGATCAAGTAGCGAAGGTGGCAATTAATCATGCTTTCAAGAAAAGCTGCTAGGGCT   1620
720        CGAGTGATCAAGTAGCGAAGGTGGCAATTAATCATGCTTTCAAGAAAAGCTGCTAGGGCT   1620
753        CGAGTGATCAAGTAGCGAAGGTGGCAATTAATCATGCTTTCAAGAAAAGCTGCTAGGGCT   1620
1026       CGAGTGATCAAGTAGCGAAGGTGGCAATTAATCATGCTTTCAAGAAAAGCTGCTAGGGCT   1620
47         CGAGTGATCAAGTAGCGAAGGTGGCAATTAATCATGCTTTCAAGAAAAGCTGCTAGGGCT   1620
92         CGAGTGATCAAGTAGCGAAGGTGGCAATTAATCATGCTTTCAAGAAAAGCTGCTAGGGCT   1620
600        CGAGTGATCAAGTAGCGAAGGTGGCAATTAATCATGCTTTCAAGAAAAGCTGCTAGGGCT   1620
656        CGAGTGATCAAGTAGCGAAGGTGGCAATTAATCATGCTTTCAAGAAAAGCTGCTAGGGCT   1620
           ************************************************************

683        AATTTAACTGTAACCAGTACCGAGAACGAACACACGTAGTCAAGGAGAGGATCCTAAGGT   1680
663        AATTTAACTGTAACCAGTACCGAGAACGAACACACGTAGTCAAGGAGAGGATCCTAAGGT   1680
20         AATTTAACTGTAACCAGTACCGAGAACGAACACACGTAGTCAAGGAGAGGATCCTAAGGT   1680
38         AATTTAACTGTAACCAGTACCGAGAACGAACACACGTAGTCAAGGAGAGGATCCTAAGGT   1680
649        AATTTAACTGTAACCAGTACCGAGAACGAACACACGTAGTCAAGGAGAGGATCCTAAGGT   1680
710        AATTTAACTGTAACCAGTACCGAGAACGAACACACGTAGTCAAGGAGAGGATCCTAAGGT   1680
719        AATTTAACTGTAACCAGTACCGAGAACGAACACACGTAGTCAAGGAGAGGATCCTAAGGT   1680
729        AATTTAACTGTAACCAGTACCGAGAACGAACACACGTAGTCAAGGAGAGGATCCTAAGGT   1680
735_       AATTTAACTGTAACCAGTACCGAGAACGAACACACGTAGTCAAGGAGAGGATCCTAAGGT   1680
1014       AATTTAACTGTAACCAGTACCGAGAACGAACACACGTAGTCAAGGAGAGGATCCTAAGGT   1680
122        AATTTAACTGTAACCAGTACCGAGAACGAACACACGTAGTCAAGGAGAGGATCCTAAGGT   1680
L43967     AATTTAACTGTAACCAGTACCGAGAACGAACACACGTAGTCAAGGAGAGGATCCTAAGGT   1680
```

```
720        AATTTAACTGTAACCAGTACCGAGAACGAACACACGTAGTCAAGGAGAGGATCCTAAGGT   1680
753        AATTTAACTGTAACCAGTACCGAGAACGAACACACGTAGTCAAGGAGAGGATCCTAAGGT   1680
1026       AATTTAACTGTAACCAGTACCGAGAACGAACACACGTAGTCAAGGAGAGGATCCTAAGGT   1680
47         AATTTAACTGTAACCAGTACCGAGAACGAACACACGTAGTCAAGGAGAGGATCCTAAGGT   1680
92         AATTTAACTGTAACCAGTACCGAGAACGAACACACGTAGTCAAGGAGAGGATCCTAAGGT   1680
600        AATTTAACTGTAACCAGTACCGAGAACGAACACACGTAGTCAAGGAGAGGATCCTAAGGT   1680
656        AATTTAACTGTAACCAGTACCGAGAACGAACACACGTAGTCAAGGAGAGGATCCTAAGGT   1680
           ************************************************************

683        TAGCGAGTGAACTATAGCCAAGGAACTCTGCAAATTAACCCCGTAAGTTAGCGAGAAGGG   1740
663        TAGCGAGTGAACTATAGCCAAGGAACTCTGCAAATTAACCCCGTAAGTTAGCGAGAAGGG   1740
20         TAGCGAGTGAACTATAGCCAAGGAACTCTGCAAATTAACCCCGTAAGTTAGCGAGAAGGG   1740
38         TAGCGAGTGAACTATAGCCAAGGAACTCTGCAAATTAACCCCGTAAGTTAGCGAGAAGGG   1740
649        TAGCGAGTGAACTATAGCCAAGGAACTCTGCAAATTAACCCCGTAAGTTAGCGAGAAGGG   1740
710        TAGCGAGTGAACTATAGCCAAGGAACTCTGCAAATTAACCCCGTAAGTTAGCGAGAAGGG   1740
719        TAGCGAGTGAACTATAGCCAAGGAACTCTGCAAATTAACCCCGTAAGTTAGCGAGAAGGG   1740
729        TAGCGAGTGAACTATAGCCAAGGAACTCTGCAAATTAACCCCGTAAGTTAGCGAGAAGGG   1740
735_       TAGCGAGTGAACTATAGCCAAGGAACTCTGCAAATTAACCCCGTAAGTTAGCGAGAAGGG   1740
1014       TAGCGAGTGAACTATAGCCAAGGAACTCTGCAAATTAACCCCGTAAGTTAGCGAGAAGGG   1740
122        TAGCGAGTGAACTATAGCCAAGGAACTCTGCAAATTAACCCCGTAAGTTAGCGAGAAGGG   1740
L43967     TAGCGAGTGAACTATAGCCAAGGAACTCTGCAAATTAACCCCGTAAGTTAGCGAGAAGGG   1740
720        TAGCGAGTGAACTATAGCCAAGGAACTCTGCAAATTAACCCCGTAAGTTAGCGAGAAGGG   1740
753        TAGCGAGTGAACTATAGCCAAGGAACTCTGCAAATTAACCCCGTAAGTTAGCGAGAAGGG   1740
1026       TAGCGAGTGAACTATAGCCAAGGAACTCTGCAAATTAACCCCGTAAGTTAGCGAGAAGGG   1740
47         TAGCGAGTGAACTATAGCCAAGGAACTCTGCAAATTAACCCCGTAAGTTAGCGAGAAGGG   1740
92         TAGCGAGTGAACTATAGCCAAGGAACTCTGCAAATTAACCCCGTAAGTTAGCGAGAAGGG   1740
600        TAGCGAGTGAACTATAGCCAAGGAACTCTGCAAATTAACCCCGTAAGTTAGCGAGAAGGG   1740
656        TAGCGAGTGAACTATAGCCAAGGAACTCTGCAAATTAACCCCGTAAGTTAGCGAGAAGGG   1740
           ************************************************************

683        GTGCTTATCTAAAAGTAAGCCGCAGTGAAGAACGAGGGGGGACTGTTTAACTAAAACACA   1800
663        GTGCTTATCTAAAAGTAAGCCGCAGTGAAGAACGAGGGGGGACTGTTTAACTAAAACACA   1800
20         GTGCTTATCTAAAAGTAAGCCGCAGTGAAGAACGAGGGGGGACTGTTTAACTAAAACACA   1800
38         GTGCTTATCTAAAAGTAAGCCGCAGTGAAGAACGAGGGGGGACTGTTTAACTAAAACACA   1800
649        GTGCTTATCTAAAAGTAAGCCGCAGTGAAGAACGAGGGGGGACTGTTTAACTAAAACACA   1800
710        GTGCTTATCTAAAAGTAAGCCGCAGTGAAGAACGAGGGGGGACTGTTTAACTAAAACACA   1800
719        GTGCTTATCTAAAAGTAAGCCGCAGTGAAGAACGAGGGGGGACTGTTTAACTAAAACACA   1800
729        GTGCTTATCTAAAAGTAAGCCGCAGTGAAGAACGAGGGGGGACTGTTTAACTAAAACACA   1800
735_       GTGCTTATCTAAAAGTAAGCCGCAGTGAAGAACGAGGGGGGACTGTTTAACTAAAACACA   1800
1014       GTGCTTATCTAAAAGTAAGCCGCAGTGAAGAACGAGGGGGGACTGTTTAACTAAAACACA   1800
122        GTGCTTATCTAAAAGTAAGCCGCAGTGAAGAACGAGGGGGGACTGTTTAACTAAAACACA   1800
L43967     GTGCTTATCTAAAAGTAAGCCGCAGTGAAGAACGAGGGGGGACTGTTTAACTAAAACACA   1800
720        GTGCTTATCTAAAAGTAAGCCGCAGTGAAGAACGAGGGGGGACTGTTTAACTAAAACACA   1800
753        GTGCTTATCTAAAAGTAAGCCGCAGTGAAGAACGAGGGGGGACTGTTTAACTAAAACACA   1800
1026       GTGCTTATCTAAAAGTAAGCCGCAGTGAAGAACGAGGGGGGACTGTTTAACTAAAACACA   1800
47         GTGCTTATCTAAAAGTAAGCCGCAGTGAAGAACGAGGGGGGACTGTTTAACTAAAACACA   1800
92         GTGCTTATCTAAAAGTAAGCCGCAGTGAAGAACGAGGGGGGACTGTTTAACTAAAACACA   1800
600        GTGCTTATCTAAAAGTAAGCCGCAGTGAAGAACGAGGGGGGACTGTTTAACTAAAACACA   1800
656        GTGCTTATCTAAAAGTAAGCCGCAGTGAAGAACGAGGGGGGACTGTTTAACTAAAACACA   1800
           ************************************************************

683        ACTCTATGCCAAACCGTAAGGTGATGTATATGGGGTGACACCTGCCCAGTGCTGGAAGGT   1860
663        ACTCTATGCCAAACCGTAAGGTGATGTATATGGGGTGACACCTGCCCAGTGCTGGAAGGT   1860
20         ACTCTATGCCAAACCGTAAGGTGATGTATATGGGGTGACACCTGCCCAGTGCTGGAAGGT   1860
38         ACTCTATGCCAAACCGTAAGGTGATGTATATGGGGTGACACCTGCCCAGTGCTGGAAGGT   1860
649        ACTCTATGCCAAACCGTAAGGTGATGTATATGGGGTGACACCTGCCCAGTGCTGGAAGGT   1860
710        ACTCTATGCCAAACCGTAAGGTGATGTATATGGGGTGACACCTGCCCAGTGCTGGAAGGT   1860
719        ACTCTATGCCAAACCGTAAGGTGATGTATATGGGGTGACACCTGCCCAGTGCTGGAAGGT   1860
729        ACTCTATGCCAAACCGTAAGGTGATGTATATGGGGTGACACCTGCCCAGTGCTGGAAGGT   1860
735_       ACTCTATGCCAAACCGTAAGGTGATGTATATGGGGTGACACCTGCCCAGTGCTGGAAGGT   1860
1014       ACTCTATGCCAAACCGTAAGGTGATGTATATGGGGTGACACCTGCCCAGTGCTGGAAGGT   1860
122        ACTCTATGCCAAACCGTAAGGTGATGTATATGGGGTGACACCTGCCCAGTGCTGGAAGGT   1860
L43967     ACTCTATGCCAAACCGTAAGGTGATGTATATGGGGTGACACCTGCCCAGTGCTGGAAGGT   1860
720        ACTCTATGCCAAACCGTAAGGTGATGTATATGGGGTGACACCTGCCCAGTGCTGGAAGGT   1860
753        ACTCTATGCCAAACCGTAAGGTGATGTATATGGGGTGACACCTGCCCAGTGCTGGAAGGT   1860
1026       ACTCTATGCCAAACCGTAAGGTGATGTATATGGGGTGACACCTGCCCAGTGCTGGAAGGT   1860
47         ACTCTATGCCAAACCGTAAGGTGATGTATATGGGGTGACACCTGCCCAGTGCTGGAAGGT   1860
92         ACTCTATGCCAAACCGTAAGGTGATGTATATGGGGTGACACCTGCCCAGTGCTGGAAGGT   1860
600        ACTCTATGCCAAACCGTAAGGTGATGTATATGGGGTGACACCTGCCCAGTGCTGGAAGGT   1860
656        ACTCTATGCCAAACCGTAAGGTGATGTATATGGGGTGACACCTGCCCAGTGCTGGAAGGT   1860
           ************************************************************

683        TAAAGAAGGAGGTTAGCAATTTATTGCAAAGCTTTTAACTGAAGCCCCAGTGAACGGCGG   1920
663        TAAAGAAGGAGGTTAGCAATTTATTGCAAAGCTTTTAACTGAAGCCCCAGTGAACGGCGG   1920
20         TAAAGAAGGAGGTTAGCAATTTATTGCAAAGCTTTTAACTGAAGCCCCAGTGAACGGCGG   1920
38         TAAAGAAGGAGGTTAGCAATTTATTGCAAAGCTTTTAACTGAAGCCCCAGTGAACGGCGG   1920
649        TAAAGAAGGAGGTTAGCAATTTATTGCAAAGCTTTTAACTGAAGCCCCAGTGAACGGCGG   1920
710        TAAAGAAGGAGGTTAGCAATTTATTGCAAAGCTTTTAACTGAAGCCCCAGTGAACGGCGG   1920
719        TAAAGAAGGAGGTTAGCAATTTATTGCAAAGCTTTTAACTGAAGCCCCAGTGAACGGCGG   1920
729        TAAAGAAGGAGGTTAGCAATTTATTGCAAAGCTTTTAACTGAAGCCCCAGTGAACGGCGG   1920
735_       TAAAGAAGGAGGTTAGCAATTTATTGCAAAGCTTTTAACTGAAGCCCCAGTGAACGGCGG   1920
1014       TAAAGAAGGAGGTTAGCAATTTATTGCAAAGCTTTTAACTGAAGCCCCAGTGAACGGCGG   1920
122        TAAAGAAGGAGGTTAGCAATTTATTGCAAAGCTTTTAACTGAAGCCCCAGTGAACGGCGG   1920
```

```
L43967      TAAAGAAGGAGGTTAGCAATTTATTGCAAAGCTTTTAACTGAAGCCCCAGTGAACGGCGG   1920
720         TAAAGAAGGAGGTTAGCAATTTATTGCAAAGCTTTTAACTGAAGCCCCAGTGAACGGCGG   1920
753         TAAAGAAGGAGGTTAGCAATTTATTGCAAAGCTTTTAACTGAAGCCCCAGTGAACGGCGG   1920
1026        TAAAGAAGGAGGTTAGCAATTTATTGCAAAGCTTTTAACTGAAGCCCCAGTGAACGGCGG   1920
47          TAAAGAAGGAGGTTAGCAATTTATTGCAAAGCTTTTAACTGAAGCCCCAGTGAACGGCGG   1920
92          TAAAGAAGGAGGTTAGCAATTTATTGCAAAGCTTTTAACTGAAGCCCCAGTGAACGGCGG   1920
600         TAAAGAAGGAGGTTAGCAATTTATTGCAAAGCTTTTAACTGAAGCCCCAGTGAACGGCGG   1920
656         TAAAGAAGGAGGTTAGCAATTTATTGCAAAGCTTTTAACTGAAGCCCCAGTGAACGGCGG   1920
            ************************************************************

683         CCGTAACTATAACGGTCCTAAGGTAGCGAAATTCCTAGTCGGGTAAATTCCGTCCCGCTT   1980
663         CCGTAACTATAACGGTCCTAAGGTAGCGAAATTCCTAGTCGGGTAAATTCCGTCCCGCTT   1980
20          CCGTAACTATAACGGTCCTAAGGTAGCGAAATTCCTAGTCGGGTAAATTCCGTCCCGCTT   1980
38          CCGTAACTATAACGGTCCTAAGGTAGCGAAATTCCTAGTCGGGTAAATTCCGTCCCGCTT   1980
649         CCGTAACTATAACGGTCCTAAGGTAGCGAAATTCCTAGTCGGGTAAATTCCGTCCCGCTT   1980
710         CCGTAACTATAACGGTCCTAAGGTAGCGAAATTCCTAGTCGGGTAAATTCCGTCCCGCTT   1980
719         CCGTAACTATAACGGTCCTAAGGTAGCGAAATTCCTAGTCGGGTAAATTCCGTCCCGCTT   1980
729         CCGTAACTATAACGGTCCTAAGGTAGCGAAATTCCTAGTCGGGTAAATTCCGTCCCGCTT   1980
735_        CCGTAACTATAACGGTCCTAAGGTAGCGAAATTCCTAGTCGGGTAAATTCCGTCCCGCTT   1980
1014        CCGTAACTATAACGGTCCTAAGGTAGCGAAATTCCTAGTCGGGTAAATTCCGTCCCGCTT   1980
122         CCGTAACTATAACGGTCCTAAGGTAGCGAAATTCCTAGTCGGGTAAATTCCGTCCCGCTT   1980
L43967      CCGTAACTATAACGGTCCTAAGGTAGCGAAATTCCTAGTCGGGTAAATTCCGTCCCGCTT   1980
720         CCGTAACTATAACGGTCCTAAGGTAGCGAAATTCCTAGTCGGGTAAATTCCGTCCCGCTT   1980
753         CCGTAACTATAACGGTCCTAAGGTAGCGAAATTCCTAGTCGGGTAAATTCCGTCCCGCTT   1980
1026        CCGTAACTATAACGGTCCTAAGGTAGCGAAATTCCTAGTCGGGTAAATTCCGTCCCGCTT   1980
47          CCGTAACTATAACGGTCCTAAGGTAGCGAAATTCCTAGTCGGGTAAATTCCGTCCCGCTT   1980
92          CCGTAACTATAACGGTCCTAAGGTAGCGAAATTCCTAGTCGGGTAAATTCCGTCCCGCTT   1980
600         CCGTAACTATAACGGTCCTAAGGTAGCGAAATTCCTAGTCGGGTAAATTCCGTCCCGCTT   1980
656         CCGTAACTATAACGGTCCTAAGGTAGCGAAATTCCTAGTCGGGTAAATTCCGTCCCGCTT   1980
            ************************************************************

683         GAATGGTGTAACCATCTCTTGACTGTCTCGGCTATAGACTCGGTGAAATCCAGGTACGGG   2040
663         GAATGGTGTAACCATCTCTTGACTGTCTCGGCTATAGACTCGGTGAAATCCAGGTACGGG   2040
20          GAATGGTGTAACCATCTCTTGACTGTCTCGGCTATAGACTCGGTGAAATCCAGGTACGGG   2040
38          GAATGGTGTAACCATCTCTTGACTGTCTCGGCTATAGACTCGGTGAAATCCAGGTACGGG   2040
649         GAATGGTGTAACCATCTCTTGACTGTCTCGGCTATAGACTCGGTGAAATCCAGGTACGGG   2040
710         GAATGGTGTAACCATCTCTTGACTGTCTCGGCTATAGACTCGGTGAAATCCAGGTACGGG   2040
719         GAATGGTGTAACCATCTCTTGACTGTCTCGGCTATAGACTCGGTGAAATCCAGGTACGGG   2040
729         GAATGGTGTAACCATCTCTTGACTGTCTCGGCTATAGACTCGGTGAAATCCAGGTACGGG   2040
735_        GAATGGTGTAACCATCTCTTGACTGTCTCGGCTATAGACTCGGTGAAATCCAGGTACGGG   2040
1014        GAATGGTGTAACCATCTCTTGACTGTCTCGGCTATAGACTCGGTGAAATCCAGGTACGGG   2040
122         GAATGGTGTAACCATCTCTTGACTGTCTCGGCTATAGACTCGGTGAAATCCAGGTACGGG   2040
L43967      GAATGGTGTAACCATCTCTTGACTGTCTCGGCTATAGACTCGGTGAAATCCAGGTACGGG   2040
720         GAATGGTGTAACCATCTCTTGACTGTCTCGGCTATAGACTCGGTGAAATCCAGGTACGGG   2040
753         GAATGGTGTAACCATCTCTTGACTGTCTCGGCTATAGACTCGGTGAAATCCAGGTACGGG   2040
1026        GAATGGTGTAACCATCTCTTGACTGTCTCGGCTATAGACTCGGTGAAATCCAGGTACGGG   2040
47          GAATGGTGTAACCATCTCTTGACTGTCTCGGCTATAGACTCGGTGAAATCCAGGTACGGG   2040
92          GAATGGTGTAACCATCTCTTGACTGTCTCGGCTATAGACTCGGTGAAATCCAGGTACGGG   2040
600         GAATGGTGTAACCATCTCTTGACTGTCTCGGCTATAGACTCGGTGAAATCCAGGTACGGG   2040
656         GAATGGTGTAACCATCTCTTGACTGTCTCGGCTATAGACTCGGTGAAATCCAGGTACGGG   2040
            ************************************************************

683         TGAAGACACCCGTTAGGCGCAACGGGACGGGAAGACCCCGTGAAGCTTTACTGTAGCTTA   2100
663         TGAAGACACCCGTTAGGCGCAACGGGACGGAGAGACCCCGTGAAGCTTTACTGTAGCTTA   2100
20          TGAAGACACCCGTTAGGCGCAACGGGACGGAGAGACCCCGTGAAGCTTTACTGTAGCTTA   2100
38          TGAAGACACCCGTTAGGCGCAACGGGACGGAGAGACCCCGTGAAGCTTTACTGTAGCTTA   2100
649         TGAAGACACCCGTTAGGCGCAACGGGACGGAGAGACCCCGTGAAGCTTTACTGTAGCTTA   2100
710         TGAAGACACCCGTTAGGCGCAACGGGACGGAGAGACCCCGTGAAGCTTTACTGTAGCTTA   2100
719         TGAAGACACCCGTTAGGCGCAACGGGACGGAGAGACCCCGTGAAGCTTTACTGTAGCTTA   2100
729         TGAAGACACCCGTTAGGCGCAACGGGACGGAGAGACCCCGTGAAGCTTTACTGTAGCTTA   2100
735_        TGAAGACACCCGTTAGGCGCAACGGGACGGAGAGACCCCGTGAAGCTTTACTGTAGCTTA   2100
1014        TGAAGACACCCGTTAGGCGCAACGGGACGGAGAGACCCCGTGAAGCTTTACTGTAGCTTA   2100
122         TGAAGACACCCGTTAGGCGCAACGGGACGGAAAGACCCCGTGAAGCTTTACTGTAGCTTA   2100
L43967      TGAAGACACCCGTTAGGCGCAACGGGACGGAAAGACCCCGTGAAGCTTTACTGTAGCTTA   2100
720         TGAAGACACCCGTTAGGCGCAACGGGACGGTAAGACCCCGTGAAGCTTTACTGTAGCTTA   2100
753         TGAAGACACCCGTTAGGCGCAACGGGACGGTAAGACCCCGTGAAGCTTTACTGTAGCTTA   2100
1026        TGAAGACACCCGTTAGGCGCAACGGGACGGTAAGACCCCGTGAAGCTTTACTGTAGCTTA   2100
47          TGAAGACACCCGTTAGGCGCAACGGGACGGGAAGACCCCGTGAAGCTTTACTGTAGCTTA   2100
92          TGAAGACACCCGTTAGGCGCAACGGGACGGGAAGACCCCGTGAAGCTTTACTGTAGCTTA   2100
600         TGAAGACACCCGTTAGGCGCAACGGGACGGGAAGACCCCGTGAAGCTTTACTGTAGCTTA   2100
656         TGAAGACACCCGTTAGGCGCAACGGGACGGGAAGACCCCGTGAAGCTTTACTGTAGCTTA   2100
            ******************************  ****************************

683         ATATTGATCAAAACATCACCATGTAGAGAATAGGTAGGAGCAATTGATGCAAGTTCGCAA   2160
663         ATATTGATCAAAACACCACCATGTAGAGAATAGGTAGGAGCAATTGATGCAAGTTCGCAA   2160
20          ATATTGATCAAAACACCACCATGTAGAGAATAGGTAGGAGCAATTGATGCAAGTTCGCAA   2160
38          ATATTGATCAAAACACCACCATGTAGAGAATAGGTAGGAGCAATTGATGCAAGTTCGCAA   2160
649         ATATTGATCAAAACACCACCATGTAGAGAATAGGTAGGAGCAATTGATGCAAGTTCGCAA   2160
710         ATATTGATCAAAACACCACCATGTAGAGAATAGGTAGGAGCAATTGATGCAAGTTCGCAA   2160
719         ATATTGATCAAAACACCACCATGTAGAGAATAGGTAGGAGCAATTGATGCAAGTTCGCAA   2160
729         ATATTGATCAAAACACCACCATGTAGAGAATAGGTAGGAGCAATTGATGCAAGTTCGCAA   2160
735_        ATATTGATCAAAACACCACCATGTAGAGAATAGGTAGGAGCAATTGATGCAAGTTCGCAA   2160
1014        ATATTGATCAAAACACCACCATGTAGAGAATAGGTAGGAGCAATTGATGCAAGTTCGCAA   2160
```

```
122         ATATTGATCAAAACACCACCATGTAGAGAATAGGTAGGAGCAATTGATGCAAGTTCGCAA   2160
L43967      ATATTGATCAAAACACCACCATGTAGAGAATAGGTAGGAGCAATTGATGCAAGTTCGCAA   2160
720         ATATTGATCAAAACACCACCATGTAGAGAATAGGTAGGAGCAATTGATGCAAGTTCGCAA   2160
753         ATATTGATCAAAACACCACCATGTAGAGAATAGGTAGGAGCAATTGATGCAAGTTCGCAA   2160
1026        ATATTGATCAAAACACCACCATGTAGAGAATAGGTAGGAGCAATTGATGCAAGTTCGCAA   2160
47          ATATTGATCAAAACACCACCATGTAGAGAATAGGTAGGAGCAATTGATGCAAGTTCGCAA   2160
92          ATATTGATCAAAACACCACCATGTAGAGAATAGGTAGGAGCAATTGATGCAAGTTCGCAA   2160
600         ATATTGATCAAAACACCACCATGTAGAGAATAGGTAGGAGCAATTGATGCAAGTTCGCAA   2160
656         ATATTGATCAAAACACCACCATGTAGAGAATAGGTAGGAGCAATTGATGCAAGTTCGCAA   2160
            *************** ********************************************

683         GGATTTGTTGATGTGAAATGTGGAATACTACCCTTGGTGATGTTTTGTTCTAATTGGTAA   2220
663         GGATTTGTTGATGTGAAATGTGGAATACTACCCTTGGTTATGTTTTGTTCTAATTGGTAA   2220
20          GGATTTGTTGATGTGAAATGTGGAATACTACCCTTGGTTATGTTTTGTTCTAATTGGTAA   2220
38          GGATTTGTTGATGTGAAATGTGGAATACTACCCTTGGTTATGTTTTGTTCTAATTGGTAA   2220
649         GGATTTGTTGATGTGAAATGTGGAATACTACCCTTGGTTATGTTTTGTTCTAATTGGTAA   2220
710         GGATTTGTTGATGTGAAATGTGGAATACTACCCTTGGTTATGTTTTGTTCTAATTGGTAA   2220
719         GGATTTGTTGATGTGAAATGTGGAATACTACCCTTGGTTATGTTTTGTTCTAATTGGTAA   2220
729         GGATTTGTTGATGTGAAATGTGGAATACTACCCTTGGTTATGTTTTGTTCTAATTGGTAA   2220
735_        GGATTTGTTGATGTGAAATGTGGAATACTACCCTTGGTTATGTTTTGTTCTAATTGGTAA   2220
1014        GGATTTGTTGATGTGAAATGTGGAATACTACCCTTGGTTATGTTTTGTTCTAATTGGTAA   2220
122         GGATTTGTTGATGTGAAATGTGGAATACTACCCTTGGTTATGTTTTGTTCTAATTGGTAA   2220
L43967      GGATTTGTTGATGTGAAATGTGGAATACTACCCTTGGTTATGTTTTGTTCTAATTGGTAA   2220
720         GGATTTGTTGATGTGAAATGTGGAATACTACCCTTGGTTATGTTTTGTTCTAATTGGTAA   2220
753         GGATTTGTTGATGTGAAATGTGGAATACTACCCTTGGTTATGTTTTGTTCTAATTGGTAA   2220
1026        GGATTTGTTGATGTGAAATGTGGAATACTACCCTTGGTTATGTTTTGTTCTAATTGGTAA   2220
47          GGATTTGTTGATGTTAAATGTGGAATACTACCCTTGGTTATGTTTTGTTCTAATTGGTAA   2220
92          GGATTTGTTGATGTTAAATGTGGAATACTACCCTTGGTTATGTTTTGTTCTAATTGGTAA   2220
600         GGATTTGTTGATGTGAAATGTGGAATACTACCCTTGGTTATGTTTTGTTCTAATTGGTAA   2220
656         GGATTTGTTGATGTGAAATGTGGAATACTACCCTTGGTTATGTTTTGTTCTAATTGGTAA   2220
            ************** *********************** *********************

683         CTGTGATCCAGTTTCAAGACAGTGTTAGGTGGGCAGTTTGACTGGGGCGGTCGCCTCCTA   2280
663         CTGTGATCCAGTTTCAAGACAGTGTTAGGTGGGCAGTTTGACTGGGGCGGTCGCCTCCTA   2280
20          CTGTGATCCAGTTTCAAGACAGTGTTAGGTGGGCAGTTTGACTGGGGCGGTCGCCTCCTA   2280
38          CTGTGATCCAGTTTCAAGACAGTGTTAGGTGGGCAGTTTGACTGGGGCGGTCGCCTCCTA   2280
649         CTGTGATCCAGTTTCAAGACAGTGTTAGGTGGGCAGTTTGACTGGGGCGGTCGCCTCCTA   2280
710         CTGTGATCCAGTTTCAAGACAGTGTTAGGTGGGCAGTTTGACTGGGGCGGTCGCCTCCTA   2280
719         CTGTGATCCAGTTTCAAGACAGTGTTAGGTGGGCAGTTTGACTGGGGCGGTCGCCTCCTA   2280
729         CTGTGATCCAGTTTCAAGACAGTGTTAGGTGGGCAGTTTGACTGGGGCGGTCGCCTCCTA   2280
735_        CTGTGATCCAGTTTCAAGACAGTGTTAGGTGGGCAGTTTGACTGGGGCGGTCGCCTCCTA   2280
1014        CTGTGATCCAGTTTCAAGACAGTGTTAGGTGGGCAGTTTGACTGGGGCGGTCGCCTCCTA   2280
122         CTGTGATCCAGTTTCAAGACAGTGTTAGGTGGGCAGTTTGACTGGGGCGGTCGCCTCCTA   2280
L43967      CTGTGATCCAGTTTCAAGACAGTGTTAGGTGGGCAGTTTGACTGGGGCGGTCGCCTCCTA   2280
720         CTGTGATCCAGTTTCAAGACAGTGTTAGGTGGGCAGTTTGACTGGGGCGGTCGCCTCCTA   2280
753         CTGTGATCCAGTTTCAAGACAGTGTTAGGTGGGCAGTTTGACTGGGGCGGTCGCCTCCTA   2280
1026        CTGTGATCCAGTTTCAAGACAGTGTTAGGTGGGCAGTTTGACTGGGGCGGTCGCCTCCTA   2280
47          CTGTGATCCAGTTTCAAGACAGTGTTAGGTGGGCAGTTTGACTGGGGCGGTCGCCTCCTA   2280
92          CTGTGATCCAGTTTCAAGACAGTGTTAGGTGGGCAGTTTGACTGGGGCGGTCGCCTCCTA   2280
600         CTGTGATCCAGTTTCAAGACAGTGTTAGGTGGGCAGTTTGACTGGGGCGGTCGCCTCCTA   2280
656         CTGTGATCCAGTTTCAAGACAGTGTTAGGTGGGCAGTTTGACTGGGGCGGTCGCCTCCTA   2280
            ************************************************************

683         AAAGGTAACGGAGGCGCACAAAGGTACCTTCAGTACGGTTGGAAATCGTATTTAGAGTGT   2340
663         AAAGGTAACGGAGGCGCACAAAGGTACCTTCAGTACGGTTGGAAATCGTATTTAGAGTGT   2340
20          AAAGGTAACGGAGGCGCACAAAGGTACCTTCAGTACGGTTGGAAATCGTATTTAGAGTGT   2340
38          AAAGGTAACGGAGGCGCACAAAGGTACCTTCAGTACGGTTGGAAATCGTATTTAGAGTGT   2340
649         AAAGGTAACGGAGGCGCACAAAGGTACCTTCAGTACGGTTGGAAATCGTATTTAGAGTGT   2340
710         AAAGGTAACGGAGGCGCACAAAGGTACCTTCAGTACGGTTGGAAATCGTATTTAGAGTGT   2340
719         AAAGGTAACGGAGGCGCACAAAGGTACCTTCAGTACGGTTGGAAATCGTATTTAGAGTGT   2340
729         AAAGGTAACGGAGGCGCACAAAGGTACCTTCAGTACGGTTGGAAATCGTATTTAGAGTGT   2340
735_        AAAGGTAACGGAGGCGCACAAAGGTACCTTCAGTACGGTTGGAAATCGTATTTAGAGTGT   2340
1014        AAAGGTAACGGAGGCGCACAAAGGTACCTTCAGTACGGTTGGAAATCGTATTTAGAGTGT   2340
122         AAAGGTAACGGAGGCGCACAAAGGTACCTTCAGTACGGTTGGAAATCGTATTTAGAGTGT   2340
L43967      AAAGGTAACGGAGGCGCACAAAGGTACCTTCAGTACGGTTGGAAATCGTATTTAGAGTGT   2340
720         AAAGGTAACGGAGGCGCACAAAGGTACCTTCAGTACGGTTGGAAATCGTATTTAGAGTGT   2340
753         AAAGGTAACGGAGGCGCACAAAGGTACCTTCAGTACGGTTGGAAATCGTATTTAGAGTGT   2340
1026        AAAGGTAACGGAGGCGCACAAAGGTACCTTCAGTACGGTTGGAAATCGTATTTAGAGTGT   2340
47          AAAGGTAACGGAGGCGCACAAAGGTACCTTCAGTACGGTTGGAAATCGTATTTAGAGTGT   2340
92          AAAGGTAACGGAGGCGCACAAAGGTACCTTCAGTACGGTTGGAAATCGTATTTAGAGTGT   2340
600         AAAGGTAACGGAGGCGCACAAAGGTACCTTCAGTACGGTTGGAAATCGTATTTAGAGTGT   2340
656         AAAGGTAACGGAGGCGCACAAAGGTACCTTCAGTACGGTTGGAAATCGTATTTAGAGTGT   2340
            ************************************************************

683         AATGGTATAAGGGTGCTTGACTGTGAGACTTACAGGTCGAACAGGTGAGAAATCAGGTCA   2400
663         AATGGTATAAGGGTGCTTGACTGTGAGACTTACAGGTCGAACAGGTGAGAAATCAGGTCA   2400
20          AATGGTATAAGGGTGCTTGACTGTGAGACTTACAGGTCGAACAGGTGAGAAATCAGGTCA   2400
38          AATGGTATAAGGGTGCTTGACTGTGAGACTTACAGGTCGAACAGGTGAGAAATCAGGTCA   2400
649         AATGGTATAAGGGTGCTTGACTGTGAGACTTACAGGTCGAACAGGTGAGAAATCAGGTCA   2400
710         AATGGTATAAGGGTGCTTGACTGTGAGACTTACAGGTCGAACAGGTGAGAAATCAGGTCA   2400
719         AATGGTATAAGGGTGCTTGACTGTGAGACTTACAGGTCGAACAGGTGAGAAATCAGGTCA   2400
729         AATGGTATAAGGGTGCTTGACTGTGAGACTTACAGGTCGAACAGGTGAGAAATCAGGTCA   2400
735_        AATGGTATAAGGGTGCTTGACTGTGAGACTTACAGGTCGAACAGGTGAGAAATCAGGTCA   2400
```

```
1014      AATGGTATAAGGGTGCTTGACTGTGAGACTTACAGGTCGAACAGGTGAGAAATCAGGTCA   2400
122       AATGGTATAAGGGTGCTTGACTGTGAGACTTACAGGTCGAACAGGTGAGAAATCAGGTCA   2400
L43967    AATGGTATAAGGGTGCTTGACTGTGAGACTTACAGGTCGAACAGGTGAGAAATCAGGTCA   2400
720       AATGGTATAAGGGTGCTTGACTGTGAGACTTACAGGTCGAACAGGTGAGAAATCAGGTCA   2400
753       AATGGTATAAGGGTGCTTGACTGTGAGACTTACAGGTCGAACAGGTGAGAAATCAGGTCA   2400
1026      AATGGTATAAGGGTGCTTGACTGTGAGACTTACAGGTCGAACAGGTGAGAAATCAGGTCA   2400
47        AATGGTATAAGGGTGCTTGACTGTGAGACTTACAGGTCGAACAGGTGAGAAATCAGGTCA   2400
92        AATGGTATAAGGGTGCTTGACTGTGAGACTTACAGGTCGAACAGGTGAGAAATCAGGTCA   2400
600       AATGGTATAAGGGTGCTTGACTGTGAGACTTACAGGTCGAACAGGTGAGAAATCAGGTCA   2400
656       AATGGTATAAGGGTGCTTGACTGTGAGACTTACAGGTCGAACAGGTGAGAAATCAGGTCA   2400
          ************************************************************

683       TAGTGATCCGGTGGTTCAGTATGGAATGGCCATCGCTCAACGGATAAAAGCTACTCCGGG   2460
663       TAGTGATCCGGTGGTTCAGTATGGAATGGCCATCGCTCAACGGATAAAAGCTACTCCGGG   2460
20        TAGTGATCCGGTGGTTCAGTATGGAATGGCCATCGCTCAACGGATAAAAGCTACTCCGGG   2460
38        TAGTGATCCGGTGGTTCAGTATGGAATGGCCATCGCTCAACGGATAAAAGCTACTCCGGG   2460
649       TAGTGATCCGGTGGTTCAGTATGGAATGGCCATCGCTCAACGGATAAAAGCTACTCCGGG   2460
710       TAGTGATCCGGTGGTTCAGTATGGAATGGCCATCGCTCAACGGATAAAAGCTACTCCGGG   2460
719       TAGTGATCCGGTGGTTCAGTATGGAATGGCCATCGCTCAACGGATAAAAGCTACTCCGGG   2460
729       TAGTGATCCGGTGGTTCAGTATGGAATGGCCATCGCTCAACGGATAAAAGCTACTCCGGG   2460
735_      TAGTGATCCGGTGGTTCAGTATGGAATGGCCATCGCTCAACGGATAAAAGCTACTCCGGG   2460
1014      TAGTGATCCGGTGGTTCAGTATGGAATGGCCATCGCTCAACGGATAAAAGCTACTCCGGG   2460
122       TAGTGATCCGGTGGTTCAGTATGGAATGGCCATCGCTCAACGGATAAAAGCTACTCCGGG   2460
L43967    TAGTGATCCGGTGGTTCAGTATGGAATGGCCATCGCTCAACGGATAAAAGCTACTCCGGG   2460
720       TAGTGATCCGGTGGTTCAGTATGGAATGGCCATCGCTCAACGGATAAAAGCTACTCCGGG   2460
753       TAGTGATCCGGTGGTTCAGTATGGAATGGCCATCGCTCAACGGATAAAAGCTACTCCGGG   2460
1026      TAGTGATCCGGTGGTTCAGTATGGAATGGCCATCGCTCAACGGATAAAAGCTACTCCGGG   2460
47        TAGTGATCCGGTGGTTCAGTATGGAATGGCCATCGCTCAACGGATAAAAGCTACTCCGGG   2460
92        TAGTGATCCGGTGGTTCAGTATGGAATGGCCATCGCTCAACGGATAAAAGCTACTCCGGG   2460
600       TAGTGATCCGGTGGTTCAGTATGGAATGGCCATCGCTCAACGGATAAAAGCTACTCCGGG   2460
656       TAGTGATCCGGTGGTTCAGTATGGAATGGCCATCGCTCAACGGATAAAAGCTACTCCGGG   2460
          ************************************************************

683       GATAACAGGCTGATACTGCCCAAGAGTTCATATCGACGGCAGTGTTTGGCACCTCGATGT   2520
663       GATAACAGGCTGATACTGCCCAAGAGTTCATATCGACGGCAGTGTTTGGCACCTCGATGT   2520
20        GATAACAGGCTGATACTGCCCAAGAGTTCATATCGACGGCAGTGTTTGGCACCTCGATGT   2520
38        GATAACAGGCTGATACTGCCCAAGAGTTCATATCGACGGCAGTGTTTGGCACCTCGATGT   2520
649       GATAACAGGCTGATACTGCCCAAGAGTTCATATCGACGGCAGTGTTTGGCACCTCGATGT   2520
710       GATAACAGGCTGATACTGCCCAAGAGTTCATATCGACGGCAGTGTTTGGCACCTCGATGT   2520
719       GATAACAGGCTGATACTGCCCAAGAGTTCATATCGACGGCAGTGTTTGGCACCTCGATGT   2520
729       GATAACAGGCTGATACTGCCCAAGAGTTCATATCGACGGCAGTGTTTGGCACCTCGATGT   2520
735_      GATAACAGGCTGATACTGCCCAAGAGTTCATATCGACGGCAGTGTTTGGCACCTCGATGT   2520
1014      GATAACAGGCTGATACTGCCCAAGAGTTCATATCGACGGCAGTGTTTGGCACCTCGATGT   2520
122       GATAACAGGCTGATACTGCCCAAGAGTTCATATCGACGGCAGTGTTTGGCACCTCGATGT   2520
L43967    GATAACAGGCTGATACTGCCCAAGAGTTCATATCGACGGCAGTGTTTGGCACCTCGATGT   2520
720       GATAACAGGCTGATACTGCCCAAGAGTTCATATCGACGGCAGTGTTTGGCACCTCGATGT   2520
753       GATAACAGGCTGATACTGCCCAAGAGTTCATATCGACGGCAGTGTTTGGCACCTCGATGT   2520
1026      GATAACAGGCTGATACTGCCCAAGAGTTCATATCGACGGCAGTGTTTGGCACCTCGATGT   2520
47        GATAACAGGCTGATACTGCCCAAGAGTTCATATCGACGGCAGTGTTTGGCACCTCGATGT   2520
92        GATAACAGGCTGATACTGCCCAAGAGTTCATATCGACGGCAGTGTTTGGCACCTCGATGT   2520
600       GATAACAGGCTGATACTGCCCAAGAGTTCATATCGACGGCAGTGTTTGGCACCTCGATGT   2520
656       GATAACAGGCTGATACTGCCCAAGAGTTCATATCGACGGCAGTGTTTGGCACCTCGATGT   2520
          ************************************************************

683       CGACTCATCTCATCCTCGAGCTGAAGCAGGTTCGAAGGGTTCGGCTGTTCGCCGATTAAA   2580
663       CGACTCATCTCATCCTCGAGCTGAAGTAGGTTCGAAGGGTTCGGCTGTTCGCCGATTAAA   2580
20        CGACTCATCTCATCCTCGAGCTGAAGCAGGTTCGAAGGGTTCGGCTGTTCGCCGATTAAA   2580
38        CGACTCATCTCATCCTCGAGCTGAAGCAGGTTCGAAGGGTTCGGCTGTTCGCCGATTAAA   2580
649       CGACTCATCTCATCCTCGAGCTGAAGCAGGTTCGAAGGGTTCGGCTGTTCGCCGATTAAA   2580
710       CGACTCATCTCATCCTCGAGCTGAAGCAGGTTCGAAGGGTTCGGCTGTTCGCCGATTAAA   2580
719       CGACTCATCTCATCCTCGAGCTGAAGCAGGTTCGAAGGGTTCGGCTGTTCGCCGATTAAA   2580
729       CGACTCATCTCATCCTCGAGCTGAAGCAGGTTCGAAGGGTTCGGCTGTTCGCCGATTAAA   2580
735_      CGACTCATCTCATCCTCGAGCTGAAGCAGGTTCGAAGGGTTCGGCTGTTCGCCGATTAAA   2580
1014      CGACTCATCTCATCCTCGAGCTGAAGCAGGTTCGAAGGGTTCGGCTGTTCGCCGATTAAA   2580
122       CGACTCATCTCATCCTCGAGCTGAAGCAGGTTCGAAGGGTTCGGCTGTTCGCCGATTAAA   2580
L43967    CGACTCATCTCATCCTCGAGCTGAAGCAGGTTCGAAGGGTTCGGCTGTTCGCCGATTAAA   2580
720       CGACTCATCTCATCCTCGAGCTGAAGCAGGTTCGAAGGGTTCGGCTGTTCGCCGATTAAA   2580
753       CGACTCATCTCATCCTCGAGCTGAAGCAGGTTCGAAGGGTTCGGCTGTTCGCCGATTAAA   2580
1026      CGACTCATCTCATCCTCGAGCTGAAGCAGGTTCGAAGGGTTCGGCTGTTCGCCGATTAAA   2580
47        CGACTCATCTCATCCTCGAGCTGAAGCAGGTTCGAAGGGTTCGGCTGTTCGCCGATTAAA   2580
92        CGACTCATCTCATCCTCGAGCTGAAGCAGGTTCGAAGGGTTCGGCTGTTCGCCGATTAAA   2580
600       CGACTCATCTCATCCTCGAGCTGAAGCAGGTTCGAAGGGTTCGGCTGTTCGCCGATTAAA   2580
656       CGACTCATCTCATCCTCGAGCTGAAGCAGGTTCGAAGGGTTCGGCTGTTCGCCGATTAAA   2580
          ************************** *********************************

683       GAGATACGTGAGTTGGGTTCAAACCGTCGTGAGACAGGTTGGTCCCTATCTATTGTGCCC   2640
663       GAGATACGTGAGTTGGGTTCAAACCGTCGTGAGACAGGTTGGTCCCTATCTATTGTGCCC   2640
20        GAGATACGTGAGTTGGGTTCAAACCGTCGTGAGACAGGTTGGTCCCTATCTATTGTGCCC   2640
38        GAGATACGTGAGTTGGGTTCAAACCGTCGTGAGACAGGTTGGTCCCTATCTATTGTGCCC   2640
649       GAGATACGTGAGTTGGGTTCAAACCGTCGTGAGACAGGTTGGTCCCTATCTATTGTGCCC   2640
710       GAGATACGTGAGTTGGGTTCAAACCGTCGTGAGACAGGTTGGTCCCTATCTATTGTGCCC   2640
719       GAGATACGTGAGTTGGGTTCAAACCGTCGTGAGACAGGTTGGTCCCTATCTATTGTGCCC   2640
729       GAGATACGTGAGTTGGGTTCAAACCGTCGTGAGACAGGTTGGTCCCTATCTATTGTGCCC   2640
```

```
735_        GAGATACGTGAGTTGGGTTCAAACCGTCGTGAGACAGGTTGGTCCCTATCTATTGTGCCC   2640
1014        GAGATACGTGAGTTGGGTTCAAACCGTCGTGAGACAGGTTGGTCCCTATCTATTGTGCCC   2640
122         GAGATACGTGAGTTGGGTTCAAACCGTCGTGAGACAGGTTGGTCCCTATCTATTGTGCCC   2640
L43967      GAGATACGTGAGTTGGGTTCAAACCGTCGTGAGACAGGTTGGTCCCTATCTATTGTGCCC   2640
720         GAGATACGTGAGTTGGGTTCAAACCGTCGTGAGACAGGTTGGTCCCTATCTATTGTGCCC   2640
753         GAGATACGTGAGTTGGGTTCAAACCGTCGTGAGACAGGTTGGTCCCTATCTATTGTGCCC   2640
1026        GAGATACGTGAGTTGGGTTCAAACCGTCGTGAGACAGGTTGGTCCCTATCTATTGTGCCC   2640
47          GAGATACGTGAGTTGGGTTCAAACCGTCGTGAGACAGGTTGGTCCCTATCTATTGTGCCC   2640
92          GAGATACGTGAGTTGGGTTCAAACCGTCGTGAGACAGGTTGGTCCCTATCTATTGTGCCC   2640
600         GAGATACGTGAGTTGGGTTCAAACCGTCGTGAGACAGGTTGGTCCCTATCTATTGTGCCC   2640
656         GAGATACGTGAGTTGGGTTCAAACCGTCGTGAGACAGGTTGGTCCCTATCTATTGTGCCC   2640
            ************************************************************

683         ACAGGAAGATTGAAGAGCTTTGCTTCTAGTACGAGAGGACCGGAGCGAGGACACCGCTTA   2700
663         ACAGGAAGATTGAAGAGCTTTGCTTCTAGTACGAGAGGACCGGAGCGAGGACACCGCTTA   2700
20          ACAGGAAGATTGAAGAGCTTTGCTTCTAGTACGAGAGGACCGGAGCGAGGACACCGCTTA   2700
38          ACAGGAAGATTGAAGAGCTTTGCTTCTAGTACGAGAGGACCGGAGCGAGGACACCGCTTA   2700
649         ACAGGAAGATTGAAGAGCTTTGCTTCTAGTACGAGAGGACCGGAGCGAGGACACCGCTTA   2700
710         ACAGGAAGATTGAAGAGCTTTGCTTCTAGTACGAGAGGACCGGAGCGAGGACACCGCTTA   2700
719         ACAGGAAGATTGAAGAGCTTTGCTTCTAGTACGAGAGGACCGGAGCGAGGACACCGCTTA   2700
729         ACAGGAAGATTGAAGAGCTTTGCTTCTAGTACGAGAGGACCGGAGCGAGGACACCGCTTA   2700
735_        ACAGGAAGATTGAAGAGCTTTGCTTCTAGTACGAGAGGACCGGAGCGAGGACACCGCTTA   2700
1014        ACAGGAAGATTGAAGAGCTTTGCTTCTAGTACGAGAGGACCGGAGCGAGGACACCGCTTA   2700
122         ACAGGAAGATTGAAGAGCTTTGCTTCTAGTACGAGAGGACCGGAGCGAGGACACCGCTTA   2700
L43967      ACAGGAAGATTGAAGAGCTTTGCTTCTAGTACGAGAGGACCGGAGCGAGGACACCGCTTA   2700
720         ACAGGAAGATTGAAGAGCTTTGCTTCTAGTACGAGAGGACCGGAGCGAGGACACCGCTTA   2700
753         ACAGGAAGATTGAAGAGCTTTGCTTCTAGTACGAGAGGACCGGAGCGAGGACACCGCTTA   2700
1026        ACAGGAAGATTGAAGAGCTTTGCTTCTAGTACGAGAGGACCGGAGCGAGGACACCGCTTA   2700
47          ACAGGAAGATTGAAGAGCTTTGCTTCTAGTACGAGAGGACCGGAGCGAGGACACCGCTTA   2700
92          ACAGGAAGATTGAAGAGCTTTGCTTCTAGTACGAGAGGACCGGAGCGAGGACACCGCTTA   2700
600         ACAGGAAGATTGAAGAGCTTTGCTTCTAGTACGAGAGGACCGGAGCGAGGACACCGCTTA   2700
656         ACAGGAAGATTGAAGAGCTTTGCTTCTAGTACGAGAGGACCGGAGCGAGGACACCGCTTA   2700
            ************************************************************

683         TGCTCCAGTTGTAGCGCCAGCTGCACCGCTGGGT      2734
663         TGCTCCAGTTGTAGCGCCAGCTGCACCGCTGGGT      2734
20          TGCTCCAGTTGTAGCGCCAGCTGCACCGCTGGGT      2734
38          TGCTCCAGTTGTAGCGCCAGCTGCACCGCTGGGT      2734
649         TGCTCCAGTTGTAGCGCCAGCTGCACCGCTGGGT      2734
710         TGCTCCAGTTGTAGCGCCAGCTGCACCGCTGGGT      2734
719         TGCTCCAGTTGTAGCGCCAGCTGCACCGCTGGGT      2734
729         TGCTCCAGTTGTAGCGCCAGCTGCACCGCTGGGT      2734
735_        TGCTCCAGTTGTAGCGCCAGCTGCACCGCTGGGT      2734
1014        TGCTCCAGTTGTAGCGCCAGCTGCACCGCTGGGT      2734
122         TGCTCCAGTTGTAGCGCCAGCTGCACCGCTGGGT      2734
L43967      TGCTCCAGTTGTAGCGCCAGCTGCACCGCTGGGT      2734
720         TGCTCCAGTTGTAGCGCCAGCTGCACCGCTGGGT      2734
753         TGCTCCAGTTGTAGCGCCAGCTGCACCGCTGGGT      2734
1026        TGCTCCAGTTGTAGCGCCAGCTGCACCGCTGGGT      2734
47          TGCTCCAGTTGTAGCGCCAGCTGCACCGCTGGGT      2734
92          TGCTCCAGTTGTAGCGCCAGCTGCACCGCTGGGT      2734
600         TGCTCCAGTTGTAGCGCCAGCTGCACCGCTGGGT      2734
656         TGCTCCAGTTGTAGCGCCAGCTGCACCGCTGGGT      2734
            **********************************
```

***parC* gene alignment**

```
719         ATGGATCAAAAAAACAACAACCTCTTTCAAAAGGCAATTGAAGAAGTCTTTGCAGTTAGC   60
20          ATGGATCAAAAAAACAACAACCTCTTTCAAAAGGCAATTGAAGAAGTCTTTGCAGTTAGC   60
735         ATGGATCAAAAAAACAACAACCTCTTTCAAAAGGCAATTGAAGAAGTCTTTGCAGTTAGC   60
649         ATGGATCAAAAAAACAACAACCTCTTTCAAAAGGCAATTGAAGAAGTCTTTGCAGTTAGC   60
710         ATGGATCAAAAAAACAACAACCTCTTTCAAAAGGCAATTGAAGAAGTCTTTGCAGTTAGC   60
729         ATGGATCAAAAAAACAACAACCTCTTTCAAAAGGCAATTGAAGAAGTCTTTGCAGTTAGC   60
38          ATGGATCAAAAAAACAACAACCTCTTTCAAAAGGCAATTGAAGAAGTCTTTGCAGTTAGC   60
600         ATGGATCAAAAAAACAACAACCTCTTTCAAAAGGCAATTGAAGAAGTCTTTGCAGTTAGC   60
122         ATGGATCAAAAAAACAACAACCTCTTTCAAAAGGCAATTGAAGAAGTCTTTGCAGTTAGC   60
683         ATGGATCAAAAAAACAACAACCTCTTTCAAAAGGCAATTGAAGAAGTCTTTGCAGTTAGC   60
1026        ATGGATCAAAAAAACAACAACCTCTTTCAAAAGGCAATTGAAGAAGTCTTTGCAGTTAGC   60
753         ATGGATCAAAAAAACAACAACCTCTTTCAAAAGGCAATTGAAGAAGTCTTTGCAGTTAGC   60
720         ATGGATCAAAAAAACAACAACCTCTTTCAAAAGGCAATTGAAGAAGTCTTTGCAGTTAGC   60
47          ATGGATCAAAAAAACAACAACCTCTTTCAAAAGGCAATTGAAGAAGTCTTTGCAGTTAGC   60
663         ATGGATCAAAAAAACAACAACCTCTTTCAAAAGGCAATTGAAGAAGTCTTTGCAGTTAGC   60
1014        ATGGATCAAAAAAACAACAACCTCTTTCAAAAGGCAATTGAAGAAGTCTTTGCAGTTAGC   60
L43967      ATGGATCAAAAAAACAACAACCTCTTTCAAAAGGCAATTGAAGAAGTCTTTGCAGTTAGC   60
656         ATGGATCAAAAAAACAACAACCTCTTTCAAAAGGCAATTGAAGAAGTCTTTGCAGTTAGC   60
            ************************************************************

719         TTTAGTAAGTATGCTAAATACATCATCCAAGATAGAGCTTTACCTGATCTAAGAGATGGG   120
20          TTTAGTAAGTATGCTAAATACATCATCCAAGATAGAGCTTTACCTGATCTAAGAGATGGG   120
735         TTTAGTAAGTATGCTAAATACATCATCCAAGATAGAGCTTTACCTGATCTAAGAGATGGG   120
649         TTTAGTAAGTATGCTAAATACATCATCCAAGATAGAGCTTTACCTGATCTAAGAGATGGG   120
710         TTTAGTAAGTATGCTAAATACATCATCCAAGATAGAGCTTTACCTGATCTAAGAGATGGG   120
729         TTTAGTAAGTATGCTAAATACATCATCCAAGATAGAGCTTTACCTGATCTAAGAGATGGG   120
38          TTTAGTAAGTATGCTAAATACATCATCCAAGATAGAGCTTTACCTGATCTAAGAGATGGG   120
600         TTTAGTAAGTATGCTAAATACATCATCCAAGATAGAGCTTTACCTGATCTAAGAGATGGG   120
122         TTTAGTAAGTATGCTAAATACATCATCCAAGATAGAGCTTTACCTGATCTAAGAGATGGG   120
683         TTTAGTAAGTATGCTAAATACATCATCCAAGATAGAGCTTTACCTGATCTAAGAGATGGG   120
1026        TTTAGTAAGTATGCTAAATACATCATCCAAGATAGAGCTTTACCTGATCTAAGAGATGGG   120
753         TTTAGTAAGTATGCTAAATACATCATCCAAGATAGAGCTTTACCTGATCTAAGAGATGGG   120
720         TTTAGTAAGTATGCTAAATACATCATCCAAGATAGAGCTTTACCTGATCTAAGAGATGGG   120
47          TTTAGTAAGTATGCTAAATACATCATCCAAGATAGAGCTTTACCTGATCTAAGAGATGGG   120
663         TTTAGTAAGTATGCTAAATACATCATCCAAGATAGAGCTTTACCTGATCTAAGAGATGGG   120
1014        TTTAGTAAGTATGCTAAATACATCATCCAAGATAGAGCTTTACCTGATCTAAGAGATGGG   120
L43967      TTTAGTAAGTATGCTAAATACATCATCCAAGATAGAGCTTTACCTGATCTAAGAGATGGG   120
656         TTTAGTAAGTATGCTAAATACATCATCCAAGATAGAGCTTTACCTGATCTAAGAGATGGG   120
            ************************************************************

719         TTAAAACCAGTACAAAGACGGATCTTATATGGGATGTTTCAAATGGGCTTAAAACCCACC   180
20          TTAAAACCAGTACAAAGACGGATCTTATATGGGATGTTTCAAATGGGCTTAAAACCCACC   180
735         TTAAAACCAGTACAAAGACGGATCTTATATGGGATGTTTCAAATGGGCTTAAAACCCACC   180
649         TTAAAACCAGTACAAAGACGGATCTTATATGGGATGTTTCAAATGGGCTTAAAACCCACC   180
710         TTAAAACCAGTACAAAGACGGATCTTATATGGGATGTTTCAAATGGGCTTAAAACCCACC   180
729         TTAAAACCAGTACAAAGACGGATCTTATATGGGATGTTTCAAATGGGCTTAAAACCCACC   180
38          TTAAAACCAGTACAAAGACGGATCTTATATGGGATGTTTCAAATGGGCTTAAAACCCACC   180
600         TTAAAACCAGTACAAAGACGGATCTTATATGGGATGTTTCAAATGGGCTTAAAACCCACC   180
122         TTAAAACCAGTACAAAGACGGATCTTATATGGGATGTTTCAAATGGGCTTAAAACCCACC   180
683         TTAAAACCAGTACAAAGACGGATCTTATATGGGATGTTTCAAATGGGCTTAAAACCCACC   180
1026        TTAAAACCAGTACAAAGACGGATCTTATATGGGATGTTTCAAATGGGCTTAAAACCCACC   180
753         TTAAAACCAGTACAAAGACGGATCTTATATGGGATGTTTCAAATGGGCTTAAAACCCACC   180
720         TTAAAACCAGTACAAAGACGGATCTTATATGGGATGTTTCAAATGGGCTTAAAACCCACC   180
47          TTAAAACCAGTACAAAGACGGATCTTATATGGGATGTTTCAAATGGGCTTAAAACCCACC   180
663         TTAAAACCAGTACAAAGACGGATCTTATATGGGATGTTTCAAATGGGTTTAAAACCCACC   180
1014        TTAAAACCAGTACAAAGACGGATCTTATATGGGATGTTTCAAATGGGCTTAAAACCCACC   180
L43967      TTAAAACCAGTACAAAGACGGATCTTATATGGGATGTTTCAAATGGGCTTAAAACCCACC   180
656         TTAAAACCAGTACAAAGACGGATCTTATATGGGATGTTTCAAATGGGCTTAAAACCCACC   180
            *********************************************** ************

719         ACTCCCTATAAAAAATCAGCCCGTGCTGTTGGGGAGATCATGGGGAAATACCACCCCCAT   240
20          ACTCCCTATAAAAAATCAGCCCGTGCTGTTGGGGAGATCATGGGGAAATACCACCCCCAT   240
735         ACTCCCTATAAAAAATCAGCCCGTGCTGTTGGGGAGATCATGGGGAAATACCACCCCCAT   240
649         ACTCCCTATAAAAAATCAGCCCGTGCTGTTGGGGAGATCATGGGGAAATACCACCCCCAT   240
710         ACTCCCTATAAAAAATCAGCCCGTGCTGTTGGGGAGATCATGGGGAAATACCACCCCCAT   240
729         ACTCCCTATAAAAAATCAGCCCGTGCTGTTGGGGAGATCATGGGGAAATACCACCCCCAT   240
38          ACTCCCTATAAAAAATCAGCCCGTGCTGTTGGGGAGATCATGGGGAAATACCACCCCCAT   240
600         ACTCCCTATAAAAAATCAGCCCGTGCTGTTGGGGAGATCATGGGGAAATACCACCCCCAT   240
122         ACTCCCTATAAAAAATCAGCCCGTGCTGTTGGGGAGATCATGGGGAAATACCACCCCCAT   240
683         ACTCCCTATAAAAAATCAGCCCGTGCTGTTGGGGAGATCATGGGGAAATACCACCCCCAT   240
1026        ACTCCCTATAAAAAATCAGCCCGTGCTGTTGGGGAGATCATGGGGAAATACCACCCCCAT   240
753         ACTCCCTATAAAAAATCAGCCCGTGCTGTTGGGGAGATCATGGGGAAATACCACCCCCAT   240
720         ACTCCCTATAAAAAATCAGCCCGTGCTGTTGGGGAGATCATGGGGAAATACCACCCCCAT   240
47          ACTCCCTATAAAAAATCAGCCCGTGCTGTTGGGGAGATCATGGGGAAATACCACCCCCAT   240
663         ACTCCCTATAAAAAATCAGCCCGTGCTGTTGGGGAGATCATGGGGAAATACCACCCCCAT   240
1014        ACTCCCTATAAAAAATCAGCCCGTGCTGTTGGGGAGATCATGGGGAAATACCACCCCCAT   240
L43967      ACTCCCTATAAAAAATCAGCCCGTGCTGTTGGGGAGATCATGGGGAAATACCACCCCCAT   240
656         ACTCCCTATAAAAAATCAGCCCGTGCTGTTGGGGAGATCATGGGGAAATACCACCCCCAT   240
            ************************************************************

719         GGTGATATTTCCATTTATGATGCAATTATCAGAATGTCCCAAAGCTGAAAGAACAACTGA   300
```

```
20        GGTGATATTTCCATTTATGATGCAATTATCAGAATGTCCCAAAGCTGAAAGAACAACTGA   300
735       GGTGATATTTCCATTTATGATGCAATTATCAGAATGTCCCAAAGCTGAAAGAACAACTGA   300
649       GGTGATATTTCCATTTATGATGCAATTATCAGAATGTCCCAAAGCTGAAAGAACAACTGA   300
710       GGTGATATTTCCATTTATGATGCAATTATCAGAATGTCCCAAAGCTGAAAGAACAACTGA   300
729       GGTGATATTTCCATTTATGATGCAATTATCAGAATGTCCCAAAGCTGAAAGAACAACTGA   300
38        GGTGATAGTTCCATTTATGATGCAATTATCAGAATGTCCCAAAGCTGAAAGAACAACTGA   300
600       GGTGATAGTTCCATTTATGATGCAATTATCAGAATGTCCCAAAGCTGAAAGAACAACTGA   300
122       GGTGATAGTTCCATTTATGATGCAATTATCAGAATGTCCCAAAGCTGAAAGAACAACTGA   300
683       GGTGATATTTCCATTTATGATGCAATTATCAGAATGTCCCAAAGCTGAAAGAACAACTGA   300
1026      GGTGATATTTCCATTTATGATGCAATTATCAGAATGTCCCAAAGCTGAAAGAACAACTGA   300
753       GGTGATAGTTCCATTTATGATGCAATTATCAGAATGTCCCAAAGCTGAAAGAACAACTGA   300
720       GGTGATAGTTCCATTTATGATGCAATTATCAGAATGTCCCAAAGCTGAAAGAACAACTGA   300
47        GGTGATAGTTCCATTTATGATGCAATTATCAGAATGTCCCAAAGCTGAAAGAACAACTGA   300
663       GGTGATAGTTCCATTTATGATGCAATTATCAGAATGTCCCAAAGCTGAAAGAACAACTGA   300
1014      GGTGATATTTCCATTTATGATGCAATTATCAGAATGTCCCAAAGCTGAAAGAACAACTGA   300
L43967    GGTGATAGTTCCATTTATGATGCAATTATCAGAATGTCCCAAAGCTGAAAGAACAACTGA   300
656       GGTGATAGTTCCATTTATGATGCAATTATCAGAATGTCCCAAAGCTGAAAGAACAACTGA   300
          ******* ****************************************************

719       ACAACTGTTTCTATCCATGGTAACAATGGTTCAGTGGATGGGGATAATGCTGCAGCAATG   360
20        ACAACTGTTTCTATCCATGGTAACAATGGTTCAGTGGATGGGGATAATGCTGCAGCAATG   360
735       ACAACTGTTTCTATCCATGGTAACAATGGTTCAGTGGATGGGGATAATGCTGCAGCAATG   360
649       ACAACTGTTTCTATCCATGGTAACAATGGTTCAGTGGATGGGGATAATGCTGCAGCAATG   360
710       ACAACTGTTTCTATCCATGGTAACAATGGTTCAGTGGATGGGGATAATGCTGCAGCAATG   360
729       ACAACTGTTTCTATCCATGGTAACAATGGTTCAGTGGATGGGGATAATGCTGCAGCAATG   360
38        ACAACTGTTTCTATCCATGGTAACAATGGTTCAGTGGATGGGGATAATGCTGCAGCAATG   360
600       ACAACTGTTTCTATCCATGGTAACAATGGTTCAGTGGATGGGGATAATGCTGCAGCAATG   360
122       ACAACTGTTTCTATCCATGGTAACAATGGTTCAGTGGATGGGGATAATGCTGCAGCAATG   360
683       ACAACTGTTTCTATCCATGGTAACAATGGTTCAGTGGATGGGGATAATGCTGCAGCAATG   360
1026      ACAACTGTTTCTATCCATGGTAACAATGGTTCAGTGGATGGGGATAATGCTGCAGCAATG   360
753       ACAACTGTTTCTATCCATGGTAACAATGGTTCAGTGGATGGGGATAATGCTGCAGCAATG   360
720       ACAACTGTTTCTATCCATGGTAACAATGGTTCAGTGGATGGGGATAATGCTGCAGCAATG   360
47        ACAACTGTTTCTATCCATGGTAACAATGGTTCAGTGGATGGGGATAATGCTGCAGCAATG   360
663       ACAACTGTTTCTATCCATGGTAACAATGGTTCAGTGGATGGGGATAATGCTGCAGCAATG   360
1014      ACAACTGTTTCTATCCATGGTAACAATGGTTCAGTGGATGGGGATAATGCTGCAGCAATG   360
L43967    ACAACTGTTTCTATCCATGGTAACAATGGTTCAGTGGATGGGGATAATGCTGCAGCAATG   360
656       ACAACTGTTTCTATCCATGGTAACAATGGTTCAGTGGATGGGGATAATGCTGCAGCAATG   360
          ************************************************************

719       CGTTACACAGAAACCCGCTTAAGCTTGTATGGATTTGAACTATTAAAAGACATTGATAAA   420
20        CGTTACACAGAAACCCGCTTAAGCTTGTATGGATTTGAACTATTAAAAGACATTGATAAA   420
735       CGTTACACAGAAACCCGCTTAAGCTTGTATGGATTTGAACTATTAAAAGACATTGATAAA   420
649       CGTTACACAGAAACCCGCTTAAGCTTGTATGGATTTGAACTATTAAAAGACATTGATAAA   420
710       CGTTACACAGAAACCCGCTTAAGCTTGTATGGATTTGAACTATTAAAAGACATTGATAAA   420
729       CGTTACACAGAAACCCGCTTAAGCTTGTATGGATTTGAACTATTAAAAGACATTGATAAA   420
38        CGTTACACAGAAACCCGCTTAAGCTTGTATGGATTTGAACTATTAAAAGACATTGATAAA   420
600       CGTTACACAGAAACCCGCTTAAGCTTGTATGGATTTGAACTATTAAAAGACATTGATAAA   420
122       CGTTACACAGAAACCCGCTTAAGCTTGTATGGATTTGAACTATTAAAAGACATTGATAAA   420
683       CGTTACACAGAAACCCGCTTAAGCTTGTATGGATTTGAACTATTAAAAGACATTGATAAA   420
1026      CGTTACACAGAAACCCGCTTAAGCTTGTATGGATTTGAACTATTAAAAGACATTGATAAA   420
753       CGTTACACAGAAACCCGCTTAAGCTTGTATGGATTTGAACTATTAAAAGACATTGATAAA   420
720       CGTTACACAGAAACCCGCTTAAGCTTGTATGGATTTGAACTATTAAAAGACATTGATAAA   420
47        CGTTATACAGAAACCCGCTTAAGCTTGTATGGATTTGAACTATTAAAAGACATTGATAAA   420
663       CGTTACACAGAAACCCGCTTAAGCTTGTATGGATTTGAACTATTAAAAGACATTGATAAA   420
1014      CGTTACACAGAAACCCGCTTAAGCTTGTATGGATTTGAACTATTAAAAGACATTGATAAA   420
L43967    CGTTACACAGAAACCCGCTTAAGCTTGTATGGATTTGAACTATTAAAAGACATTGATAAA   420
656       CGTTACACAGAAACCCGCTTAAGCTTGTATGGATTTGAACTATTAAAAGACATTGATAAA   420
          ***** ******************************************************

719       AAGTTAGTTAGTTTTATCAATAACTTTGATGATAGTGAAAAAGAACCAACGGTTTTACCA   480
20        AAGTTAGTTAGTTTTATCAATAACTTTGATGATAGTGAAAAAGAACCAACGGTTTTACCA   480
735       AAGTTAGTTAGTTTTATCAATAACTTTGATGATAGTGAAAAAGAACCAACGGTTTTACCA   480
649       AAGTTAGTTAGTTTTATCAATAACTTTGATGATAGTGAAAAAGAACCAACGGTTTTACCA   480
710       AAGTTAGTTAGTTTTATCAATAACTTTGATGATAGTGAAAAAGAACCAACGGTTTTACCA   480
729       AAGTTAGTTAGTTTTATCAATAACTTTGATGATAGTGAAAAAGAACCAACGGTTTTACCA   480
38        AAGTTAGTTAGTTTTATCAATAACTTTGATGATAGTGAAAAAGAACCAACGGTTTTACCA   480
600       AAGTTAGTTAGTTTTATCAATAACTTTGATGATAGTGAAAAAGAACCAACGGTTTTACCA   480
122       AAGTTAGTTAGTTTTATCAATAACTTTGATGATAGTGAAAAAGAACCAACGGTTTTACCA   480
683       AAGTTAGTTAGTTTTATCAATAACTTTGATGATAGTGAAAAAGAACCAACGGTTTTACCA   480
1026      AAGTTAGTTAGTTTTATCAATAACTTTGATGATAGTGAAAAAGAACCAACGGTTTTACCA   480
753       AAGTTAGTTAGTTTTATCAATAACTTTGATGATAGTGAAAAAGAACCAACGGTTTTACCA   480
720       AAGTTAGTTAGTTTTATCAATAACTTTGATGATAGTGAAAAAGAACCAACGGTTTTACCA   480
47        AAGTTAGTTAGTTTTATCAATAACTTTGATGATAGTGAAAAAGAACCAACGGTTTTACCA   480
663       AAGTTAGTTAGTTTTATCAATAACTTTGATGATAGTGAAAAAGAACCAACGGTTTTACCA   480
1014      AAGTTAGTTAGTTTTATCAATAACTTTGATGATAGTGAAAAAGAACCAACGGTTTTACCA   480
L43967    AAGTTAGTTAGTTTTATCAATAACTTTGATGATAGTGAAAAAGAACCAACGGTTTTACCA   480
656       AAGTTAGTTAGTTTTATCAATAACTTTGATGATAGTGAAAAAGAACCAACGGTTTTACCA   480
          ************************************************************

719       ACCTTACTGCCTAACCTCTTTATCAATGGTGCGAGTGGGATAGCTGCTGGATATGCAACT   540
20        ACCTTACTGCCTAACCTCTTTATCAATGGTGCGAGTGGGATAGCTGCTGGATATGCAACT   540
735       ACCTTACTGCCTAACCTCTTTATCAATGGTGCGAGTGGGATAGCTGCTGGATATGCAACT   540
649       ACCTTACTGCCTAACCTCTTTATCAATGGTGCGAGTGGGATAGCTGCTGGATATGCAACT   540
```

```
710        ACCTTACTGCCTAACCTCTTTATCAATGGTGCGAGTGGGATAGCTGCTGGATATGCAACT   540
729        ACCTTACTGCCTAACCTCTTTATCAATGGTGCGAGTGGGATAGCTGCTGGATATGCAACT   540
38         ACCTTACTGCCTAACCTCTTTATCAATGGTGCGAGTGGGATAGCTGCTGGATATGCAACT   540
600        ACCTTACTGCCTAACCTCTTTATCAATGGTGCGAGTGGGATAGCTGCTGGATATGCAACT   540
122        ACCTTACTGCCTAACCTCTTTATCAATGGTGCGAGTGGGATAGCTGCTGGATATGCAACT   540
683        ACCTTACTGCCTAACCTCTTTATCAATGGTGCGAGTGGGATAGCTGCTGGATATGCAACT   540
1026       ACCTTACTGCCTAACCTCTTTATCAATGGTGCGAGTGGGATAGCTGCTGGATATGCAACT   540
753        ACCTTACTGCCTAACCTCTTTATCAATGGTGCGAGTGGGATAGCTGCTGGATATGCAACT   540
720        ACCTTACTGCCTAACCTCTTTATCAATGGTGCGAGTGGGATAGCTGCTGGATATGCAACT   540
47         ACCTTACTGCCTAACCTCTTTATCAATGGTGCGAGTGGGATAGCTGCTGGATATGCAACT   540
663        ACCTTACTGCCTAACCTCTTTATCAATGGTGCGAGTGGGATAGCTGCTGGATATGCAACT   540
1014       ACCTTACTGCCTAACCTCTTTATCAATGGTGCGAGTGGGATAGCTGCTGGATATGCAACT   540
L43967     ACCTTACTGCCTAACCTCTTTATCAATGGTGCGAGTGGGATAGCTGCTGGATATGCAACT   540
656        ACCTTACTGCCTAACCTCTTTATCAATGGTGCGAGTGGGATAGCTGCTGGATATGCAACT   540
           ************************************************************

719        AATATTGCTCCCCATAACACTAATGAACTATTAGATAGTCTTTGCTTGCGAATAGACCAA   600
20         AATATTGCTCCCCATAACACTAATGAACTATTAGATAGTCTTTGCTTGCGAATAGACCAA   600
735        AATATTGCTCCCCATAACACTAATGAACTATTAGATAGTCTTTGCTTGCGAATAGACCAA   600
649        AATATTGCTCCCCATAACACTAATGAACTATTAGATAGTCTTTGCTTGCGAATAGACCAA   600
710        AATATTGCTCCCCATAACACTAATGAACTATTAGATAGTCTTTGCTTGCGAATAGACCAA   600
729        AATATTGCTCCCCATAACACTAATGAACTATTAGATAGTCTTTGCTTGCGAATAGACCAA   600
38         AATATTGCTCCCCATAACACTAATGAACTATTAGATAGTCTTTGCTTGCGAATAGACCAA   600
600        AATATTGCTCCCCATAACACTAATGAACTATTAGATAGTCTTTGCTTGCGAATAGACCAA   600
122        AATATTGCTCCCCATAACACTAATGAACTATTAGATAGTCTTTGCTTGCGAATAGACCAA   600
683        AATATTGCTCCCCATAACACTAATGAACTATTAGATAGTCTTTGCTTGCGAATAGACCAA   600
1026       AATATTGCTCCCCATAACACTAATGAACTATTAGATAGTCTTTGCTTGCGAATAGACCAA   600
753        AATATTGCTCCCCATAACACTAATGAACTATTAGATAGTCTTTGCTTGCGAATAGACCAA   600
720        AATATTGCTCCCCATAACACTAATGAACTATTAGATAGTCTTTGCTTGCGAATAGACCAA   600
47         AATATTGCTCCCCATAACACTAATGAACTATTAGATAGTCTTTGCTTGCGAATAGACCAA   600
663        AATATTGCTCCCCATAACACTAATGAACTATTAGATAGTCTTTGCTTGCGAATAGACCAA   600
1014       AATATTGCTCCCCATAACACTAATGAACTATTAGATAGTCTTTGCTTGCGAATAGACCAA   600
L43967     AATATTGCTCCCCATAACACTAATGAACTATTAGATAGTCTTTGCTTGCGAATAGACCAA   600
656        AATATTGCTCCCCATAACACTAATGAACTATTAGATAGTCTTTGCTTGCGAATAGACCAA   600
           ************************************************************

719        CCTGATTGTGAACTTAAACAAATTTTAAAAATTGTTAAAGGTCCTGATTTTCCAACAGGG   660
20         CCTGATTGTGAACTTAAACAAATTTTAAAAATTGTTAAAGGTCCTGATTTTCCAACAGGG   660
735        CCTGATTGTGAACTTAAACAAATTTTAAAAATTGTTAAAGGTCCTGATTTTCCAACAGGG   660
649        CCTGATTGTGAACTTAAACAAATTTTAAAAATTGTTAAAGGTCCTGATTTTCCAACAGGG   660
710        CCTGATTGTGAACTTAAACAAATTTTAAAAATTGTTAAAGGTCCTGATTTTCCAACAGGG   660
729        CCTGATTGTGAACTTAAACAAATTTTAAAAATTGTTAAAGGTCCTGATTTTCCAACAGGG   660
38         CCTGATTGTGAACTTAAACAAATTTTAAAAATTGTTAAAGGTCCTGATTTTCCAACAGGG   660
600        CCTGATTGTGAACTTAAACAAATTTTAAAAATTGTTAAAGGTCCTGATTTTCCAACAGGG   660
122        CCTGATTGTGAACTTAAACAAATTTTAAAAATTGTTAAAGGTCCTGATTTTCCAACAGGG   660
683        CCTGATTGTGAACTTAAACAAATTTTAAAAATTGTTAAAGGTCCTGATTTTCCAACAGGG   660
1026       CCTGATTGTGAACTTAAACAAATTTTAAAAATTGTTAAAGGTCCTGATTTTCCAACAGGG   660
753        CCTGATTGTGAACTTAAACAAATTTTAAAAATTGTTAAAGGTCCTGATTTTCCAACAGGG   660
720        CCTGATTGTGAACTTAAACAAATTTTAAAAATTGTTAAAGGTCCTGATTTTCCAACAGGG   660
47         CCTGATTGTGAACTTAAACAAATTTTAAAAATTGTTAAAGGTCCTGATTTTCCAACAGGG   660
663        CCTGATTGTGAACTTAAACAAATTTTAAAAATTGTTAAAGGTCCTGATTTTCCAACAGGG   660
1014       CCTGATTGTGAACTTAAACAAATTTTAAAAATTGTTAAAGGTCCTGATTTTCCAACAGGG   660
L43967     CCTAATTGTGAACTTAAACAAATTTTAAAAATTGTTAAAGGTCCTGATTTTCCAACAGGG   660
656        CCTGATTGTGAACTTAAACAAATTTTAAAAATTGTTAAAGGTCCTGATTTTCCAACAGGG   660
           *** ********************************************************

719        GGTAATGTTTATTCTGAAAAGAGTTTAAGTGATATTTATCAAGCAGGCAAAGGTAAATTT   720
20         GGTAATGTTTATTTTGAAAAGAGTTTAAGTGATATTTATCAAGCAGGCAAAGGTAAATTT   720
735        GGTAATGTTTATTTTGAAAAGAGTTTAAGTGATATTTATCAAGCAGGCAAAGGTAAATTT   720
649        GGTAATGTTTATTTTGAAAAGAGTTTAAGTGATATTTATCAAGCAGGCAAAGGTAAATTT   720
710        GGTAATGTTTATTTTGAAAAGAGTTTAAGTGATATTTATCAAGCAGGCAAAGGTAAATTT   720
729        GGTAATGTTTATTTTGAAAAGAGTTTAAGTGATATTTATCAAGCAGGCAAAGGTAAATTT   720
38         GGTAATGTTTATTTTGAAAAGAGTTTAAGTGATATTTATCAAGCAGGCAAAGGTAAATTT   720
600        GGTAATGTTTATTTTGAAAAGAGTTTAAGTGATATTTATCAAGCAGGCAAAGGTAAATTT   720
122        GGTAATGTTTATTTTGAAAAGAGTTTAAGTGATATTTATCAAGCAGGCAAAGGTAAATTT   720
683        GGTAATGTTTATTTTGAAAAGAGTTTAAGTGATATTTATCAAGCAGGCAAAGGTAAATTT   720
1026       GGTAATGTTTATTTTGAAAAGAGTTTAAGTGATATTTATCAAGCAGGCAAAGGTAAATTT   720
753        GGTAATGTTTATTTTGAAAAGAGTTTAAGTGATATTTATCAAGCAGGCAAAGGTAAATTT   720
720        GGTAATGTTTATTTTGAAAAGAGTTTAAGTGATATTTATCAAGCAGGCAAAGGTAAATTT   720
47         GGTAATGTTTATTTTGAAAAGAGTTTAAGTGATATTTATCAAGCAGGCAAAGGTAAATTT   720
663        GGTAATGTTTATTTTGAAAAGAGTTTAAGTGATATTTATCAAGCAGGCAAAGGTAAATTT   720
1014       GGTAATGTTTATTTTGAAAAGAGTTTAAGTGATATTTATCAAGCAGGCAAAGGTAAATTT   720
L43967     GGTAATGTTTATTTTGAAAAGAGTTTAAGTGATATTTATCAAGCAGGCAAAGGTAAATTT   720
656        GGTAATGTTTATTTTGAAAAGAGTTTAAGTGATATTTATCAAGCAGGCAAAGGTAAATTT   720
           ************* **********************************************

719        ATTATCCAAGCTAAGTATGAAGTTAACAAGAACTTAAACCAGATTGAAATTACCCAAATC   780
20         ATTATCCAAGCTAAGTATGAAGTTAACAAGAACTTAAACCAGATTGAAATTACCCAAATC   780
735        ATTATCCAAGCTAAGTATGAAGTTAACAAGAACTTAAACCAGATTGAAATTACCCAAATC   780
649        ATTATCCAAGCTAAGTATGAAGTTAACAAGAACTTAAACCAGATTGAAATTACCCAAATC   780
710        ATTATCCAAGCTAAGTATGAAGTTAACAAGAACTTAAACCAGATTGAAATTACCCAAATC   780
729        ATTATCCAAGCTAAGTATGAAGTTAACAAGAACTTAAACCAGATTGAAATTACCCAAATC   780
38         ATTATCCAAGCTAAGTATGAAGTTAACAAGAACTTAAACCAGATTGAAATTACCCAAATC   780
```

```
600         ATTATCCAAGCTAAGTATGAAGTTAACAAGAACTTAAACCAGATTGAAATTACCCAAATC   780
122         ATTATCCAAGCTAAGTATGAAGTTAACAAGAACTTAAACCAGATTGAAATTACCCAAATC   780
683         ATTATCCAAGCTAAGTATGAAGTTAACAAGAACTTAAACCAGATTGAAATTACCCAAATC   780
1026        ATTATCCAAGCTAAGTATGAAGTTAACAAGAACTTAAACCAGATTGAAATTACCCAAATC   780
753         ATTATCCAAGCTAAGTATGAAGTTAACAAGAATTTAAACCAGATTGAAATTACCCAAATC   780
720         ATTATCCAAGCTAAGTATGAAGTTAACAAGAACTTAAACCAGATTGAAATTACCCAAATC   780
47          ATTATCCAAGCTAAGTATGAAGTTAACAAGAACTTAAACCAGATTGAAATTACCCAAATC   780
663         ATTATCCAAGCTAAGTATGAAGTTAACAAGAACTTAAACCAGATTGAAATTACCCAAATC   780
1014        ATTATCCAAGCTAAGTATGAAGTTAACAAGAACTTAAACCAGATTGAAATTACCCAAATC   780
L43967      ATTATCCAAGCTAAGTATGAAGTTAACAAGAACTTAAACCAGATTGAAATTACCCAAATC   780
656         ATTATACAAGCTAAGTATGAAGTTAACAAGAACTTAAACCAGATTGAAATTACCCAAATC   780
            ***** ************************** ***************************

719         CCTTATGAAACACTGAAAGCTAACATTATCAAACAAATTGAAGAGATTATCTTTGACAAT   840
20          CCTTATGAAACACTGAAAGCTAACATTATCAAACAAATTGAAGAGATTATCTTTGACAAT   840
735         CCTTATGAAACACTGAAAGCTAACATTATCAAACAAATTGAAGAGATTATCTTTGACAAT   840
649         CCTTATGAAACACTGAAAGCTAACATTATCAAACAAATTGAAGAGATTATCTTTGACAAT   840
710         CCTTATGAAACACTGAAAGCTAACATTATCAAACAAATTGAAGAGATTATCTTTGACAAT   840
729         CCTTATGAAACACTGAAAGCTAACATTATCAAACAAATTGAAGAGATTATCTTTGACAAT   840
38          CCTTATGAAACACTGAAAGCTAACATTATCAAACAAATTGAAGAGATTATCTTTGACAAT   840
600         CCTTATGAAACACTGAAAGCTAACATTATCAAACAAATTGAAGAGATTATCTTTGACAAT   840
122         CCTTATGAAACACTGAAAGCTAACATTATCAAACAAATTGAAGAGATTATCTTTGACAAT   840
683         CCTTATGAAACACTGAAAGCTAACATTGTCAAACAAATTGAAGAGATTATCTTTGACAAT   840
1026        CCTTATGAAACACTGAAAGCTAACATTGTCAAACAAATTGAAGAGATTATCTTTGACAAT   840
753         CCTTATGAAACACTGAAAGCTAACATTGTCAAACAAATTGAAGAGATTATCTTTGACAAT   840
720         CCTTATGAAACACTGAAAGCTAACATTGTCAAACAAATTGAAGAGATTATCTTTGACAAT   840
47          CCTTATGAAACACTGAAAGCTAACATTGTCAAACAAATTGAAGAGATTATCTTTGACAAT   840
663         CCTTATGAAACACTGAAAGCTAACATTGTCAAACAAATTGAAGAGATTATCTTTGACAAT   840
1014        CCTTATGAAACACTGAAAGCTAACATTGTCAAACAAATTGAAGAGATTATCTTTGACAAT   840
L43967      CCTTATGAAACACTGAAAGCTAACATTGTCAAACAAATTGAAGAGATTATCTTTGACAAT   840
656         CCTTATGAAACACTGAAAGCTAACATTGTCAAACAAATTGAAGAGATTATCTTTGACAAT   840
            *************************** ********************************

719         AAACTATCTGCTATTGAAAGTGTCATTGATAGTTCAGATCGCAACGGCATTAGGATCATT   900
20          AAACTATCTGCTATTGAAAGTGTCATTGATAGTTCAGATCGCAACGGCATTAGGATCATT   900
735         AAACTATCTGCTATTGAAAGTGTCATTGATAGTTCAGATCGCAACGGCATTAGGATCATT   900
649         AAACTATCTGCTATTGAAAGTGTCATTGATAGTTCAGATCGCAACGGCATTAGGATCATT   900
710         AAACTATCTGCTATTGAAAGTGTCATTGATAGTTCAGATCGCAACGGCATTAGGATCATT   900
729         AAACTATCTGCTATTGAAAGTGTCATTGATAGTTCAGATCGCAACGGCATTAGGATCATT   900
38          AAACTATCTGCTATTGAAAGTGTCATTGATAGTTCAGATCGCAACGGCATTAGGATCATT   900
600         AAACTATCTGCTATTGAAAGTGTCATTGATAGTTCAGATCGCAACGGCATTAGGATCATT   900
122         AAACTATCTGCTATTGAAAGTGTCATTGATAGTTCAGATCGCAACGGCATTAGGATCATT   900
683         AAACTATCTGCTATTGAAAGTGTCATTGATAGTTCAGATCGCAACGGCATTAGGATCATT   900
1026        AAACTATCTGCTATTGAAAGTGTCATTGATAGTTCAGATCGCAACGGCATTAGGATCATT   900
753         AAACTATCTGCTATTGAAAGTGTCATTGATAGTTCAGATCGCAACGGCATTAGGATCATT   900
720         AAACTATCTGCTATTGAAAGTGTCATTGATAGTTCAGATCGCAACGGCATTAGGATCATT   900
47          AAACTATCTGCTATTGAAAGTGTCATTGATAGTTCAGATCGCAACGGCATTAGGATCATT   900
663         AAACTATCTGCTATTGAAAGTGTCATTGATAGTTCAGATCGCAACGGCATTAGGATCATT   900
1014        AAACTATCTGCTATTGAAAGTGTCATTGATAGTTCAGATCGCAACGGCATTAGGATCATT   900
L43967      AAACTATCTGCTATTGAAAGTGTCATTGATAGTTCAGATCGCAACGGCATTAGGATCATT   900
656         AAACTATCTGCTATTGAAAGTGTCATTGATAGTTCAGATCGCAACGGCATTAGGATCATT   900
            ************************************************************

719         ATTAAACACAAGGACTTTTTGCCTGCTGAGAAGATCATGGCCTTTTTGTTTAAACACACC   960
20          ATTAAACACAAGGACTTTTTGCCTGCTGAGAAGATCATGGCCTTTTTGTTTAAACACACC   960
735         ATTAAACACAAGGACTTTTTGCCTGCTGAGAAGATCATGGCCTTTTTGTTTAAACACACC   960
649         ATTAAACACAAGGACTTTTTGCCTGCTGAGAAGATCATGGCCTTTTTGTTTAAACACACC   960
710         ATTAAACACAAGGACTTTTTGCCTGCTGAGAAGATCATGGCCTTTTTGTTTAAACACACC   960
729         ATTAAACACAAGGACTTTTTGCCTGCTGAGAAGATCATGGCCTTTTTGTTTAAACACACC   960
38          ATTAAACACAAGGACTTTTTGCCTGCTGAGAAGATCATGGCCTTTTTGTTTAAACACACC   960
600         ATTAAACACAAGGACTTTTTGCCTGCTGAGAAGATCATGGCCTTTTTGTTTAAACACACC   960
122         ATTAAACACAAGGACTTTTTGCCTGCTGAGAAGATCATGGCCTTTTTGTTTAAACACACC   960
683         ATTAAACACAAGGACTTTTTGCCTGCTGAGAAGATCATGGCCTTTTTGTTTAAACACACC   960
1026        ATTAAACACAAGGACTTTTTGCCTGCTGAGAAGATCATGGCCTTTTTGTTTAAACACACC   960
753         ATTAAACACAAGGACTTTTTGCCTGCTGAGAAGATCATGGCCTTTTTGTTTAAACACACC   960
720         ATTAAACACAAGGACTTTTTGCCTGCTGAGAAGATCATGGCCTTTTTGTTTAAACACACC   960
47          ATTAAACACAAGGACTTTTTGCCTGCTGAGAAGATCATGGCCTTTTTGTTTAAACACACC   960
663         ATTAAACACAAGGACTTTTTGCCTGCTGAGAAGATCATGGCCTTTTTGTTTAAACACACC   960
1014        ATTAAACACAAGGACTTTTTGCCTGCTGAGAAGATCATGGCCTTTTTGTTTAAACACACC   960
L43967      ATTAAACACAAGGACTTTTTGCCTGCTGAGAAGATCATGGCCTTTTTGTTTAAACACACC   960
656         ATTAAACACAAGGACTTTTTGCCTGCTGAGAAGATCATGGCCTTTTTGTTTAAACACACC   960
            ************************************************************

719         CAACTCCAAGTGAACTTTAACCTTAATAACACCGTGATTGCTAACCGCTTTCCCATCCAA   1020
20          CAACTCCAAGTGAACTTTAACCTTAATAACACCGTGATTGCTAACCGCTTTCCCATCCAA   1020
735         CAACTCCAAGTGAACTTTAACCTTAATAACACCGTGATTGCTAACCGCTTTCCCATCCAA   1020
649         CAACTCCAAGTGAACTTTAACCTTAATAACACCGTGATTGCTAACCGCTTTCCCATCCAA   1020
710         CAACTCCAAGTGAACTTTAACCTTAATAACACCGTGATTGCTAACCGCTTTCCCATCCAA   1020
729         CAACTCCAAGTGAACTTTAACCTTAATAACACCGTGATTGCTAACCGCTTTCCCATCCAA   1020
38          CAACTCCAAGTGAACTTTAACCTTAATAACACCGTGATTGCTAACCGCTTTCCCATCCAA   1020
600         CAACTCCAAGTGAACTTTAACCTTAATAACACCGTGATTGCTAACCGCTTTCCCATCCAA   1020
122         CAACTCCAAGTGAACTTTAACCTTAATAACACCGTGATTGCTAACCGCTTTCCCATCCAA   1020
683         CAACTCCAAGTGAACTTTAACCTTAATAACACCGTGATTGCTAACCGCTTTCCCATCCAA   1020
```

```
1026      CAACTCCAAGTGAACTTTAACCTTAATAACACCGTGATTGCTAACCGCTTTCCCATCCAA   1020
753       CAACTCCAAGTGAACTTTAACCTTAATAACACCGTGATTGCTAACCGCTTTCCCATCCAA   1020
720       CAACTCCAAGTGAACTTTAACCTTAATAACACCGTGATTGCTAACCGCTTTCCCATCCAA   1020
47        CAACTCCAAGTGAACTTTAACCTTAATAACACCGTGATTGCTAACCGCTTTCCCATCCAA   1020
663       CAACTCCAAGTGAACTTTAACCTTAATAACACCGTGATTGCTAACCGCTTTCCCATCCAA   1020
1014      CAACTCCAAGTGAACTTTAACCTTAATAACACCGTGATTGCTAACCGCTTTCCCATCCAA   1020
L43967    CAACTCCAAGTGAACTTTAACCTTAATAACACCGTGATTGCTAACCGCTTTCCCATCCAA   1020
656       CAACTCCAAGTGAACTTTAACCTTAATAACACCGTGATTGCTAACCGCTTTCCCATCCAA   1020
          ************************************************************

719       ATTGGTTTACTAAGTTACCTCGATTATTTTTTAAAGTTTTGTCATGAACTAATTATTAAT   1080
20        ATTGGTTTACTAAGTTACCTCGATTATTTTTTAAAGTTTTGTCATGAACTAATTATTAAT   1080
735       ATTGGTTTACTAAGTTACCTCGATTATTTTTTAAAGTTTTGTCATGAACTAATTATTAAT   1080
649       ATTGGTTTACTAAGTTACCTCGATTATTTTTTAAAGTTTTGTCATGAACTAATTATTAAT   1080
710       ATTGGTTTACTAAGTTACCTCGATTATTTTTTAAAGTTTTGTCATGAACTAATTATTAAT   1080
729       ATTGGTTTACTAAGTTACCTCGATTATTTTTTAAAGTTTTGTCATGAACTAATTATTAAT   1080
38        ATTGGTTTACTAAGTTACCTCGATTATTTTTTAAAGTTTTGTCATGAACTAATTATTAAT   1080
600       ATTGGTTTACTAAGTTACCTCGATTATTTTTTAAAGTTTTGTCATGAACTAATTATTAAT   1080
122       ATTGGTTTACTAAGTTACCTCGATTATTTTTTAAAGTTTTGTCATGAACTAATTATTAAT   1080
683       ATTGGTTTACTAAGTTACCTCGATCATTTTTTAAAGTTTTGTCATGAACTAATTATTAAT   1080
1026      ATTGGTTTACTAAGTTACCTCGATCATTTTTTAAAGTTTTGTCATGAACTAATTATTAAT   1080
753       ATTGGTTTACTAAGTTACCTCGATCATTTTTTAAAGTTTTGTCATGAACTAATTATTAAT   1080
720       ATTGGTTTACTAAGTTACCTCGATCATTTTTTAAAGTTTTGTCATGAACTAATTATTAAT   1080
47        ATTGGTTTACTAAGTTACCTCGATCATTTTTTAAAGTTTTGTCATGAACTAATTATTAAT   1080
663       ATTGGTTTACTAAGTTACCTCGATCATTTTTTAAAGTTTTGTCATGAACTAATTATTAAT   1080
1014      ATTGGTTTACTAAGTTACCTCGATCATTTTTTAAAGTTTTGTCATGAACTAATTATTAAT   1080
L43967    ATTGGTTTACTAAGTTACCTCGATCATTTTTTAAAGTTTTGTCATGAACTAATTATTAAT   1080
656       ATTGGTTTACTAAGTTACCTCGATCATTTTTTAAAGTTTTGTCATGAACTAATTATTAAT   1080
          ************************ ***********************************

719       AAAGCTAAGTATGAACTTGAGCTTGCAAGCAAGCGCTTGGAAATTATTTTAGGACTAATT   1140
20        AAAGCTAAGTATGAACTTGAGCTTGCAAGCAAGCGCTTGGAAATTATTTTAGGACTAATT   1140
735       AAAGCTAAGTATGAACTTGAGCTTGCAAGCAAGCGCTTGGAAATTATTTTAGGACTAATT   1140
649       AAAGCTAAGTATGAACTTGAGCTTGCAAGCAAGCGCTTGGAAATTATTTTAGGACTAATT   1140
710       AAAGCTAAGTATGAACTTGAGCTTGCAAGCAAGCGCTTGGAAATTATTTTAGGACTAATT   1140
729       AAAGCTAAGTATGAACTTGAGCTTGCAAGCAAGCGCTTGGAAATTATTTTAGGACTAATT   1140
38        AAAGCTAAGTATGAACTTGAGCTTGCAAGCAAGCGCTTGGAAATTATTTTAGGACTAATT   1140
600       AAAGCTAAGTATGAACTTGAGCTTGCAAGCAAGCGCTTGGAAATTATTTTAGGACTAATT   1140
122       AAAGCTAAGTATGAACTTGAGCTTGCAAGCAAGCGCTTGGAAATTATTTTAGGACTAATT   1140
683       AAAGCTAAGTATGAACTTGAGCTTGCAAGCAAGCGCTTGGAAATTATTTTAGGACTAATT   1140
1026      AAAGCTAAGTATGAACTTGAGCTTGCAAGCAAGCGCTTGGAAATTATTTTAGGACTAATT   1140
753       AAAGCTAAGTATGAACTTGAGCTTGCAAGCAAGCGCTTGGAAATTATTTTAGGACTAATT   1140
720       AAAGCTAAGTATGAACTTGAGCTTGCAAGCAAGCGCTTGGAAATTATTTTAGGACTAATT   1140
47        AAAGCTAAGTATGAACTTGAGCTTGCAAGCAAGCGCTTGGAAATTATTTTAGGACTAATT   1140
663       AAAGCTAAGTATGAACTTGAGCTTGCAAGCAAGCGCTTGGAAATTATTTTAGGACTAATT   1140
1014      AAAGCTAAGTATGAACTTGAGCTTGCAAGCAAGCGCTTGGAAATTATTTTAGGACTAATT   1140
L43967    AAAGCTAAGTATGAACTTGAGCTTGCAAGCAAGCGCTTGGAAATTATTTTAGGACTAATT   1140
656       AAAGCTAAGTATGAACTTGAGCTTGCAAGCAAGCGCTTGGAAATTATTTTAGGACTAATT   1140
          ************************************************************

719       AAAGCGATTAGTATCATTGATAAAATCATCAAATTAATTAGATCAGCAGTTGACAAAAGT   1200
20        AAAGCGATTAGTATCATTGATAAAATCATCAAATTAATTAGATCAGCAGTTGACAAAAGT   1200
735       AAAGCGATTAGTATCATTGATAAAATCATCAAATTAATTAGATCAGCAGTTGACAAAAGT   1200
649       AAAGCGATTAGTATCATTGATAAAATCATCAAATTAATTAGATCAGCAGTTGACAAAAGT   1200
710       AAAGCGATTAGTATCATTGATAAAATCATCAAATTAATTAGATCAGCAGTTGACAAAAGT   1200
729       AAAGCGATTAGTATCATTGATAAAATCATCAAATTAATTAGATCAGCAGTTGACAAAAGT   1200
38        AAAGCGATTAGTATCATTGATAAAATCATCAAATTAATTAGATCAGCAGTTGACAAAAGT   1200
600       AAAGCGATTAGTATCATTGATAAAATCATCAAATTAATTAGATCAGCAGTTGACAAAAGT   1200
122       AAAGCGATTAGTATCATTGATAAAATCATCAAATTAATTAGATCAGCAGTTGACAAAAGT   1200
683       AAAGCGATTAGTATCATTGATAAAATCATCAAATTAATTAGATCAGCAGTTGACAAAAGT   1200
1026      AAAGCGATTAGTATCATTGATAAAATCATCAAATTAATTAGATCAGCAGTTGACAAAAGT   1200
753       AAAGCGATTAGTATCATTGATAAAATCATCAAATTAATTAGATCAGCAGTTGACAAAAGT   1200
720       AAAGCGATTAGTATCATTGATAAAATCATCAAATTAATTAGATCAGCAGTTGACAAAAGT   1200
47        AAAGCGATTAGTATCATTGATAAAATCATCAAATTAATTAGATCAGCAGTTGACAAAAGT   1200
663       AAAGCGATTAGTATCATTGATAAAATCATCAAATTAATTAGATCAGCAGTTGACAAAAGT   1200
1014      AAAGCGATTAGTATCATTGATAAAATCATCAAATTAATTAGATCAGCAGTTGACAAAAGT   1200
L43967    AAAGCGATTAGTATCATTGATAAAATCATCAAATTAATTAGATCAGCAGTTGACAAAAGT   1200
656       AAAGCGATTAGTATCATTGATAAAATCATCAAATTAATTAGATCAGCAGTTGACAAAAGT   1200
          ************************************************************

719       GATGCAAGAGAAAAGTTAATTGATAACTTTAAATTTACTTTTAACCAAGCAGAGGCAATT   1260
20        GATGCAAGAGAAAAGTTAATTGATAACTTTAAATTTACTTTTAACCAAGCAGAGGCAATT   1260
735       GATGCAAGAGAAAAGTTAATTGATAACTTTAAATTTACTTTTAACCAAGCAGAGGCAATT   1260
649       GATGCAAGAGAAAAGTTAATTGATAACTTTAAATTTACTTTTAACCAAGCAGAGGCAATT   1260
710       GATGCAAGAGAAAAGTTAATTGATAACTTTAAATTTACTTTTAACCAAGCAGAGGCAATT   1260
729       GATGCAAGAGAAAAGTTAATTGATAACTTTAAATTTACTTTTAACCAAGCAGAGGCAATT   1260
38        GATGCAAGAGAAAAGTTAATTGATAACTTTAAATTTACTTTTAACCAAGCAGAGGCAATT   1260
600       GATGCAAGAGAAAAGTTAATTGATAACTTTAAATTTACTTTTAACCAAGCAGAGGCAATT   1260
122       GATGCAAGAGAAAAGTTAATTGATAACTTTAAATTTACTTTTAACCAAGCAGAGGCAATT   1260
683       GATGCAAGAGAAAAGTTAATTGATAACTTTAAATTTACTTTTAACCAAGCAGAGGCAATT   1260
1026      GATGCAAGAGAAAAGTTAATTGATAACTTTAAATTTACTTTTAACCAAGCAGAGGCAATT   1260
753       GATGCAAGAGAAAAGTTAATTGATAACTTTAAATTTACTTTTAACCAAGCAGAGGCAATT   1260
720       GATGCAAGAGAAAAGTTAATTGATAACTTTAAATTTACTTTTAACCAAGCAGAGGCAATT   1260
```

```
47          GATGCAAGAGAAAAGTTAATTGATAACTTTAAATTTACTTTTAACCAAGCAGAGGCAATT   1260
663         GATGCAAGAGAAAAGTTAATTGATAACTTTAAATTTACTTTTAACCAAGCAGAGGCAATT   1260
1014        GATGCAAGAGAAAAGTTAATTGATAACTTTAAATTTACTTTTAACCAAGCAGAGGCAATT   1260
L43967      GATGCAAGAGAAAAGTTAATTGATAACTTTAAATTTACTTTTAACCAAGCAGAGGCAATT   1260
656         GATGCAAGAGAAAAGTTAATTGATAACTTTAAATTTACTTTTAACCAAGCAGAGGCAATT   1260
            ************************************************************

719         GTTAGTTTGCGACTTTACCAACTAACTAACACTGATATTTTTGAACTTAACCAAGAACAA   1320
20          GTTAGTTTGCGACTTTACCAACTAACTAACACTGATATTTTTGAACTTAACCAAGAACAA   1320
735         GTTAGTTTGCGACTTTACCAACTAACTAACACTGATATTTTTGAACTTAACCAAGAACAA   1320
649         GTTAGTTTGCGACTTTACCAACTAACTAACACTGATATTTTTGAACTTAACCAAGAACAA   1320
710         GTTAGTTTGCGACTTTACCAACTAACTAACACTGATATTTTTGAACTTAACCAAGAACAA   1320
729         GTTAGTTTGCGACTTTACCAACTAACTAACACTGATATTTTTGAACTTAACCAAGAACAA   1320
38          GTTAGTTTGCGACTTTACCAACTAACTAACACTGATATTTTTGAACTTAACCAAGAACAA   1320
600         GTTAGTTTGCGACTTTACCAACTAACTAACACTGATATTTTTGAACTTAACCAAGAACAA   1320
122         GTTAGTTTGCGACTTTACCAACTAACTAACACTGATATTTTTGAACTTAACCAAGAACAA   1320
683         GTTAGTTTGCGACTTTACCAACTAACTAACACTGATATTTTTGAACTTAACCAAGAACAA   1320
1026        GTTAGTTTGCGACTTTACCAACTAACTAACACTGATATTTTTGAACTTAACCAAGAACAA   1320
753         GTTAGTTTGCGACTTTACCAACTAACTAACACTGATATTTTTGAACTTAACCAAGAACAA   1320
720         GTTAGTTTGCGACTTTACCAACTAACTAACACTGATATTTTTGAACTTAACCAAGAACAA   1320
47          GTTAGTTTGCGACTTTACCAACTAACTAACACTGATATTTTTGAACTTAACCAAGAACAA   1320
663         GTTAGTTTGCGACTTTACCAACTAACTAACACTGATATTTTTGAACTTAACCAAGAACAA   1320
1014        GTTAGTTTGCGACTTTACCAACTAACTAACACTGATATTTTTGAACTTAACCAAGAACAA   1320
L43967      GTTAGTTTGCGACTTTACCAACTAACTAACACTGATATTTTTGAACTTAACCAAGAACAA   1320
656         GTTAGTTTGCGACTTTACCAACTAACTAACACTGATATTTTTGAACTTAACCAAGAACAA   1320
            ************************************************************

719         AATGAACTTGAAAAAACTGTGATTAGTTCAGAGCAACTAATTGCTAGTGAAAAAGCAAGA   1380
20          AATGAACTTGAAAAAACTGTGATTAGTTCAGAGCAACTAATTGCTAGTGAAAAAGCAAGA   1380
735         AATGAACTTGAAAAAACTGTGATTAGTTCAGAGCAACTAATTGCTAGTGAAAAAGCAAGA   1380
649         AATGAACTTGAAAAAACTGTGATTAGTTCAGAGCAACTAATTGCTAGTGAAAAAGCAAGA   1380
710         AATGAACTTGAAAAAACTGTGATTAGTTCAGAGCAACTAATTGCTAGTGAAAAAGCAAGA   1380
729         AATGAACTTGAAAAAACTGTGATTAGTTCAGAGCAACTAATTGCTAGTGAAAAAGCAAGA   1380
38          AATGAACTTGAAAAAACTGTGATTAGTTCAGAGCAACTAATTGCTAGTGAAAAAGCAAGA   1380
600         AATGAACTTGAAAAAACTGTGATTAGTTCAGAGCAACTAATTGCTAGTGAAAAAGCAAGA   1380
122         AATGAACTTGAAAAAACTGTGATTAGTTCAGAGCAACTAATTGCTAGTGAAAAAGCAAGA   1380
683         AATGAACTTGAAAAAACTGTGATTAGTTCAGAGCAACTAATTGCTAGTGAAAAAGCAAGA   1380
1026        AATGAACTTGAAAAAACTGTGATTAGTTCAGAGCAACTAATTGCTAGTGAAAAAGCAAGA   1380
753         AATGAACTTGAAAAAACTGTGATTAGTTCAGAGCAACTAATTGCTAGTGAAAAAGCAAGA   1380
720         AATGAACTTGAAAAAACTGTGATTAGTTCAGAGCAACTAATTGCTAGTGAAAAAGCAAGA   1380
47          AATGAACTTGAAAAAACTGTGATTAGTTCAGAGCAACTAATTGCTAGTGAAAAAGCAAGA   1380
663         AATGAACTTGAAAAAACTGTGATTAGTTCAGAGCAACTAATTGCTAGTGAAAAAGCAAGA   1380
1014        AATGAACTTGAAAAAACTGTGATTAGTTCAGAGCAACTAATTGCTAGTGAAAAAGCAAGA   1380
L43967      AATGAACTTGAAAAAACTGTGATTAGTTCAGAGCAACTAATTGCTAGTGAAAAAGCAAGA   1380
656         AATGAACTTGAAAAAACTGTGATTAGTTCAGAGCAACTAATTGCTAGTGAAAAAGCAAGA   1380
            ************************************************************

719         AACAAACTCCTAAAAAAACAGTTTGAAGGTTATAAAAAGCAGTTTCACCAGCAACGAAGG   1440
20          AACAAACTCCTAAAAAAACAGTTTGAAGGTTATAAAAAGCAGTTTCACCAGCAACGAAGG   1440
735         AACAAACTCCTAAAAAAACAGTTTGAAGGTTATAAAAAGCAGTTTCACCAGCAACGAAGG   1440
649         AACAAACTCCTAAAAAAACAGTTTGAAGGTTATAAAAAGCAGTTTCACCAGCAACGAAGG   1440
710         AACAAACTCCTAAAAAAACAGTTTGAAGGTTATAAAAAGCAGTTTCACCAGCAACGAAGG   1440
729         AACAAACTCCTAAAAAAACAGTTTGAAGGTTATAAAAAGCAGTTTCACCAGCAACGAAGG   1440
38          AACAAACTCCTAAAAAAACAGTTTGAAGGTTATAAAAAGCAGTTTCACCAGCAACGAAGG   1440
600         AACAAACTCCTAAAAAAACAGTTTGAAGGTTATAAAAAGCAGTTTCACCAGCAACGAAGG   1440
122         AACAAACTCCTAAAAAAACAGTTTGAAGGTTATAAAAAGCAGTTTCACCAGCAACGAAGG   1440
683         AACAAACTCCTAAAAAAACAGTTTGAAGGTTATAAAAAGCAGTTTCACCAGCAACGAAGG   1440
1026        AACAAACTCCTAAAAAAACAGTTTGAAGGTTATAAAAAGCAGTTTCACCAGCAACGAAGG   1440
753         AACAAACTCCTAAAAAAACAGTTTGAAGGTTATAAAAAGCAGTTTCACCAGCAACGAAGG   1440
720         AACAAACTCCTAAAAAAACAGTTTGAAGGTTATAAAAAGCAGTTTCACCAGCAACGAAGG   1440
47          AACAAACTCCTAAAAAAACAGTTTGAAGGTTATAAAAAGCAGTTTCACCAGCAACGAAGG   1440
663         AACAAACTCCTAAAAAAACAGTTTGAAGGTTATAAAAAGCAGTTTCACCAGCAACGAAGG   1440
1014        AACAAACTCCTAAAAAAACAGTTTGAAGGTTATAAAAAGCAGTTTCACCAGCAACGAAGG   1440
L43967      AACAAACTCCTAAAAAAACAGTTTGAAGGTTATAAAAAGCAGTTTCACCAGCAACGAAGG   1440
656         AACAAACTCCTAAAAAAACAGTTTGAAGGTTATAAAAAGCAGTTTCACCAGCAACGAAGG   1440
            ************************************************************

719         TCACAAATATGTGGCTTTATTAACCAAAAAAAGGTGGAGGAAAGTGAGCTAATTGAAAAC   1500
20          TCACAAATATGTGGCTTTATTAACCAAAAAAAGGTGGAGGAAAGTGAGCTAATTGAAAAC   1500
735         TCACAAATATGTGGCTTTATTAACCAAAAAAAGGTGGAGGAAAGTGAGCTAATTGAAAAC   1500
649         TCACAAATATGTGGCTTTATTAACCAAAAAAAGGTGGAGGAAAGTGAGCTAATTGAAAAC   1500
710         TCACAAATATGTGGCTTTATTAACCAAAAAAAGGTGGAGGAAAGTGAGCTAATTGAAAAC   1500
729         TCACAAATATGTGGCTTTATTAACCAAAAAAAGGTGGAGGAAAGTGAGCTAATTGAAAAC   1500
38          TCACAAATATGTGGCTTTATTAACCAAAAAAAGGTGGAGGAAAGTGAGCTAATTGAAAAC   1500
600         TCACAAATATGTGGCTTTATTAACCAAAAAAAGGTGGAGGAAAGTGAGCTAATTGAAAAC   1500
122         TCACAAATATGTGGCTTTATTAACCAAAAAAAGGTGGAGGAAAGTGAGCTAATTGAAAAC   1500
683         TCACAAATATGTGGCTTTATTAACCAAAAAAAGGTGGAGGAAAGTGAGCTAATTGAAAAC   1500
1026        TCACAAATATGTGGCTTTATTAACCAAAAAAAGGTGGAGGAAAGTGAGCTAATTGAAAAC   1500
753         TCACAAATATGTGGCTTTATTAACCAAAAAAAGGTGGAGGAAAGTGAGCTAATTGAAAAC   1500
720         TCACAAATATGTGGCTTTATTAACCAAAAAAAGGTGGAGGAAAGTGAGCTAATTGAAAAC   1500
47          TCACAAATATGTGGCTTTATTAACCAAAAAAAGGTGGAGGAAAGTGAGCTAATTGAAAAC   1500
663         TCACAAATATGTGGCTTTATTAACCAAAAAAAGGTGGAGGAAAGTGAGCTAATTGAAAAC   1500
1014        TCACAAATATGTGGCTTTATTAACCAAAAAAAGGTGGAGGAAAGTGAGCTAATTGAAAAC   1500
```

```
L43967     TCACAAATATGTGGCTTTATTAACCAAAAAAAGGTGGAGGAAAGTGAGCTAATTGAAAAC   1500
656        TCACAAATATGTGGCTTTATTAACCAAAAAAAGGTGGAGGAAAGTGAGCTAATTGAAAAC   1500
           ************************************************************

719        AAAACTTATGGGGTTTTAATCACTAAAGCTGGTAACTACCATAAGTTTGAATCTAACCAA   1560
20         AAAACTTATGGGGTTTTAATCACTAAAGCTGGTAACTACCATAAGTTTGAATCTAACCAA   1560
735        AAAACTTATGGGGTTTTAATCACTAAAGCTGGTAACTACCATAAGTTTGAATCTAACCAA   1560
649        AAAACTTATGGGGTTTTAATCACTAAAGCTGGTAACTACCATAAGTTTGAATCTAACCAA   1560
710        AAAACTTATGGGGTTTTAATCACTAAAGCTGGTAACTACCATAAGTTTGAATCTAACCAA   1560
729        AAAACTTATGGGGTTTTAATCACTAAAGCTGGTAACTACCATAAGTTTGAATCTAACCAA   1560
38         AAAACTTATGGGGTTTTAATCACTAAAGCTGGTAACTACCATAAGTTTGAATCTAACCAA   1560
600        AAAACTTATGGGGTTTTAATCACTAAAGCTGGTAACTACCATAAGTTTGAATCTAACCAA   1560
122        AAAACTTATGGGGTTTTAATCACTAAAGCTGGTAACTACCATAAGTTTGAATCTAACCAA   1560
683        AAAACTTATGGGGTTTTAATCACTAAAGCTGGTAACTACCATAAGTTTGAATCTAACCAA   1560
1026       AAAACTTATGGGGTTTTAATCACTAAAGCTGGTAACTACCATAAGTTTGAATCTAACCAA   1560
753        AAAACTTATGGGGTTTTAATCACTAAAGCTGGTAACTACCATAAGTTTGAATCTAACCAA   1560
720        AAAACTTATGGGGTTTTAATCACTAAAGCTGGTAACTACCATAAGTTTGAATCTAACCAA   1560
47         AAAACTTATGGGGTTTTAATCACTAAAGCTGGTAACTACCATAAGTTTGAATCTAACCAA   1560
663        AAAACTTATGGGGTTTTAATCACTAAAGCTGGTAACTACCATAAGTTTGAATCTAACCAA   1560
1014       AAAACTTATGGGGTTTTAATCACTAAAGCTGGTAACTACCATAAGTTTGAATCTAACCAA   1560
L43967     AAAACTTATGGGGTTTTAATCACTAAAGCTGGTAACTACCATAAGTTTGAATCTAACCAA   1560
656        AAAACTTATGGGGTTTTAATCACTAAAGCTGGTAACTACCATAAGTTTGAATCTAACCAA   1560
           ************************************************************

719        CTATTAAAAAGCACCACTGATTTTAAAAGTGAGAGTGACACAATTATCTTTGCACAAACT   1620
20         CTATTAAAAAGCACCACTGATTTTAAAAGTGAGAGTGACACAATTATCTTTGCACAAACT   1620
735        CTATTAAAAAGCACCACTGATTTTAAAAGTGAGAGTGACACAATTATCTTTGCACAAACT   1620
649        CTATTAAAAAGCACCACTGATTTTAAAAGTGAGAGTGACACAATTATCTTTGCACAAACT   1620
710        CTATTAAAAAGCACCACTGATTTTAAAAGTGAGAGTGACACAATTATCTTTGCACAAACT   1620
729        CTATTAAAAAGCACCACTGATTTTAAAAGTGAGAGTGACACAATTATCTTTGCACAAACT   1620
38         CTATTAAAAAGCACCACTGATTTTAAAAGTGAGAGTGACACAATTATCTTTGCACAAACT   1620
600        CTATTAAAAAGCACCACTGATTTTAAAAGTGAGAGTGACACAATTATCTTTGCACAAACT   1620
122        CTATTAAAAAGCACCACTGATTTTAAAAGTGAGAGTGACACAATTATCTTTGCACAAACT   1620
683        CTATTAAAAAGCACCACTGATTTTAAAAGTGAGAGTGACACAATTATCTTTGCACAAACT   1620
1026       CTATTAAAAAGCACCACTGATTTTAAAAGTGAGAGTGACACAATTATCTTTGCACAAACT   1620
753        CTATTAAAAAGCACCACTGATTTTAAAAGTGAGAGTGACACAATTATCTTTGCACAAACT   1620
720        CTATTAAAAAGCACCACTGATTTTAAAAGTGAGAGTGACACAATTATCTTTGCACAAACT   1620
47         CTATTAAAAAGCACCACTGATTTTAAAAGTGAGAGTGACACAATTATCTTTGCACAAACT   1620
663        CTATTAAAAAGCACCACTGATTTTAAAAGTGAGAGTGACACAATTATCTTTGCACAAACT   1620
1014       CTATTAAAAAGCACCACTGATTTTAAAAGTGAGAGTGACACAATTATCTTTGCACAAACT   1620
L43967     CTATTAAAAAGCACCACTGATTTTAAAAGTGAGAGTGACACAATTATCTTTGCACAAACT   1620
656        CTATTAAAAAGCACCACTGATTTTAAAAGTGAGAGTGACACAATTATCTTTGCACAAACT   1620
           ************************************************************

719        ATTGCTAATACCGACCAAATTTTTATTGTCACTTCACTAGGTAACATTATTAATATCCCT   1680
20         ATTGCTAATACCGACCAAATTTTTATTGTCACTTCACTAGGTAACATTATTAATATCCCT   1680
735        ATTGCTAATACCGACCAAATTTTTATTGTCACTTCACTAGGTAACATTATTAATATCCCT   1680
649        ATTGCTAATACCGACCAAATTTTTATTGTCACTTCACTAGGTAACATTATTAATATCCCT   1680
710        ATTGCTAATACCGACCAAATTTTTATTGTCACTTCACTAGGTAACATTATTAATATCCCT   1680
729        ATTGCTAATACCGACCAAATTTTTATTGTCACTTCACTAGGTAACATTATTAATATCCCT   1680
38         ATTGCTAATACCGACCAAATTTTTATTGTCACTTCACTAGGTAACATTATTAATATCCCT   1680
600        ATTGCTAATACCGACCAAATTTTTATTGTCACTTCACTAGGTAACATTATTAATATCCCT   1680
122        ATTGCTAATACCGACCAAATTTTTATTGTCACTTCACTAGGTAACATTATTAATATCCCT   1680
683        ATTGCTAATACCGACCAAATTTTTATTGTCACTTCACTAGGTAACATTATTAATATCCCT   1680
1026       ATTGCTAATACCGACCAAATTTTTATTGTCACTTCACTAGGTAACATTATTAATATCCCT   1680
753        ATTGCTAATACCGACCAAATTTTTATTGTCACTTCACTAGGTAACATTATTAATATCCCT   1680
720        ATTGCTAATACCGACCAAATTTTTATTGTCACTTCACTAGGTAACATTATTAATATCCCT   1680
47         ATTGCTAATACCGACCAAATTTTTATTGTCACTTCACTAGGTAACATTATTAATATCCCT   1680
663        ATTGCTAATACCGACCAAATTTTTATTGTCACTTCACTAGGTAACATTATTAATATCCCT   1680
1014       ATTGCTAATACCGACCAAATTTTTATTGTCACTTCACTAGGTAACATTATTAATATCCCT   1680
L43967     ATTGCTAATACCGACCAAATTTTTATTGTCACTTCACTAGGTAACATTATTAATATCCCT   1680
656        ATTGCTAATACCGACCAAATTTTTATTGTCACTTCACTAGGTAACATTATTAATATCCCT   1680
           ************************************************************

719        GTTTATAAATTAGCTTTCAATTCCAAAAATAAACTAGCAAGTTTAGTTAGTAAAAAACCA   1740
20         GTTTATAAATTAGCTTTCAATTCCAAAAATAAACTAGCAAGTTTAGTTAGTAAAAAACCA   1740
735        GTTTATAAATTAGCTTTCAATTCCAAAAATAAACTAGCAAGTTTAGTTAGTAAAAAACCA   1740
649        GTTTATAAATTAGCTTTCAATTCCAAAAATAAACTAGCAAGTTTAGTTAGTAAAAAACCA   1740
710        GTTTATAAATTAGCTTTCAATTCCAAAAATAAACTAGCAAGTTTAGTTAGTAAAAAACCA   1740
729        GTTTATAAATTAGCTTTCAATTCCAAAAATAAACTAGCAAGTTTAGTTAGTAAAAAACCA   1740
38         GTTTATAAATTAGCTTTCAATTCCAAAAATAAACTAGCAAGTTTAGTTAGTAAAAAACCA   1740
600        GTTTATAAATTAGCTTTCAATTCCAAAAATAAACTAGCAAGTTTAGTTAGTAAAAAACCA   1740
122        GTTTATAAATTAGCTTTCAATTCCAAAAATAAACTAGCAAGTTTAGTTAGTAAAAAACCA   1740
683        GTTTATAAATTAGCTTTCAATTCCAAAAATAAACTAGCAAGTTTAGTTAGTAAAAAACCA   1740
1026       GTTTATAAATTAGCTTTCAATTCCAAAAATAAACTAGCAAGTTTAGTTAGTAAAAAACCA   1740
753        GTTTATAAATTAGCTTTCAATTCCAAAAATAAACTAGCAAGTTTAGTTAGTAAAAAACCA   1740
720        GTTTATAAATTAGCTTTCAATTCCAAAAATAAACTAGCAAGTTTAGTTAGTAAAAAACCA   1740
47         GTTTATAAATTAGCTTTCAATTCCAAAAATAAACTAGCAAGTTTAGTTAGTAAAAAACCA   1740
663        GTTTATAAATTAGCTTTCAATTCCAAAAATAAACTAGCAAGTTTAGTTAGTAAAAAACCA   1740
1014       GTTTATAAATTAGCTTTCAATTCCAAAAATAAACTAGCAAGTTTAGTTAGTAAAAAACCA   1740
L43967     GTTTATAAATTAGCTTTCAATTCCAAAAATAAACTAGCAAGTTTAGTTAGTAAAAAACCA   1740
656        GTTTATAAATTAGCTTTCAATTCCAAAAATAAACTAGCAAGTTTAGTTAGTAAAAAACCA   1740
           ************************************************************
```

```
719         ATCCTTTTGGAGTATGAAACGATTGTTTTTGTTGGAACAATAAACAGTGTAAACCAACCA   1800
20          ATCCTTTTGGAGTATGAAACGATTGTTTTTGTTGGAACAATAAACAGTGTAAACCAACCA   1800
735         ATCCTTTTGGAGTATGAAACGATTGTTTTTGTTGGAACAATAAACAGTGTAAACCAACCA   1800
649         ATCCTTTTGGAGTATGAAACGATTGTTTTTGTTGGAACAATAAACAGTGTAAACCAACCA   1800
710         ATCCTTTTGGAGTATGAAACGATTGTTTTTGTTGGAACAATAAACAGTGTAAACCAACCA   1800
729         ATCCTTTTGGAGTATGAAACGATTGTTTTTGTTGGAACAATAAACAGTGTAAACCAACCA   1800
38          ATCCTTTTGGAGTATGAAACGATTGTTTTTGTTGGAACAATAAACAGTGTAAACCAACCA   1800
600         ATCCTTTTGGAGTATGAAACGATTGTTTTTGTTGGAACAATAAACAGTGTAAACCAACCA   1800
122         ATCCTTTTGGAGTATGAAACGATTGTTTTTGTTGGAACAATAAACAGTGTAAACCAACCA   1800
683         ATCCTTTTGGAGTATGAAACGATTGTTTTTGTTGGAACAATGAACAGTGTAAACCAACCA   1800
1026        ATCCTTTTGGAGTATGAAACGATTGTTTTTGTTGGAACAATGAACAGTGTAAACCAACCA   1800
753         ATCCTTTTGGAGTATGAAACGATTGTTTTTGTTGGAACAATGAACAGTGTAAACCAACCA   1800
720         ATCCTTTTGGAGTATGAAACGATTGTTTTTGTTGGAACAATGAACAGTGTAAACCAACCA   1800
47          ATCCTTTTGGAGTATGAAACGATTGTTTTTGTTGGAACAATGAACAGTGTAAACCAACCA   1800
663         ATCCTTTTGGAGTATGAAACGATTGTTTTTGTTGGAACAATGAACAGTGTAAACCAACCA   1800
1014        ATCCTTTTGGAGTATGAAACGATTGTTTTTGTTGGAACAATGAACAGTGTAAACCAACCA   1800
L43967      ATCCTTTTGGAGTATGAAACGATTGTTTTTGTTGGAACAATGAACAGTGTAAACCAACCA   1800
656         ATCCTTTTGGAGTATGAAACGATTGTTTTTGTTGGAACAATGAACAGTGTAAACCAACCA   1800
            ***************************************** ******************

719         ATCCTTGTTTTAACTTCCAAACTAGGAATGGTTAAACGGATTGATTTAACCAAACTTAAC   1860
20          ATCCTTGTTTTAACTTCCAAACTAGGAATGGTTAAACGGATTGATTTAACCAAACTTAAC   1860
735         ATCCTTGTTTTAACTTCCAAACTAGGAATGGTTAAACGGATTGATTTAACCAAACTTAAC   1860
649         ATCCTTGTTTTAACTTCCAAACTAGGAATGGTTAAACGGATTGATTTAACCAAACTTAAC   1860
710         ATCCTTGTTTTAACTTCCAAACTAGGAATGGTTAAACGGATTGATTTAACCAAACTTAAC   1860
729         ATCCTTGTTTTAACTTCCAAACTAGGAATGGTTAAACGGATTGATTTAACCAAACTTAAC   1860
38          ATCCTTGTTTTAACTTCCAAACTAGGAATGGTTAAACGGATTGATTTAACCAAACTTAAC   1860
600         ATCCTTGTTTTAACTTCCAAACTAGGAATGGTTAAACGGATTGATTTAACCAAACTTAAC   1860
122         ATCCTTGTTTTAACTTCCAAACTAGGAATGGTTAAACGGATTGATTTAACCAAACTTAAC   1860
683         ATCCTTGTTTTAACTTCCAAACTAGGAATGGTTAAACGGATTGATTTAACCAAACTTAAC   1860
1026        ATCCTTGTTTTAACTTCCAAACTAGGAATGGTTAAACGGATTGATTTAACCAAACTTAAC   1860
753         ATCCTTGTTTTAACTTCCAAACTAGGAATGGTTAAACGGATTGATTTAACCAAACTTAAC   1860
720         ATCCTTGTTTTAACTTCCAAACTAGGAATGGTTAAACGGATTGATTTAACCAAACTTAAC   1860
47          ATCCTTGTTTTAACTTCCAAACTAGGAATGGTTAAACGGATTGATTTAACCAAACTTAAC   1860
663         ATCCTTGTTTTAACTTCCAAACTAGGAATGGTTAAACGGATTGATTTAACCAAACTTAAC   1860
1014        ATCCTTGTTTTAACTTCCAAACTAGGAATGGTTAAACGGATTGATTTAACCAAACTTAAC   1860
L43967      ATCCTTGTTTTAACTTCCAAACTAGGAATGGTTAAACGGATTGATTTAACCAAACTTAAC   1860
656         ATCCTTGTTTTAACTTCCAAACTAGGAATGGTTAAACGGATTGATTTAACCAAACTTAAC   1860
            ************************************************************

719         AATAAGCCACTTAAAGCTACTTTGTGTATCTCACTCCGTGATAAAGACCAGTTAGTAAGT   1920
20          AATAAGCCACTTAAAGCTACTTTGTGTATCTCACTCCGTGATAAAGACCAGTTAGTAAGT   1920
735         AATAAGCCACTTAAAGCTACTTTGTGTATCTCACTCCGTGATAAAGACCAGTTAGTAAGT   1920
649         AATAAGCCACTTAAAGCTACTTTGTGTATCTCACTCCGTGATAAAGACCAGTTAGTAAGT   1920
710         AATAAGCCACTTAAAGCTACTTTGTGTATCTCACTCCGTGATAAAGACCAGTTAGTAAGT   1920
729         AATAAGCCACTTAAAGCTACTTTGTGTATCTCACTCCGTGATAAAGACCAGTTAGTAAGT   1920
38          AATAAGCCACTTAAAGCTACTTTGTGTATCTCACTCCGTGATAAAGACCAGTTAGTAAGT   1920
600         AATAAGCCACTTAAAGCTACTTTGTGTATCTCACTCCGTGATAAAGACCAGTTAGTAAGT   1920
122         AATAAGCCACTTAAAGCTACTTTGTGTATCTCACTCCGTGATAAAGACCAGTTAGTAAGT   1920
683         AATAAGCCACTTAAAGCTACTTTGTGTATCTCACTCCGTGATAAAGACCAGTTAGTAAGT   1920
1026        AATAAGCCACTTAAAGCTACTTTGTGTATCTCACTCCGTGATAAAGACCAGTTAGTAAGT   1920
753         AATAAGCCACTTAAAGCTACTTTGTGTATCTCACTCCGTGATAAAGACCAGTTAGTAAGT   1920
720         AATAAGCCACTTAAAGCTACTTTGTGTATCTCACTCCGTGATAAAGACCAGTTAGTAAGT   1920
47          ATTAAGCCACTTAAAGCTACTTTGTGTATCTCACTCCGTGATAAAGACCATTTAGTAAGT   1920
663         ATTAAGCCACTTAAAGCTACTTTGTGTATCTCACTCCGTGATAAAGACCATTTAGTAAGT   1920
1014        ATTAAGCCACTTAAAGCTACTTTGTGTATCTCACTCCGTGATAAAGACCATTTAGTAAGT   1920
L43967      ATTAAGCCACTTAAAGCTACTTTGTGTATCTCACTCCGTGATAAAGACCATTTAGTAAGT   1920
656         ATTAAGCCACTTAAAGCTACTTTGTGTATCTCACTCCGTGATAAAGACCATTTAGTAAGT   1920
            * ************************************************ *********

719         GCATTTTTACAACAAGATGATAAACTGATCTGTTTAGTGTCTGATCACAACTATTACACA   1980
20          GCATTTTTACAACAAGATGATAAACTGATCTGTTTAGTGTCTGATCACAACTATTACACA   1980
735         GCATTTTTACAACAAGATGATAAACTGATCTGTTTAGTGTCTGATCACAACTATTACACA   1980
649         GCATTTTTACAACAAGATGATAAACTGATCTGTTTAGTGTCTGATCACAACTATTACACA   1980
710         GCATTTTTACAACAAGATGATAAACTGATCTGTTTAGTGTCTGATCACAACTATTACACA   1980
729         GCATTTTTACAACAAGATGATAAACTGATCTGTTTAGTGTCTGATCACAACTATTACACA   1980
38          GCATTTTTACAACAAGATGATAAACTGATCTGTTTAGTGTCTGATCACAACTATTACACA   1980
600         GCATTTTTACAACAAGATGATAAACTGATCTGTTTAGTGTCTGATCACAACTATTACACA   1980
122         GCATTTTTACAACAAGATGATAAACTGATCTGTTTAGTGTCTGATCACAACTATTACACA   1980
683         GCATTTTTACAACAAGATGATAAACTGATCTGTTTAGTGTCTGATCACAACTATTACACA   1980
1026        GCATTTTTACAACAAGATGATAAACTGATCTGTTTAGTGTCTGATCACAACTATTACACA   1980
753         GCATTTTTACAACAAGATGATAAACTGATCTGTTTAGTGTCTGATCACAACTATTACACA   1980
720         GCATTTTTACAACAAGATGATAAACTGATCTGTTTAGTGTCTGATCACAACTATTACACA   1980
47          GCATTTTTACAACAAGATGATAAACTGATCTGTTTAGTGTCTGATCACAACTATTACACT   1980
663         GCATTTTTACAACAAGATGATAAACTGATCTGTTTAGTGTCTGATCACAACTATTACACT   1980
1014        GCATTTTTACAACAAGATGATAAACTGATCTGTTTAGTGTCTGATCACAACTATTACACT   1980
L43967      GCATTTTTACAACAAGATGATAAACTGATCTGTTTAGTGTCTGATCACAACTATTACACT   1980
656         GCATTTTTACAACAAGATGATAAACTGATCTGTTTAGTGTCTGATCACAACTATTACACT   1980
            ***********************************************************

719         GTTTTTCACACCAATGAGATCCCACTAATTAGTAGTAAGGGGATGGGAGTGAAGGGGATG   2040
20          GTTTTTCACACCAATGAGATCCCACTAATTAGTAGTAAGGGGATGGGAGTGAAGGGGATG   2040
```

```
735       GTTTTTCACACCAATGAGATCCCACTAATTAGTAGTAAGGGGATGGGAGTGAAGGGGATG   2040
649       GTTTTTCACACCAATGAGATCCCACTAATTAGTAGTAAGGGGATGGGAGTGAAGGGGATG   2040
710       GTTTTTCACACCAATGAGATCCCACTAATTAGTAGTAAGGGGATGGGAGTGAAGGGGATG   2040
729       GTTTTTCACACCAATGAGATCCCACTAATTAGTAGTAAGGGGATGGGAGTGAAGGGGATG   2040
38        GTTTTTCACACCAATGAGATCCCACTAATTAGTAGTAAGGGGATGGGAGTGAAGGGGATG   2040
600       GTTTTTCACACCAATGAGATCCCACTAATTAGTAGTAAGGGGATGGGAGTGAAGGGGATG   2040
122       GTTTTTCACACCAATGAGATCCCACTAATTAGTAGTGAGGGGATGGGAGTGAAGGGGATG   2040
683       GTTTTTCACACCAATGAGATCCCATTAATTAGTAGTAAGGGGATGGGAGTGAAGGGGATG   2040
1026      GTTTTTCACACCAATGAGATCCCATTAATTAGTAGTAAGGGGATGGGAGTGAAGGGGATG   2040
753       GTTTTTCACACCAATGAGATCCCATTAATTAGTAGTAAGGGGATGGGAGTGAAGGGGATG   2040
720       GTTTTTCACACCAATGAGATCCCATTAATTAGTAGTAAGGGGATGGGAGTGAAGGGGATG   2040
47        GTTTTTCACACCAATGAGATCCCATTAATTAGTAGTAAGGGGATGGGAGTGAAGGGGATG   2040
663       GTTTTTCACACCAATGAGATCCCATTAATTAGTAGTAAGGGGATGGGAGTGAAGGGGATG   2040
1014      GTTTTTCACACCAATGAGATCCCATTAATTAGTAGTAAGGGGATGGGAGTGAAGGGGATG   2040
L43967    GTTTTTCACACCAATGAGATCCCATTAATTAGTAGTAAGGGGATGGGAGTGAAGGGGATG   2040
656       GTTTTTCACACCAATGAGATCCCATTAATTAGTAGTAAGGGGATGGGAGTGAAGGGGATG   2040
          ************************ *********** ***********************

719       AAGTTAAAACTAGAGGATCAAATTAAGTTTGTTGTTGCTTTTGAAGCTAATGAACCGTTA   2100
20        AAGTTAAAACTAGAGGATCAAATTAAGTTTGTTGTTGCTTTTGAAGCTAATGAACCGTTA   2100
735       AAGTTAAAACTAGAGGATCAAATTAAGTTTGTTGTTGCTTTTGAAGCTAATGAACCGTTA   2100
649       AAGTTAAAACTAGAGGATCAAATTAAGTTTGTTGTTGCTTTTGAAGCTAATGAACCGTTA   2100
710       AAGTTAAAACTAGAGGATCAAATTAAGTTTGTTGTTGCTTTTGAAGCTAATGAACCGTTA   2100
729       AAGTTAAAACTAGAGGATCAAATTAAGTTTGTTGTTGCTTTTGAAGCTAATGAACCGTTA   2100
38        AAGTTAAAACTAGAGGATCAAATTAAGTTTGTTGTTGCTTTTGAAGCTAATGAACCGTTA   2100
600       AAGTTAAAACTAGAGGATCAAATTAAGTTTGTTGTTGCTTTTGAAGCTAATGAACCGTTA   2100
122       AAGTTAAAACTAGAGGATCAAATTAAGTTTGTTGTTGCTTTTGAAGCTAATGAACCGTTA   2100
683       AAGTTAAAACTAGAGGATCAAATTAAGTTTGTTGTTGCTTTTGAAGCTAATGAACCGTTA   2100
1026      AAGTTAAAACTAGAGGATCAAATTAAGTTTGTTGTTGCTTTTGAAGCTAATGAACCGTTA   2100
753       AAGTTAAAACTAGAGGATCAAATTAAGTTTGTTGTTGCTTTTGAAGCTAATGAACCGTTA   2100
720       AAGTTAAAACTAGAGGATCAAATTAAGTTTGTTGTTGCTTTTGAAGCTAATGAACCGTTA   2100
47        AAGTTAAAACTAGAGGATCAAATTAAGTTTGTTGTTGCTTTTGAAGCTAATGAACCGTTA   2100
663       AAGTTAAAACTAGAGGATCAAATTAAGTTTGTTGTTGCTTTTGAAGCTAATGAACCGTTA   2100
1014      AAGTTAAAACTAGAGGATCAAATTAAGTTTGTTGTTGCTTTTGAAGCTAATGAACCGTTA   2100
L43967    AAGTTAAAACTAGAGGATCAAATTAAGTTTGTTGTTGCTTTTGAAGCTAATGAACCGTTA   2100
656       AAGTTAAAACTAGAGGATCAAATTAAGTTTGTTGTTGCTTTTGAAGCTAATGAACCGTTA   2100
          ************************************************************

719       GCGATGATATGTAGTGATGGTAGTGTCATTAACTTAAAACAAACTGAACTAGTTGTAGTT   2160
20        GCGATGATATGTAGTGATGGTAGTGTCATTAACTTAAAACAAACTGAACTAGTTGTAGTT   2160
735       GCGATGATATGTAGTGATGGTAGTGTCATTAACTTAAAACAAACTGAACTAGTTGTAGTT   2160
649       GTGATGATATGTAGTGATGGTAGTGTCATTAACTTAAAACAAACTGAACTAGTTGTAGTT   2160
710       GTGATGATATGTAGTGATGGTAGTGTCATTAACTTAAAACAAACTGAACTAGTTGTAGTT   2160
729       GTGATGATATGTAGTGATGGTAGTGTCATTAACTTAAAACAAACTGAACTAGTTGTAGTT   2160
38        GTGATGATATGTAGTGATGGTAGTGTCATTAACTTAAAACAAACTGAACTAGTTGTAGTT   2160
600       GTGATGATATGTAGTGATGGTAGTGTCATTAACTTAAAACAAACTGAACTAGTTGTAGTT   2160
122       GTGATGATATGTAGTGATGGTAGTGTCATTAACTTAAAACAAACTGAACTAGTTGTAGTT   2160
683       GTGATGATATGTAGTGATGGTAGTGTCATTAACTTAAAACAAACTGAACTAGTTGTAGTT   2160
1026      GTGATGATATGTAGTGATGGTAGTGTCATTAACTTAAAACAAACTGAACTAGTTGTAGTT   2160
753       GTGATGATATGTAGTGATGGTAGTGTCATTAACTTAAAACAAACTGAACTAGTTGTAGTT   2160
720       GTGATGATATGTAGTGATGGTAGTGTCATTAACTTAAAACAAACTGAACTAGTTGTAGTT   2160
47        GTGATGATATGTAGTGATGGTAGTGTCATTAACTTAAAACAAACTGAACTAGTTGTAGTT   2160
663       GTGATGATATGTAGTGATGGTAGTGTCATTAACTTAAAACAAACTGAACTAGTTGTAGTT   2160
1014      GTGATGATATGTAGTGATGGTAGTGTCATTAACTTAAAACAAACTGAACTAGTTGTAGTT   2160
L43967    GTGATGATATGTAGTGATGGTAGTGTCATTAACTTAAAACAAACTGAACTAGTTGTAGTT   2160
656       GTGATGATATGTAGTGATGGTAGTGTCATTAACTTAAAACGAACTGAACTAGTTGTAGTT   2160
          * ************************************** *******************

719       AGCAGGATGGCAACTGCAAAAAAACTGCCTGTTAAGAAAGCAATTAACTATTGTTTTAGT   2220
20        AGCAGGATGGCAACTGCAAAAAAACTGCCTGTTAAGAAAGCAATTAACTATTGTTTTAGT   2220
735       AGCAGGATGGCAACTGCAAAAAAACTGCCTGTTAAGAAAGCAATTAACTATTGTTTTAGT   2220
649       AGCAGGATGGCAACTGCAAAAAAACTGCCTGTTAAGAAAGCAATTAACTATTGTTTTAGT   2220
710       AGCAGGATGGCAACTGCAAAAAAACTGCCTGTTAAGAAAGCAATTAACTATTGTTTTAGT   2220
729       AGCAGGATGGCAACTGCAAAAAAACTGCCTGTTAAGAAAGCAATTAACTATTGTTTTAGT   2220
38        AGCAGGATGGCAACTGCAAAAAAACTGCCTGTTAAGAAAGCAATTAACTATTGTTTTAGT   2220
600       AGCAGGATGGCAACTGCAAAAAAACTGCCTGTTAAGAAAGCAATTAACTATTGTTTTAGT   2220
122       AGCAGGATGGCAACTGCAAAAAAACTGCCTGTTAAGAAAGCAATTAACTATTGTTTTAGT   2220
683       AGCAGGATGGCAACTGCAAAAAAACTGCCTGTTAAGAAAGCAATTAACTATTGTTTTAGT   2220
1026      AGCAGGATGGCAACTGCAAAAAAACTGCCTGTTAAGAAAGCAATTAACTATTGTTTTAGT   2220
753       AGCAGGATGGCAACTGCAAAAAAACTGCCTGTTAAGAAAGCAATTAACTATTGTTTTAGT   2220
720       AGCAGGATGGCAACTGCAAAAAAACTGCCTGTTAAGAAAGCAATTAACTATTGTTTTAGT   2220
47        AGCAGGATGGCAACTGCAAAAAAACTGCCTGTTAAGAAAGCAATTAACTATTGTTTTAGT   2220
663       AGCAGGATGGCAACTGCAAAAAAACTGCCTGTTAAGAAAGCAATTAACTATTGTTTTAGT   2220
1014      AGCAGGATGGCAACTGCAAAAAAACTGCCTGTTAAGAAAGCAATTAACTATTGTTTTAGT   2220
L43967    AGCAGGATGGCAACTGCAAAAAAACTGCCTGTTAAGAAAGCAATTAACTATTGTTTTAGT   2220
656       AGCAGGATGGCAACTGCAAAAAAACTGCCTGTTAAGAAAGCAATTAACTATTGTTTTAGT   2220
          ************************************************************

719       GATGCAACTAACACCCAGTTAATTAATTTTCAGGGTAAGAACGGTAGTAAATTAATTACA   2280
20        GATGCAACTAACACCCAGTTAATTAATTTTCAGGGTAAGAACGGTAGTAAATTAATTACA   2280
735       GATGCAACTAACACCCAGTTAATTAATTTTCAGGGTAAGAACGGTAGTAAATTAATTACA   2280
649       GATGCAACTAACACCCAGTTAATTAATTTTCAGGGTAAGAACGGTAGTAAATTAATTACA   2280
710       GATGCAACTAACACCCAGTTAATTAATTTTCAGGGTAAGAACGGTAGTAAATTAATTACA   2280
```

```
729        GATGCAACTAACACCCAGTTAATTAATTTTCAGGGTAAGAACGGTAGTAAATTAATTACA   2280
38         GATGCAACTAACACCCAGTTAATTAATTTTCAGGGTAAGAACGGTAGTAAATTAATTACA   2280
600        GATGCAACTAACACCCAGTTAATTAATTTTCAGGGTAAGAACGGTAGTAAATTAATTACA   2280
122        GATGCAACTAACACCCAGTTAATTAATTTTCAGGGTAAGAACGGTAGTAAATTAATTACA   2280
683        GATGCAACTAACACCCAGTTAATTAATTTTCAGGGTAAGAATGGTAGTAAATTAATTACA   2280
1026       GATGCAACTAACACCCAGTTAATTAATTTTCAGGGTAAGAATGGTAGTAAATTAATTACA   2280
753        GATGCAACTAACACCCAGTTAATTAATTTTCAGGGTAAGAATGGTAGTAAATTAATTACA   2280
720        GATGCAACTAACACCCAGTTAATTAATTTTCAGGGTAAGAATGGTAGTAAATTAATTACA   2280
47         GATGCAACTAACACCCAGTTAATTAATTTTCAGGGTAAGAACGGTAGTAAATTAATTACA   2280
663        GATGCAACTAACACCCAGTTAATTAATTTTCAGGGTAAGAACGGTAGTAAATTAATTACA   2280
1014       GATGCAACTAACACCCAGTTAATTAATTTTCAGGGTAAGAACGGTAGTAAATTAATTACA   2280
L43967     GATGCAACTAACACCCAGTTAATTAATTTTCAGGGTAAGAACGGTAGTAAATTAATTACA   2280
656        GATGCAACTAACACCCAGTTAATTAATTTTCAGGGTAAGAACGGTAGTAAATTAATTACA   2280
           ***************************************** ******************

719        ACTAGTGAACTGAACCAGATGAGTAAAACTGCAATTAGTCAAACCAGGTTTAACAAACTT   2340
20         ACTAGTGAACTGAACCAGATGAGTAAAACTGCAATTAGTCAAACCAGGTTTAACAAACTT   2340
735        ACTAGTGAACTGAACCAGATGAGTAAAACTGCAATTAGTCAAACCAGGTTTAACAAACTT   2340
649        ACTAGTGAACTGAACCAGATGAGTAAAACTGCAATTAGTCAAACCAGGTTTAACAAACTT   2340
710        ACTAGTGAACTGAACCAGATGAGTAAAACTGCAATTAGTCAAACCAGGTTTAACAAACTT   2340
729        ACTAGTGAACTGAACCAGATGAGTAAAACTGCAATTAGTCAAACCAGGTTTAACAAACTT   2340
38         ACTAGTGAACTGAACCAGATGAGTAAAACTGCAATTAGTCAAACCAGGTTTAACAAACTT   2340
600        ACTAGTGAACTGAACCAGATGAGTAAAACTGCAATTAGTCAAACCAGGTTTAACAAACTT   2340
122        ACTAGTGAACTGAACCAGATGAGTAAAACTGCAATTAGTCAAACCAGGTTTAACAAACTT   2340
683        ACTAGTGAACTGAACCAGATGAGTAAAACTGCAATTAGTCAAACCAGGTTTAACAAACTT   2340
1026       ACTAGTGAACTGAACCAGATGAGTAAAACTGCAATTAGTCAAACCAGGTTTAACAAACTT   2340
753        ACTAGTGAACTGAACCAGATGAGTAAAACTGCAATTAGTCAAACCAGGTTTAACAAACTT   2340
720        ACTAGTGAACTGAACCAGATGAGTAAAACTGCAATTAGTCAAACCAGGTTTAACAAACTT   2340
47         ACTAGTGAACTGAACCAGATGAGTAAAACTGCAATTAGTCAAACCAGGTTTAACAAACTT   2340
663        ACTAGTGAACTGAACCAGATGAGTAAAACTGCAATTAGTCAAACCAGGTTTAACAAACTT   2340
1014       ACTAGTGAACTGAACCAGATGAGTAAAACTGCAATTAGTCAAACCAGGTTTAACAAACTT   2340
L43967     ACTAGTGAACTGAACCAGATGAGTAAAACTGCAATTAGTCAAACCAGGTTTAACAAACTT   2340
656        ACTAGTGAACTGAACCAGATGAGTAAAACTGCAATTAGTCAAACCAGGTTTAACAAACTT   2340
           ************************************************************

719        AATTAG    2346
20         AATTAG    2346
735        AATTAG    2346
649        AATTAG    2346
710        AATTAG    2346
729        AATTAG    2346
38         AATTAG    2346
600        AATTAG    2346
122        AATTAG    2346
683        AATTAG    2346
1026       AATTAG    2346
753        AATTAG    2346
720        AATTAG    2346
47         AATTAG    2346
663        AATTAG    2346
1014       AATTAG    2346
L43967     AATTAG    2346
656        AATTAG    2346
           ******
```
